# An Unusual Oxidative Rearrangement Catalyzed by a Divergent Member of the 2‐Oxoglutarate‐Dependent Dioxygenase Superfamily during Biosynthesis of Dehydrofosmidomycin

**DOI:** 10.1002/anie.202206173

**Published:** 2022-06-07

**Authors:** Elizabeth I. Parkinson, Hani G. Lakkis, Amir A. Alwali, Mary Elizabeth M. Metcalf, Ramya Modi, William W. Metcalf

**Affiliations:** ^1^ Institute for Genomic Biology University of Illinois at Urbana-Champaign 1206 W. Gregory Dr. Urbana IL 61801 USA; ^2^ Department of Chemistry Purdue University Herbert C. Brown Laboratory of Chemistry, Room 4103E 560 Oval Drive, Box 59 West Lafayette IN 47907 USA; ^3^ Department of Medicinal Chemistry and Molecular Pharmacology Purdue University Herbert C. Brown Laboratory of Chemistry, Room 4103E 560 Oval Drive, Box 59 West Lafayette IN 47907 USA; ^4^ Department of Microbiology University of Illinois at Urbana-Champaign, B103C&LSL 601 S. Goodwin Urbana IL 61801 USA

**Keywords:** Biosynthesis, Enzyme Catalysis, Natural Products, Phosphonates

## Abstract

The biosynthesis of the natural product dehydrofosmidomycin involves an unusual transformation in which 2‐(trimethylamino)ethylphosphonate is rearranged, desaturated and demethylated by the enzyme DfmD, a divergent member of the 2‐oxoglutarate‐dependent dioxygenase superfamily. Although other members of this enzyme family catalyze superficially similar transformations, the combination of all three reactions in a single enzyme has not previously been observed. By characterizing the products of in vitro reactions with labeled and unlabeled substrates, we show that DfmD performs this transformation in two steps, with the first involving desaturation of the substrate to form 2‐(trimethylamino)vinylphosphonate, and the second involving rearrangement and demethylation to form methyldehydrofosmidomycin. These data reveal significant differences from the desaturation and rearrangement reactions catalyzed by other family members.

## Graphical abstract

DfmD is a highly divergent member of the 2‐oxoglutarate‐dependent dioxygenase superfamily that catalyzes desaturation, rearrangement and demethylation of its substrate. In vitro biochemical studies and homology modeling suggest that these transformations occur via novel chemistry.

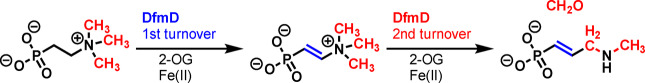

## Introduction

Organophosphonic acids, characterized by the presence of stable carbon‐phosphorus (C−P) bonds, are produced by approximately 5 % of microbes, including a wide variety of bacteria, fungi and protists.^[1]^ Members of this diverse molecular family often display potent bioactivity due to their chemical mimicry of common cellular metabolites.^[2]^ Among these are the clinically used antibiotic fosfomycin, an analog of phosphoenolpyruvate (PEP); the potent herbicide phosphinothricin, an analog of glutamate; and the fosmidomycin family of antimalarial compounds, which mimic the isoprenoid precursor deoxyxyluose‐phosphate. Despite the structural diversity of phosphonate natural products, the biosynthesis of nearly all of these molecules begins with the rearrangement of PEP to phosphonopyruvate, catalyzed by the enzyme PEP mutase.^[2]^ The biosynthetic pathways that convert this early intermediate into disparate bioactive products have now been characterized for numerous phosphonate natural products. Because many phosphonate biosynthetic intermediates are also similar to common metabolites, it is not surprising that the enzymes which produce these intermediates are evolutionarily derived from enzymes of central metabolism.^[2]^ Nevertheless, it is also clear that standard biochemistry is insufficient for many of the transformations involved in these pathways. Indeed, a number of unusual and often unprecedented enzymatic transformations have been discovered during characterization of phosphonate biosynthesis and catabolism.^[3]^ Among these are a family of related peroxidases and oxygenases involved in the synthesis of fosfomycin,^[4]^ phosphinothricin^[5]^ and methylphosphonate;^[6]^ an oxygen‐ and iron‐dependent decarboxylase involved in the *Pseudomonas* pathway for fosfomycin biosynthesis,^[7]^ an oxaloacetate‐dependent retroaldolase in rhizocticin biosynthesis,^[8]^ a series of tRNA‐dependent peptide‐forming enzymes needed for production of dehydrophos^[9]^ and the carbon‐phosphorus lyase involved in catabolism of numerous phosphonates.^[10]^ Our recent studies have added a new reaction to this list, namely an unusual oxidative rearrangement involved in the biosynthesis of the phosphonate natural product dehydrofosmidomycin.^[11]^

Dehydrofosmidomycin is one of a family of related phosphonate molecules that inhibit deoxylulose‐5‐phosphate reductoisomerase (Dxr), an enzyme which catalyzes a key step in the bacterial pathway for biosynthesis of the isoprenoid precursors isopentenyl‐pyrophosphate (IPP) and dimethyallylpyrophosphate (DMAP).^[11]^ Although all life forms require IPP and DMAP, the biosynthetic pathways used to produce these precursors in bacteria differ significantly from those used by most archaea and eukaryotes. Thus, Dxr inhibitors are potent antibacterial agents with minimal toxicity towards humans. Interestingly, because of their dependence on bacterially derived organelles (chloroplasts and apicoplasts) that use the bacterial pathway for synthesis of IPP and DMAP, dehydrofosmidomycin and its congeners also have potent herbicidal and antimalarial activity.^[12]^

Strong interest in the diverse bioactivities of phosphonate Dxr inhibitors has motivated studies on the biosynthesis of dehydrofosmidomycin and its equally potent congener FR‐900098. Surprisingly, the biosynthesis of dehydrofosmidomycin diverges significantly from that of FR‐900098, suggesting that biosynthesis of this potent pharmacophore has evolved independently at least twice.[^11^, ^13^] While both pathways begin with PEP mutase, FR‐900098 is derived from a five‐carbon intermediate that is shortened by successive decarboxylation reactions, whereas dehydrofosmidomycin is made from a two‐carbon intermediate that is lengthened by a one carbon.[^1^, ^13^, ^14^] In vivo labeling studies in the dehydrofosmidomycin producer *Streptomyces lavendulae*, suggest that this one‐carbon unit is derived from *S*‐adenosylmethionine (SAM) and involves the intermediate 2‐(trimethylamino)ethlyphosphonate (TMAEP). In vitro biochemistry shows that conversion of this saturated two‐carbon intermediate to an unsaturated three‐carbon intermediate is catalyzed by the enzyme DfmD. Although the details of this unusual oxidative rearrangement have yet to be established, our initial biochemical and bioinformatic analyses show that DfmD is a member of the 2‐oxoglutarate (2OG)‐dependent dioxygenase superfamily.^[11]^

**Figure 1:**
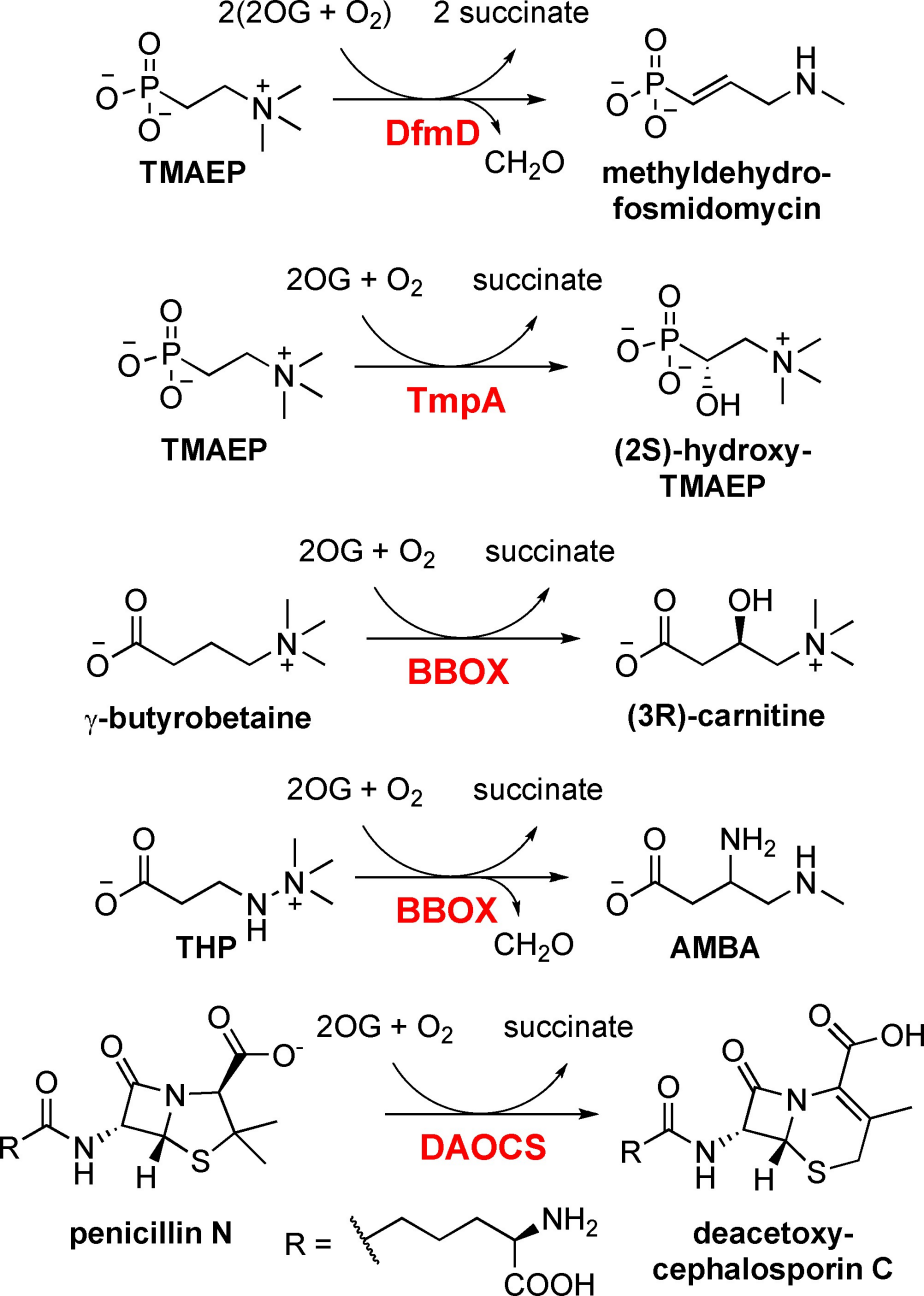
Reactions catalyzed by 2‐oxoglutarate‐dependent dioxygenases. The following reactions are shown in order from top to bottom: The oxidative rearrangement of trimethylaminoethylphosphonate (TMAEP) catalyzed by DfmD during biosynthesis of dehydrofosmidomycin. The hydroxylation of TMAEP catalyzed by TmpA during catabolism of the substrate. The standard hydroxylation reaction involved in carnatine biosynthesis catalyzed by γ‐butyrobetaine hydroxylase (BBOX). The BBOX‐catalyzed rearrangement of the inhibitor trimethylhydrazine‐propionate (THP, also known as meldonium). The rearrangement and desaturation of penicillin N‐catalyzed by deactoxycephalosporin synthase (DAOCS). Abbreviations used: 2‐oxoglutarate (2OG),1‐hydroxy‐2‐trimethylaminoethylphosphonate (hydroxy‐TMAEP), 3‐amino‐4‐(methylamino)butyrate (AMBA).

2OG‐dependent dioxygenases comprise one of the largest protein superfamilies, being found in bacteria, archaea, eukayotes and even viruses.^[14]^ The most common reaction catalyzed by members of this family is hydroxylation, exemplified by the well‐studied enzyme taurine hydroxylase (TauD);^[15]^ however, a wide variety of reactions can be performed by these proteins including desaturations, halogenations, epimerizations, as well as ring expansions and closures.^[16]^ Members of this family require ferrous iron for activity and use 2OG and molecular oxygen as co‐substrates. Catalyzed reactions typically involve two‐electron oxidations of both the substrate and 2OG, with the latter being oxidized to succinate and CO_2_. The precise mechanism depends on the type of reaction being catalyzed and most, if not all, involve generation of substrate radicals via an Fe^IV^‐oxo intermediate. The putative DfmD reaction involves both desaturation and an unusual rearrangement of TMAEP, with insertion of one of the *N*‐methyl groups as a methylene moiety in the product methyldehydrofosmidomycin and release of another as formaldehyde (Figure 1).^[11]^ Although there are a small number of 2OG‐dependent dioxygenases enzymes that catalyze rearrangements and desaturations, all examples of which we are aware have significant differences from DfmD (Figure 1). Of particular interest is an adventitious reaction catalyzed by γ‐butyrobetaine hydroxylase (BBOX), which normally performs a standard hydroxylation reaction during carnitine biosynthesis.^[17]^ When incubated with trimethylhydrazine‐propionate (THP), a BBOX inhibitor used to treat cardiovascular disease, this enzyme catalyzes a DfmD‐like rearrangement coupled with formaldehyde release. However, unlike DfmD, BBOX does not perform desaturation. Another superficially similar reaction is that of deacetoxycephalosporin C synthase (DAOCS) from *Streptomyces clavuligerus*, which catalyzes both rearrangement and desaturation of its substrate.^[18]^ However, DOACS differs from DfmD in that the methylene insertion forms a carbon‐sulfur, rather than carbon‐carbon bond, and does not release formaldehyde. Finally, it is worth noting that TmpA, used by *Leisingera caerula* for catabolism of TMAEP (the same substrate as used by DfmD), catalyzes a simple hydroxylation without desaturation or rearrangement.^[19]^

To better understand the unusual reaction catalyzed by DfmD, as well as the similarities and differences between it and other members of the 2OG‐dioxygenase superfamily, we performed in vitro studies of the enzyme using labelled and unlabeled substrates. We also conducted a series of bioinformatics analyses that provide insight into its structure and evolution. Our data show that DfmD is highly diverged from all known family members and that the unusual desaturation and rearrangement reactions occur via discrete steps in a multi‐turnover reaction pathway.

## Results and Discussion

Although we previously showed that DfmD converts TMAEP to methyldehydrofosmidomycin and formaldehyde, the stoichiometry and components required for the reaction have yet to be fully characterized.^[11]^ To address this, we conducted a series of in vitro reactions using recombinant 6‐his‐tagged DfmD.

**Figure 2:**
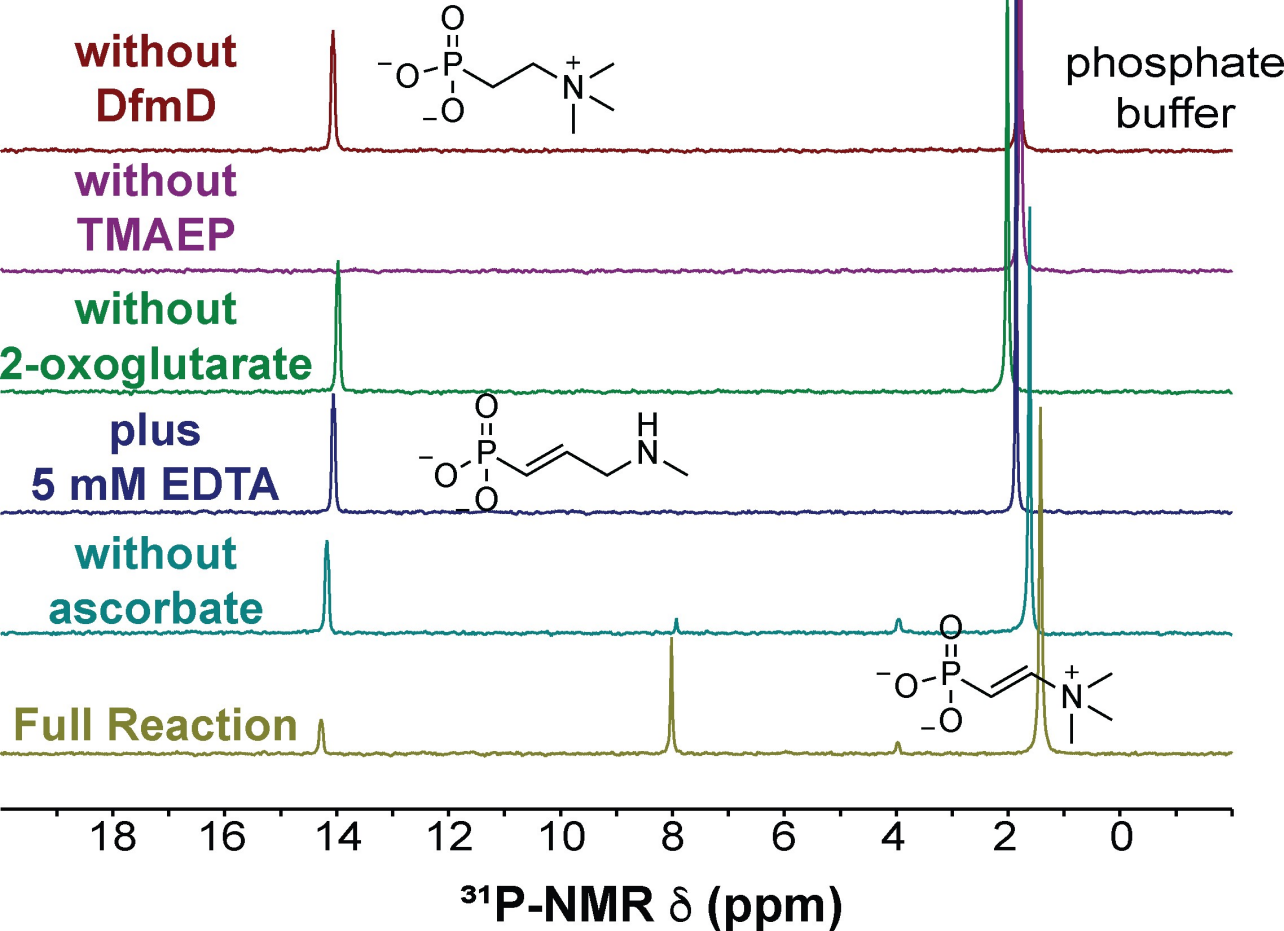
DfmD reaction requirements. Requirements for the reaction were determined by systemic exclusion or addition of certain components. For the full reaction (yellow), DfmD (5 μM) was incubated with 2‐oxoglutarate (10 mM), L‐ascorbate (5 mM), TMAEP (5 mM), and Fe^II^ (0.2 mM) for 24 h at 28 °C. EDTA (5 mM) was added to chelate the Fe^II^ and demonstrate the dependence on Fe^II^. Observed product peaks are labelled with the compounds that produce each signal (see Figures S1 and S2 for validation). Triplicate experiments produced qualitatively similar results; representative data from a single experiment is shown.

Based on ^31^P‐NMR monitoring of the phosphonate substrates and products, DfmD converts TMAEP to methyldehydrofosmidomycin in a reaction that requires both Fe^II^ and 2‐oxoglutarate (Figures 2 and S1). Like other 2‐OG‐dependent dioxygenases this reaction is stimulated by addition of the reducing agent ascorbic acid.^[20]^ During the course of the reaction, an additional P‐containing product with a chemical shift in the range typical for phosphonic acids was observed in the full reaction and in the no ascorbate control. A time course shows that this minor product appears first and remains at a low concentration, while the relative concentrations of TMAEP and methyldehydrofosmidomycin decrease and increase, respectively, consistent with this molecule being an intermediate in the reaction (Figure S2A). ^1^H‐^31^P‐HMBC‐NMR analysis of the reaction shows that the putative intermediate has a vinyl moiety adjacent to the C−P bond (Figure S2B), while high‐resolution LC‐MS/MS analysis of the reaction revealed exact masses consistent with the presence of the substrate and product, as well as a third compound with a likely empirical formula of C_5_H_12_NO_3_P (Figure S2C). Four isobaric structures, namely the *cis* and *trans* isomers of dimethyldehydrofosmidomycin and 2‐(trimethylamino)vinylphosphonate (TMAVP), can reasonably be proposed that are consistent with these data. Based on the *trans* configuration of the final product, we did not consider the *cis* isomers of these compounds to be likely candidates. To distinguish between the two *trans* isomers, we synthesized *trans*‐dimethyldehydrofosmidomycin and *trans*‐TMAVP and spiked them into DfmD reactions. Addition of dimethyldehydrofosmidomycin produced a new ^31^P‐NMR peak just upfield of the putative intermediate (Figure S2D). In contrast, spiking with TMAVP did not produce a new peak, but instead increased the size of the peak assigned to the putative intermediate (Figure S2E). Thus, the putative intermediate can confidently be assigned as *trans*‐TMAVP (hereafter simply TMAVP). Although the time course data suggest that TMAVP is an intermediate in the DfmD reaction, it is remains possible that it is an off‐pathway shunt product. To test this, we incubated DfmD with TMAVP and monitored the reaction by ^31^P‐NMR (Figure S2F). The complete conversion of this substrate to methyldehydrofosmidomycin is fully consistent with the idea that TMAVP is an intermediate rather than a shunt product.

To determine the stoichiometry of the reaction, we quantified the substrates and products of the reaction after a 6 h incubation using quantitative ^1^H‐NMR to simultaneously measure the consumption/production of 2OG, succinate, TMAEP, TMAVP and methyldehydrofosmidomycin (Table S1). The data show consumption of ≈1.4 2OG (≈1.5 succinate produced) per turnover assuming that one turnover is required to produce TMAVP and two for production of methyldehydrofosmidomycin. The slight excess over the expected value of one, is consistent with an uncoupled reaction that is routinely observed in 2OG‐dependent dioxygenases.^[21]^ Because aqueous formaldehyde is rapidly converted to methylene glycol, whose protons are masked by residual water during ^1^H‐NMR,^[22]^ we used a colorimetric assay to determine the stoichiometry of formaldehyde production in separate reactions, showing 1.14 equivalents of formaldehyde produced for every methyldehydrofosmidomycin.

Due to the relative insensitivity of NMR, we measured O_2_ consumption using a Clark‐type electrode to assess the kinetic properties of DfmD (Figure S3). Based on initial rates during the first minute, the apparent *K*_m_ for TMAEP was 160 μM, while apparent *k*_cat_ is 0.04 s^−1^, leading to a catalytic efficiency of 230 M^−1^ s^−1^. Because the enzyme requires two turnovers to produce the final product, these values must be interpreted with caution. Given the short (60 s) time period used for the assay, the low *k*_cat_ of the enzyme, and our observation of TMAVP as the initial product, we assume that the kinetic values obtained pertain to the desaturation of TMAEP. However, it is possible that some fraction of the TMAVP produced in this reaction remains bound within the active site and is directly oxidized in a second turnover, which significantly complicates the kinetic analysis. Development of more sensitive assays for the phosphonate products will be required to assess this possibility.

**Figure 3:**
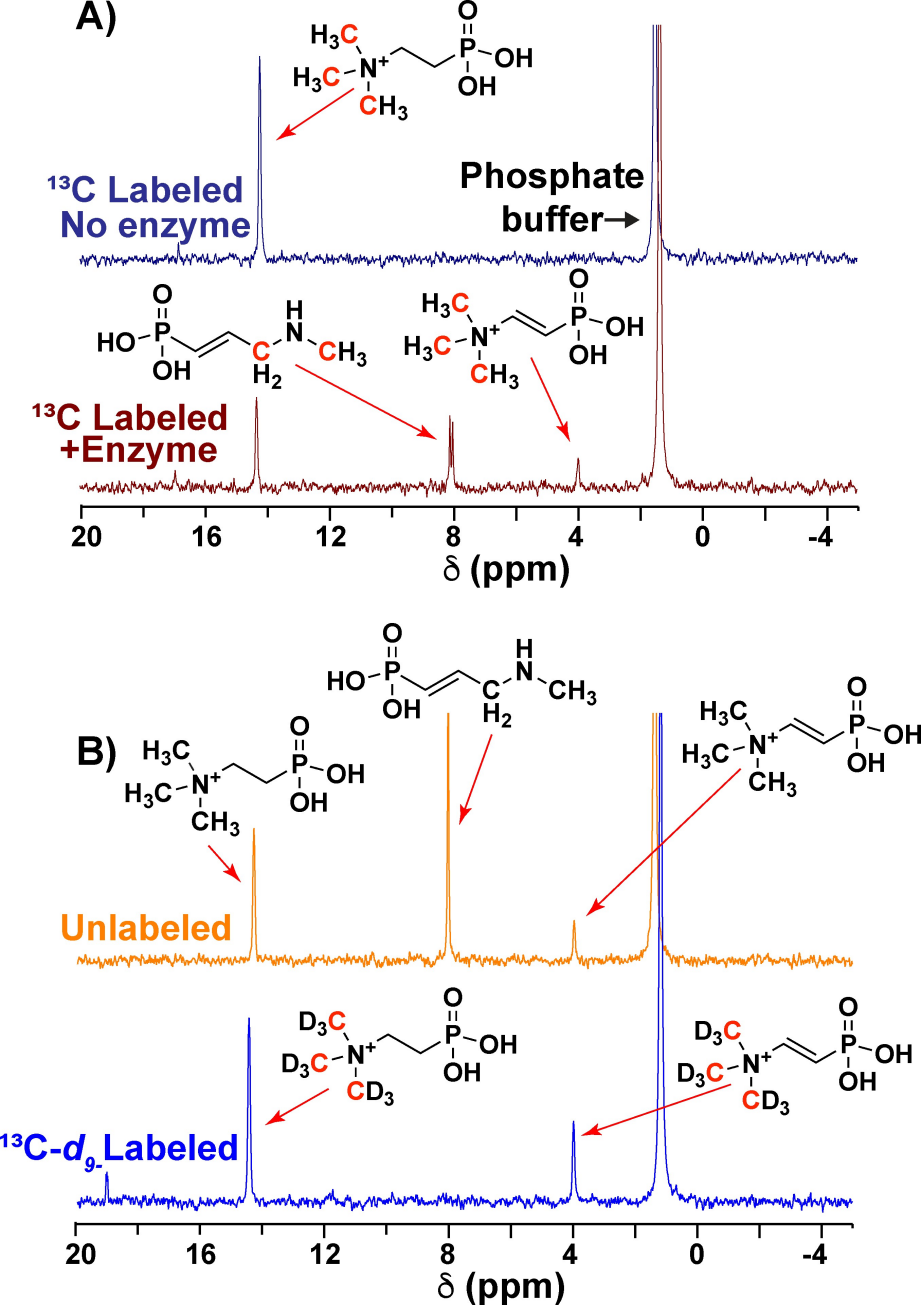
^31^P‐NMR analysis of DfmD activity with isotope labelled substrates. A) Trimethyl‐^13^C_3_‐2‐aminoethylphosphonate was incubated with (red) or without (dark blue) DmfD for 24 h prior to analysis. The compounds giving rise to each peak are also shown, with ^13^C‐labeled atoms shown in red. B) Unlabeled trimethyl‐2‐aminoethylphosphonate (orange) or trimethyl‐^13^C_3_‐d_9_‐2‐aminoethylphosphonate (blue) were incubated with enzyme for 24 h prior to analysis. Representative data from 3 independent experiments is shown. The compounds giving rise to each peak are also shown, with ^13^C‐labeled atoms shown in red. The positions of the labeled carbons in the products were determined by ^13^C‐NMR analysis, Figure S4.

In vivo labeling studies are consistent with the idea that conversion of TMAEP to methyldehydrofosmidomycin involves rearrangement of the molecule resulting in insertion of one of the N‐methyl groups into the carbon skeleton as a methylene moiety.^[11]^ To confirm this, trimethyl‐^13^C_3_‐AEP and trimethyl‐^13^C_3_‐d_9_‐AEP were synthesized and tested as substrates for DfmD (Figure 3). ^31^P‐NMR analysis of in vitro reactions using trimethyl‐^13^C_3_‐AEP revealed products with chemical shifts corresponding to methyldehydrofosmidomycin and TMAVP, with the ^31^P signal in methyldehydrofosmidomycin being split, presumably because the rearrangement of substrate brings the ^13^C close enough to couple with phosphorus. The measured coupling constant of 8 Hz is consistent with this interpretation. ^13^C‐NMR analysis of the reaction is also fully consistent with this interpretation (Figure S4). In addition, ^13^C‐labelled formaldehyde was observed, proving that this product is also derived from one of the labelled *N*‐methyl groups on the substrate. Interestingly, when the ^13^C‐d_9_‐material was incubated with DfmD, TMAVP is the sole observed product (Figures 3 and S4). This is probably due to a very strong isotope effect coupled with a rather sluggish enzyme, which slows the second step of the reaction to a rate that precludes measurable accumulation of the methyldehydrofosmidomycin. In addition, the observed accumulation of TMAVP using the labelled substrate supports the conclusion that desaturation precedes rearrangement.

To provide further insight into substrate binding and catalysis, we tested whether DfmD could process structurally similar phosphonate substrates, including 2‐aminoethylphosphonate, dimethyl‐2‐aminoethylphosphonate, dimethylhydroxy‐2‐aminoethylphosphonate, and trimethyl‐3‐aminopropylphosphonate (Figure S5). Most of these substrates were not processed by DfmD, demonstrating the importance of distance between the phosphorous and the nitrogen, as well as the requirement for tetrasubstituted nitrogen. The absence of activity with dimethyl‐2‐aminoethylphosphonate is also noteworthy, because it suggests that demethylation occurs after rearrangement. Dimethylhydroxy‐2‐aminoethylphosphonate was processed by DfmD, producing vinylphosphonate as the sole (Figure S6). Thus, substitution of a hydroxyl moiety for one of the methyl groups significantly alters the reaction trajectory.

To better understand the position of DfmD within the 2OG‐dioxygenase superfamily, we identified homologous proteins by searching two sequence databases. Surprisingly, despite the hundreds of thousands of 2OG‐dependent dioxygenases that are currently known, close homologs of DfmD were not found. Accordingly, the closest relative within in the Uniprot database of experimentally validated proteins is the γ‐butyrobetaine hydroxylase (BBOX) from *Pseudomonas* sp. AK1,^[23]^ which shares only 29 % identity with DfmD. Within the much larger GenBank‐nr database, the closest homolog shares only 51 % identity with DfmD, with top 100 hits ranging between 30 % and 51 %. Neither BBOX nor TmpA, which uses the same substrate as DfmD and shares 25 % identity, fall within the top 100 GenBank‐nr hits.

**Figure 4:**
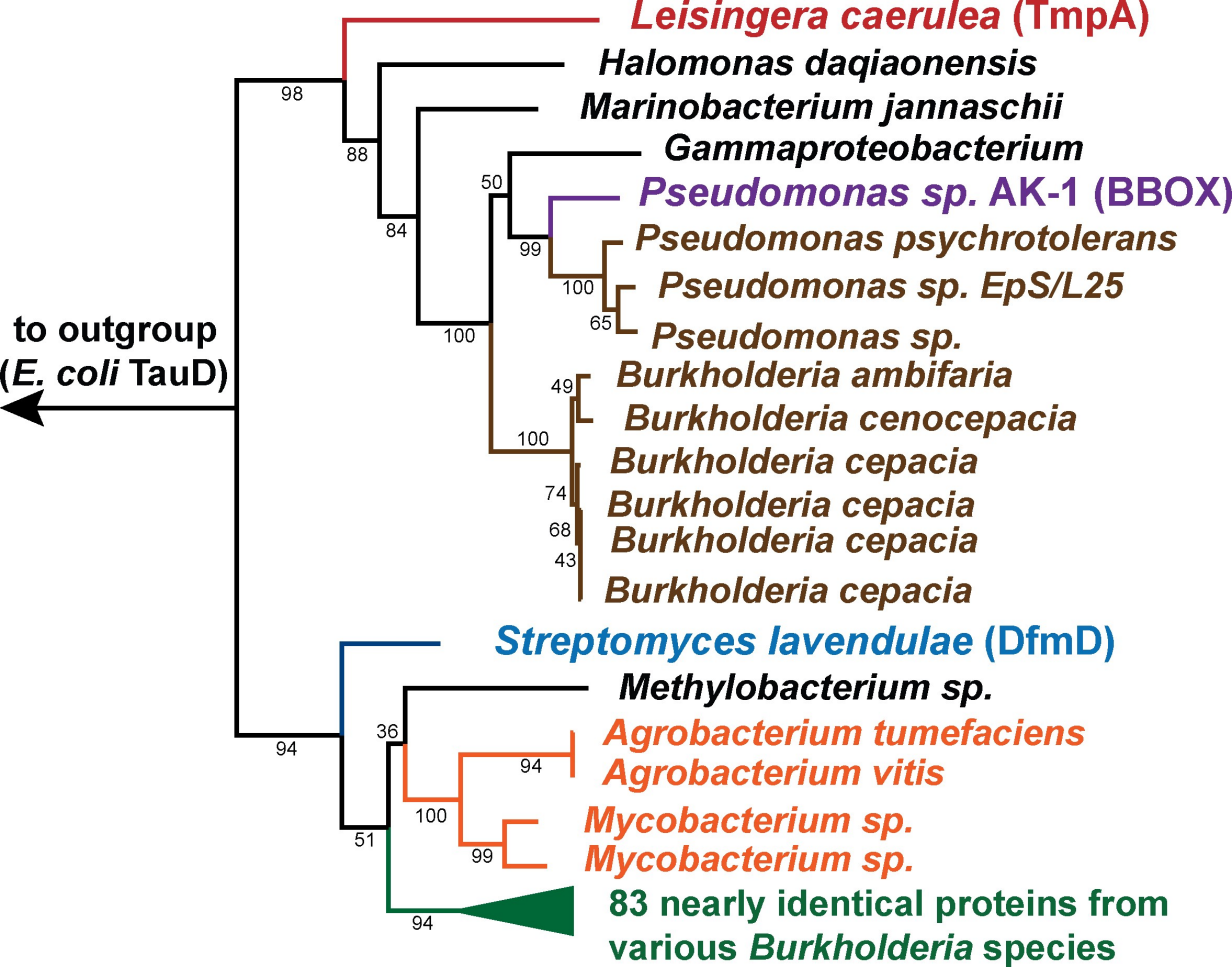
Phylogeny of DfmD homologs. A maximum‐likelihood phylogenetic tree of DfmD and the closest 100 protein homologs is shown. Two proteins with biochemically characterized activities, TmpA and BBOX, were included to provide context, although neither was among the top 100 BLASTP hits. Branches are labelled with the species in which each protein is found. Homologs encoded by genes with highly similar genomic neighborhoods are indicated by common coloring of branches and labels. Graphic depictions of these gene neighborhoods are shown in Figure S6. The genome of *Pseudomonas sp*. AK‐1 has not been sequenced so the genomic context is unknown. The tree was rooted using the *E. coli* TauD protein as an outgroup (not shown). Branches from numerous highly similar sequences from various *Burkholderia* species were collapsed into a single group as shown by the green triangle. Bootstrap support values from 100 replicate trees are shown at each node. The full tree listing the accession numbers of each protein used in this analysis is shown if Figure S7.

A phylogenetic tree of these proteins, using *E. coli* TauD (17 % identity to DfmD) as the outgroup, shows that homologous proteins fall into two large clades, one containing DfmD and the other both TmpA and BBOX (Figure 4). Examination of the gene neighborhoods encoding each homolog suggests that most of the proteins in the BBOX clade are involved in metabolism of carnitine, although the more divergent members, including TmpA, are likely to have different functions (Figure S8). Little can be gleaned from genome neighborhood analysis for members of the DfmD clade because few of the nearby genes have known functions. However, it seems likely that all of these proteins act on *N*‐methylated substrates, given that each of the homologs are encoded within operons that include putative SAM‐dependent *N*‐methyltransferases. Interestingly, the closest (albeit distant) DfmD homologs appear to be involved in the synthesis of phosphonate natural products based on presence of genes encoding PEP mutase and phosphonopyruvate decarboxylase within their respective gene neighborhoods. Moreover, it seems likely that the *Methylobacterium* cluster is responsible for synthesis of a phosphonate related to dehydrofosmidomycin, based on the finding of a nearby gene encoding a putative deoxyxylulose‐Pi reductoisomerase, which acts as self‐resistance gene in organisms that produce Dxr inhibitors.^[13b]^

**Figure 5:**
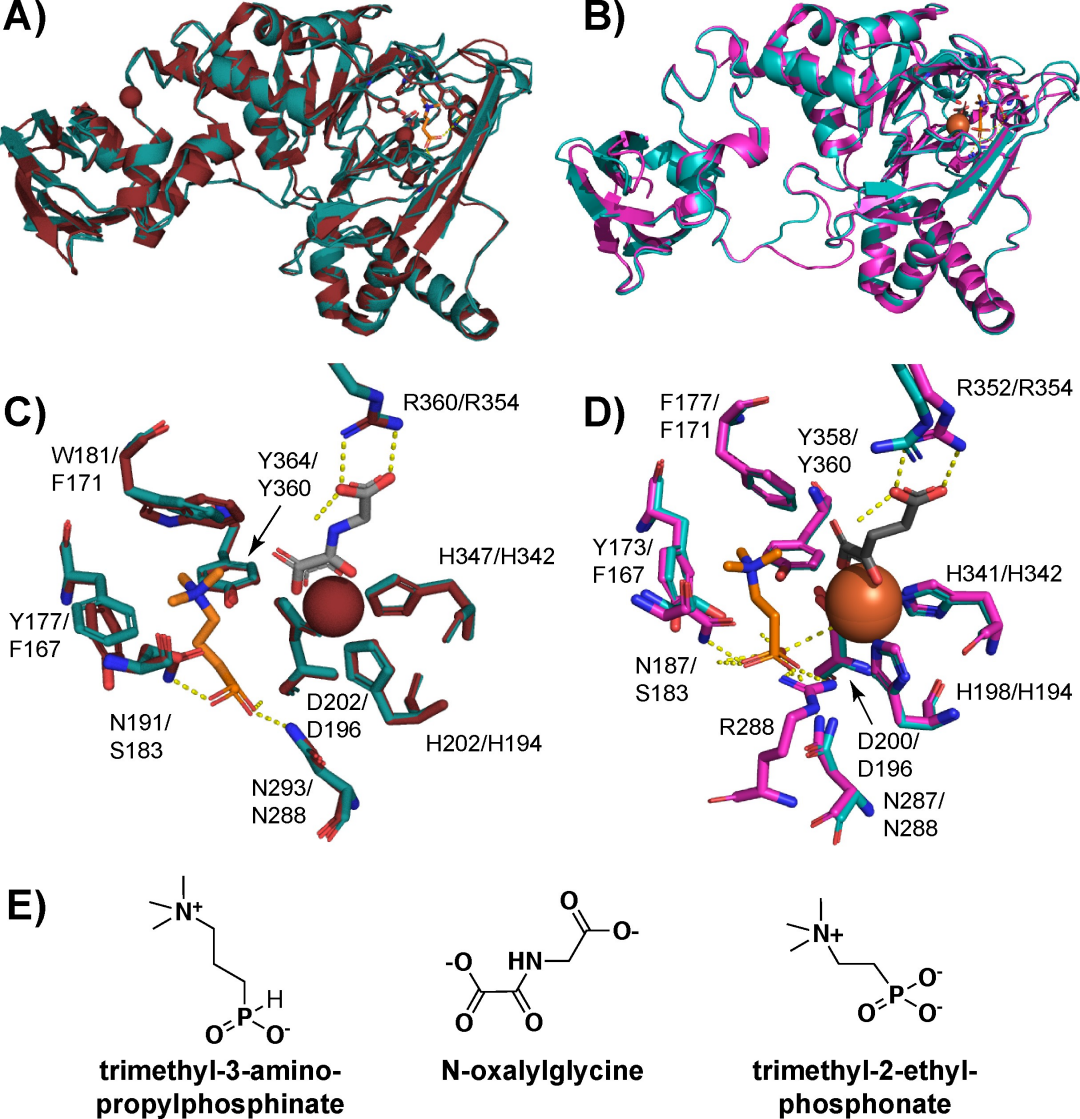
DfmD homology models. A) Homology model of DfmD (teal) aligned with human γ‐butyrobetaine dioxygenase (maroon) with bound 2‐oxoglutarate mimic N‐oxalylglycine (grey), inhibitor trimethyl‐3‐aminopropylphosphinate (orange) and Zn (red sphere, substituting for Fe). B) Homology model of DfmD (teal) aligned with TmpA (pink) with bound substrate trimethyl‐2‐ethylphosphonate (orange), 2‐oxoglutarate (grey) and Fe (orange sphere). C), D) Zoom in on the active sites of each model shown directly below the superimposed structures. E) Structures of the bound substrate and substrate mimics.

Numerous attempts were made to obtain a crystal structure of the DfmD. Unfortunately, although we were able to isolate soluble protein with a variety of affinity tags, none produced diffraction quality crystals with or without removal of the tags and in the presence and absence of substrates. As an alternative, we generated homology models using three different programs: Swiss‐Model,^[24]^ Phyre2,^[25]^ and LOMETS2.^[26]^ We also used AlphaFold to create a de novo model that is not biased by protein homology.^[27]^ All four programs produced very similar structures with RMDS values ranging between 1.6 and 4.6 A relative to the Swiss‐Model, providing confidence in the resulting models (Figure S9). Despite sharing only 28 % sequence identity, the top scoring DfmD model for each of the homology‐based algorithms was derived from the crystal structures of human γ‐butyrobetaine dioxygenase (hBBOX). Significantly, trimethyl‐3‐aminopropylphosphinate, an analog of TMAEP, and N‐oxalyglycine, an analog of 2OG, are present in the hBBOX structure. The fourth highest scoring SwissModel template was TmpA (QMEANDisCo Global=0.66±0.05), whose structure includes both bound TMAEP and 2OG. Thus, the two models make strong predictions regarding the substrate binding and active site residues within DfmD (Figure 5). Accordingly, the three proteins share aromatic amino acids that could stabilize the positively charged trimethyl group through cation‐π interactions.^[28]^ However, they differ in their stabilization of the phosphinate/phosphonate moiety. The human γ‐butyrobetaine dioxygenase has two asparagine residues (N191 and N293) which hydrogen bond with the phosphinate moiety, whereas TmpA has two asparagine residues (N187 and N287) and an arginine residue (R288) that hydrogen bond with the phosphonate. However, DfmD only has one asparagine residue that appears to be within the correct distance to hydrogen bond with the phosphonate residue (N288) with the other asparagine site substituted with a serine (S183). Although no other residues in the model are within bonding range, it is possible that K291, which resides on a nearby flexible loop, could actually play this role. Residues that bind the carboxylate of 2OG (R354) and chelate Fe^+2^ (H194, N196 and H342) are fully conserved in all four models, suggesting that early steps of the reaction pathway involving the O_2_‐dependent generation of succinate and the putative Fe^IV^‐oxo species are essentially identical to other members of the 2OG‐dependent dioxygenase family.

To provide experimental support for the model DfmD structures, we constructed and characterized three DfmD mutants. These include K291A, which we suggest is involved in coordinating the phosphonate moiety of the substrate, and S183A and S185A, which may be involved in the dehydration reaction (see below). All three mutant proteins were well expressed, producing soluble proteins that were correctly folded based on circular dichroism (CD) analysis of the mutant and wild‐type proteins (Figure S10). Despite high enzyme concentrations (10 μM) and long incubations (24 h), neither the K291A nor the S183A mutant showed activity with either TMAEP or TMAVP as substrates, suggesting that these residues are important for activity. In contrast, the S185A mutant was able to convert TMAEP to a mixture of TMAVP and methyldehydrofosmidomycin. The observation of both the intermediate and the product, although at levels much lower than produced by the wild‐type enzyme, shows that this mutant is capable of both half reactions. However, the low levels of methyldehydrofosmidomycin suggest that it is impaired in the rearrangement step.

Based on its enzymatic properties, amino acid sequence and structural modeling, DfmD is clearly a member of the 2OG‐dependent dioxygenase superfamily, albeit one that is significantly different than all known members of this very large and diverse protein family. The most obvious difference is in primary amino acid sequence, as shown by the absence of close homologs in the currently sequenced databases. The low homology of DfmD to proteins with known functions is particularly striking. The unusual rearrangement reaction catalyzed by DfmD is also striking, encompassing steps that involve desaturation of an unactivated carbon‐carbon bond, cleavage of two carbon–nitrogen bonds, oxidation of an *N*‐methyl moiety to free formaldehyde, and formation of a new carbon–carbon bond via insertion of an *N*‐methyl moiety as a methylene group in the carbon skeleton of the final product methyldehydrofosmidomycin. While there is precedent for each of these individual transformations among 2OG‐dependent dioxygenase family members, the combination of all in a single enzyme is, to our knowledge, unprecedented.

Most 2OG‐dependent dioxygenases transform their substrates in a single turnover; however, a few, such as arginine hydroxylase (VioC), clavaminic acid synthase (CAS), carbapenem synthase (CarC) perform multiple turnovers on different substrates, often performing sequential steps in the same biochemical pathway.^[29]^ Our data showing that two turnovers are required for conversion of TMAEP to methyldehydrofosmidomycin, with TMAVP being produced as an intermediate, demonstrate that DfmD belongs to this small subset of 2OG‐dependent dioxygenases. Mechanistic studies of other family members indicate an ordered sequential mechanism in which 2OG binds first, followed by the so‐called “prime” substrate; the “prime” product is then released, followed by succinate, with the latter often being the rate limiting step.^[16b]^ If DfmD follows this pattern, the second turnover, which requires binding of a second 2OG in the site occupied by succinate, could not occur without release of TMAVP. Our observation of free TMAVP during the DfmD reaction is consistent with this idea. However, it must be noted that for some family members succinate release does not always occur in an obligatorily ordered fashion.^[30]^ Indeed, studies of VioC suggest that succinate can be exchanged for 2OG without release of the other product.^[31]^ Therefore, it is possible that some, or even most, of the TMAVP could remain bound during catalysis. As described above, this possibility significantly complicates kinetic analyses of the DfmD.

Our data suggest a unique catalytic mechanism that further differentiates DfmD from other members of the 2OG‐dependent dioxygenase family. Based on sequence conservation and structural modeling, it is likely that early steps of the DfmD reaction up to generation of the Fe^IV^‐oxo intermediate are the same as for other family members. How the reaction proceeds from this intermediate is a matter of speculation, but some insight can be gleaned by comparison to family members that catalyze similar reactions. Two enzymes that catalyze superficially similar rearrangements are BBOX and DAOCS; however, both differ significantly from DfmD in that they catalyze single turnover reactions. Thus, the reaction pathways for these two‐electron oxidations are unlikely to provide good mechanistic models for the DfmD‐catalyzed reactions, despite their utility in development of the homology models presented above. With this in mind it probably more appropriate to consider the DfmD‐catalyzed mechanism of the desaturation and rearrangement separately.

**Figure 6:**
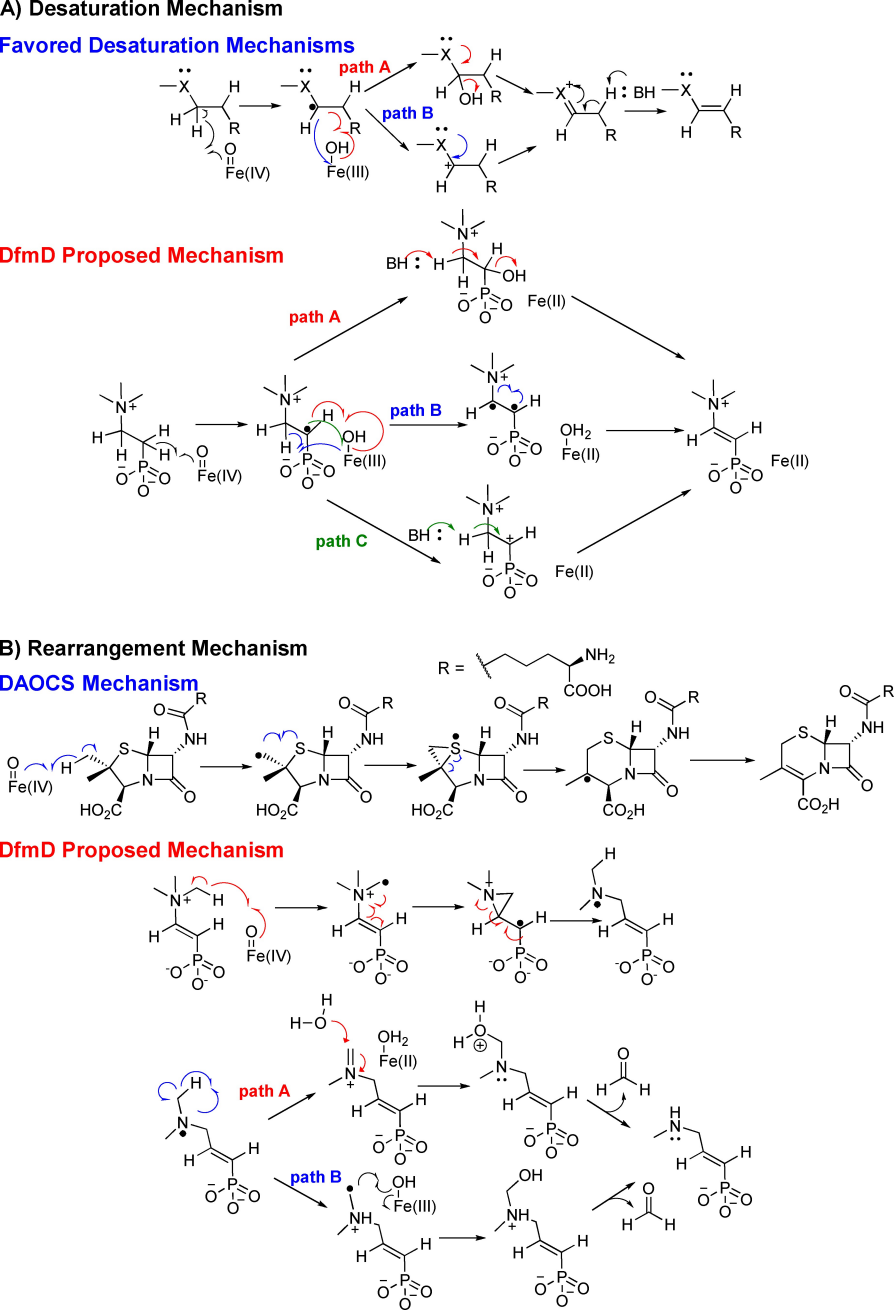
Proposed mechanisms for DfmD‐catalyzed reactions. A) Currently favored desaturation mechanisms are shown at the top. Possible mechansisms for the DfmD‐catalyzed desaturation of TMAEP are shown at the bottom. B) The proposed mechanism of the DAOCS‐catalyzed ring expansion reaction is shown at the top. The proposed DfmD‐catalyzed rearrangement of TMAVP is shown at the bottom.

2OG‐dependent dioxygenase catalyzed desaturations are rather rare, with all known substrates containing a heteroatom with a lone pair of electrons adjacent to the bond that is desaturated.^[16a]^ Indeed, although alternative mechanisms have been proposed, it has been suggested that this heteroatom is required for desaturation and that substrates without such moieties would not be found.^[32]^ Two heteroatom‐dependent mechanisms have been proposed.^[32]^ In the first, the initial radical intermediate is hydroxylated; in the second, it loses an electron to form a carbocation intermediate (Figure 6A). In either case, the heteroatom then uses its lone pair to form a double bond between the heteroatom and adjacent carbon. Subsequent deprotonation of the α‐carbon followed by double bond rearrangement gives the final desaturated product. Neither of these mechanisms are possible for desaturation of TMAEP due to the absence of a lone pair on the tetra‐substituted nitrogen. Moreover, dimethyl‐2‐aminoethyl‐phosphonate, which does have a lone pair on the adjacent nitrogen, is not processed by DfmD. Combined, these data strongly suggest an alternative mechanism of desaturation (Figure 6A). The first step of this mechanism would involve abstraction of a hydrogen atom from the α‐carbon (relative to the phosphonate), similar to the first step in hydroxylation of TMAEP by TmpA. Based on the models presented above, this carbon is positioned 4.1 Å from the Fe^II^, a distance consistent with hydrogen atom abstraction. After initial radical formation, three potential desaturation pathways can be proposed (Figure 6A). In path A, hydroxylation at the α‐position would occur, followed by abstraction of a proton by a base within the active site and subsequent loss of water. (A similar mechanism (not shown) involving hydroxylation at the β‐position could also be envisioned.) In the homology model, two amino acids, deprotonated S183 and S185, stand out as potential bases for the elimination, with their side chain oxygens being 4.1 Å and 4.3 Å away from the β‐carbon, respectively. The loss of activity in the S183A mutant supports the idea that S183 plays this role. Interestingly, the corresponding residue in TmpA (N187) cannot serve this function, providing a potential explanation for different products that are observed. In path B, a second hydrogen atom could be abstracted by the Fe^III^ species resulting in a di‐radical species that would recombine to give the alkene. However, it should be noted that similar proposals have been questioned based on the relatively poor oxidation capacity of the Fe^III^‐OH species.[^16a^, ^32^] In path C, a radical could be transferred to the Fe^III^ to give a di‐cation species that could then undergo proton abstraction. Pathway C seems unlikely due to the improbable formation of two cations within a single molecule. Thus, we favor path A. The production of vinyl phosphonate from dimethylhydroxy‐2‐aminoethylphosphonate is also consistent with this mechanism. In dimethylhydroxy‐2‐aminoethylphosphonate, the hydrogen of the hydroxylamine is much more acidic compared to the hydrogen on the β‐carbon. It is likely that the hydroxyl would be rapidly deprotonated to the dimethylamine oxide by the putative active site base. An elimination reaction in which the *N*‐oxide abstracts a hydrogen from the α‐carbon would then give vinylphosphonate and dimethylamine *N*‐oxide (Figure S6D). Similar reactions have previously been observed with dimethylamine *N*‐oxides.^[33]^ An alternative, non‐oxidative mechanism is also possible, but we believe unlikely (Figure S6D).

While of little help in understanding the desaturation reaction, the DAOCS rearrangement reaction does provide insight into the mechanism of the DfmD‐catalyzed rearrangement of TMAVP to methyldehydrofosmidomycin (Figure 6B). Both enzymes catalyze rearrangements that involve insertion of a methylene group derived from a methyl moiety. In the DAOCS‐catalyzed ring expansion, methylene insertion and desaturation is thought to occur via a thiirane intermediate.^[34]^ We suggest that methylene insertion by DfmD occurs via a similar reaction utilizing an aziridine intermediate. This would make the enzyme quite unique, given that aziridines are very rare in natural products. However, there is some precedent for this idea based on the recent characterization of the 2OG‐dependent dioxygenase TqaL, which produces an aziridine intermediate during the biosynthesis of 2‐aminoisobutyrate, although by a mechanism that is distinct from that we envision for DfmD.^[35]^ The radical SAM enzyme lysine 2,3‐amino mutase is also believed to utilize an aziridine intermediate.^[36]^ The proposed DfmD‐catalyzed rearrangement would begin by hydrogen atom transfer from one of the trimethyl groups, which is positioned 4.6 Å above the Fe^II^, in the homology model. Support for this initial reaction is provided by the strong isotope effect of deuterated *N*‐methyl groups, which we showed prevents the rearrangement, but not the desaturation. The resulting methyl centered radical would then react with the alkene to form an aziridine intermediate that is expected to be quite unstable due to the tetrasubstituted nitrogen. As a result, we would expect immediate opening of the aziridine ring via beta scission of the C−N bond, which is energetically more favorable than cleavage of the C−C bond.^[37]^ After oxidation, two pathways for resolution of the unstable nitrogen centered radical can be envisioned, both of which produce formaldehyde and methyldehydrofosmidomycin (Figure 6B).

## Conclusion

DfmD is a highly unusual 2OG‐dependent dioxygenase, whose study provides new mechanistic insight to the catalytic mechanisms of this enzyme superfamily. The absence of a lone pair‐bearing heteroatom in TMAEP calls for a reassessment of the currently favored desaturation mechanisms, while the predicted formation of an aziridine intermediate in the rearrangement reaction suggests new chemistry for this enzyme class. Consistent with the unusual biochemistry of the enzyme, phylogenetic analysis of DfmD shows that it is only distantly related to other family members. Thus, while the closest characterized relatives, TmpA and BBOX catalyze superficially similar reaction, they clearly diverged from a common ancestor long ago and have been shaped by different evolutionary pressures. Despite these differences, the structural models presented here make strong predictions regarding amino acid residues that enable the divergent chemistry displayed by the respective enzymes, which are supported by characterization of mutants lacking these residues.

## Conflict of interest

William W. Metcalf is a co‐owner of the natural products discovery company MicroMGX.

1

## Supporting information

As a service to our authors and readers, this journal provides supporting information supplied by the authors. Such materials are peer reviewed and may be re‐organized for online delivery, but are not copy‐edited or typeset. Technical support issues arising from supporting information (other than missing files) should be addressed to the authors.

Supporting Information

## Data Availability

The data that support the findings of this study are available from the corresponding author upon reasonable request.
