# Supplementary material for: An Unusual Oxidative Rearrangement Catalyzed by a Divergent Member of the 2‐Oxoglutarate‐Dependent Dioxygenase Superfamily during Biosynthesis of Dehydrofosmidomycin
Source: Angew Chem Int Ed Engl. 2022 Jun 7;61(30):e202206173. doi: 10.1002/anie.202206173 (PMC9296572; doi:10.1002/anie.202206173)
Supplement: Supplementary file 1 — Supporting Information [file ANIE-61-0-s001.pdf]

Angewandte Chemie
Eine Zeitschrift der Gesellschaft Deutscher Chemiker

Supporting Information

An Unusual Oxidative Rearrangement Catalyzed by a Divergent Member of the 2-Oxoglutarate-Dependent Dioxygenase Superfamily during Biosynthesis of Dehydrofosmidomycin

E. I. Parkinson, H. G. Lakkis, A. A. Alwali, M. E. M. Metcalf, R. Modi, W. W. Metcalf**

Supplemental Information Table of Contents

Experimental Section……………………………………………………….…………...……….3-14

Supplemental Figure 1…………………………………………………………………………..15

Supplemental Figure 2………………………………………………………………...………16

Supplemental Figure 3…………………………………………………………...…………17

Supplemental Figure 4…………………………………………………………...…………18

Supplemental Figure 5…………………………………………………….…………………19

Supplemental Figure 6…………………………………………………………………………..20

Supplemental Figure 7……………………………………………..………………………21

Supplemental Figure 8……………………………………………..………………………22

Supplemental Figure 9……………………………………………..………………………23

Supplemental Figure 10………………………………………..…………………..……24

Supplemental Table 1……………………………………………………………………...…25

Supplemental References……………………………………...…………………..……….25

Genbank-formatted sequence of the dehydrofosmidomycin biosynthetic gene cluster......….26-35

NMRs……………………………………...…………………………………......….36-93

Experimental Section

Chemicals: General chemical reagents were purchased from Sigma Aldrich. Diethyl allylphosphonate (CAS: 5954-65-4) was purchased from Alfa Chemistry. Solvents were dried by passage through columns packed with activated alumina.

Nuclear Magnetic Resonance (NMR): ^{1}H-NMR, ^{13}C-NMR, and ^{31}P-NMR were recorded on either an Agilent DD2 600 MHz spectrometer (600 MHz for ^{1}H, 150 MHz for ^{13}C and 243 MHz for ^{31}P) or a Bruker 500 MHz spectrometer equipped with a CryoProbe (500 MHz for ^{1}H, 126 MHz for ^{13}C and 203 MHz for ^{31}P). Spectra generated from a solution of $CDCl_3$ were referenced to residual chloroform (^{1}H: δ 7.26 ppm, ^{13}C: δ 77.16 ppm). Spectra generated from a solution of D_2O were referenced to residual water (^{1}H: δ 4.79 ppm).

Liquid Chromatography High Resolution Mass Spectrometry (LC-HRMS):
HRMS for synthetic molecules was performed by the UIUC Mass Spectrometry Laboratory. Unless otherwise noted, all LC-HRMS/MS experiments on enzyme reactions were performed at the UIUC Metabolomics Center using the following protocol. Briefly, samples were re-suspended in 75% ACN. Samples were vortexed and sonicated before filtering through a 0.2 µm syringe filter. For high resolution LC/MS, the samples were analyzed by using the Q-Exactive MS system (Thermo. Bremen, Germany) in the Metabolomics Laboratory of Roy J. Carver Biotechnology Center, University of Illinois at Urbana-Champaign. Software Xcalibur 4.1.31.9 was used for data acquisition and analysis. The Dionex Ultimate 3000 series HPLC system (Thermo, Germering, Germany) used includes a degasser, an autosampler, and a binary pump. The LC separation was performed on a 250 X 4.6 mm inner diameter, 4-uM particle size Hydro-RP column (Phenomenex) with mobile phase A (water) and mobile phase B (acetonitrile). The flow rate was 0.5 mL/min. The linear gradient was as follows: 0 to 1 min, 0% B, 1 to 5 min, gradient to 10% B, 5 to 35 min, gradient to 100% B, 35 to 45 min, 100% B, 46 min, to 0% B, 55 min, 0% B. The autosampler was set to 10°C. The injection volume was 10 µL. Mass spectra were acquired under both positive (sheath gas flow rate, 52; aux gas flow rate: 13.5; sweep gas flow rate, 3.5; spray voltage, 3.5 kV; capillary temp, 268 °C; Aux gas heater temp, 430 °C) and negative electrospray ionization (sheath gas flow rate, 52; aux gas flow rate: 13.5; sweep gas flow rate, 3.5; spray voltage, -2.5 kV; capillary temp, 268 °C; Aux gas heater temp, 430 °C). The full scan mass spectrum resolution was set to 70,000 with the scan range of m/z 230 ~ m/z 1,600, and the AGC target was 1E6 with a maximum injection time of 200 ms. For MS/MS scan, the mass spectrum resolution was set to 17,500. AGC target was 5E4 with a maximum injection time of 50 ms. Loop count was 4. Isolation window was 1.0 m/z with NCE of 30 and 40 eV.

DfmD phylogeny and genome neighborhood analysis.

DfmD was used as the query sequence for a BlastP search of the NCBI Genbank non-redundant protein database on October 20, 2021.[1] The top 100 hits were downloaded, along with the sequences of *E. coli* TauD (accession #P37610) and *Leisingera caerulea* TmpA (accession # 6NPB_A) and γ-butyrobetaine hydroxylase (BBOX) from Pseudomonas sp. AK-1 (accession #P80193). The proteins were aligned using MUSCLE with default parameters.[2] The resulting alignment was used as the input for the maximum-likelihood tree generating program RAxML

using rapid bootstrapping to provide statistical support and to identify the best scoring tree.[3] Gene neighborhoods surrounding each homolog were manually retrieved and the putative functions assigned based on the current gene annotations and conserved domain searches at NCBI. The genome sequence of the dehydrofosmidomycin-producing organism, *Streptomyces lavendulae* Fujisawa 8006 (Genbank accession # GCA_000715625.1) is not annotated at the protein level; thus, we have provided a fully annotated sequence, including ORF translations used for all bioinformatics results in this supplemental section (see below).

Purification of DfmD and mutant derviatives. DfmD and mutant derivatives were purified as described.[4] Site-directed mutants were constructed by replacement of an internal AgeI fragment from the DfmD overexpression plasmid pEIP001 with synthetic DNA (IDT) carrying the desired mutations. These fragments were inserted into AgeI-cut pEIP001 via HiFi Assembly (New England Biolabs). All plasmids were sequence verified at Roy J. Carver Biotechnology Center, University of Illinois at Urbana-Champaign. Correct folding of the purified mutant derivatives was assessed by Circular Dichroism after using BeStSel for analysis.[6]

DfmD activity assays by NMR and LC-HRMS/MS. NMR experiments were performed on an Agilent 600 MHz spectrometer equipped with a OneNMR probe. A typical assay (500 µL) contained 5 µM enzyme, 200 µM $Fe(II)(NH_4)_2(SO_4)_2$, 10 mM 2-oxoglutarate, 1 mM L-ascorbate, and 5 mM substrate in phosphate-buffered saline (PBS, 137 mM NaCl, 2.7 mM KCl, 10 mM Na_2HPO_4, and 1.8 mM KH_2PO_4, pH 7.4). Samples were typically performed in 15 mL conical tubes left open on their side for maximum oxygen exchange. Assays were performed at room temperature for 6 h unless otherwise noted. The reaction was then treated with Chelex (~100 µL) for 5 min to remove iron, and the protein was removed using a 30 kDa cutoff Amicon Ultra-0.5 mL centrifugal filter (EMD Millipore). D_2O (100 µL) was added prior to analysis by ^{31}P and ^{1}H NMR. Any spiking with known standards was performed after the reaction. Spectra were analyzed using MestReNova version 10.0.2. An aliquot of each samples (5 µL) was diluted into dH_2O (45 µL) and ACN (150 µL), mixed, and filtered through 0.2 um syringe filters prior to analysis via LC-HRMS/MS.

Quantitative NMR (qNMR) analysis for stoichiometry determination

Reactions were performed as described in “DfmD activity assays by NMR and LC-HRMS/MS” with the following modifications: To prevent evaporation, experiments were performed in closed 2 mL Eppendorf tubes laid on their side for maximum oxygen exchange. To ensure proper quantification, neither the chelex treatment nor the amicon centrifugal filters were utilized. Instead, after the 6 h reaction, all samples were placed on ice for 5 minutes then spun at 14,000 X g for 5 minutes. 100 µL of D_2O were then added for a total volume of 600 µL. Immediately prior to analysis, a 125 mM stock of potassium hydrogen phthalate in D_2O was prepared. For each sample, 14 µL of the potassium hydrogen phthalate stock (final concentration 2.87 mM) was added. Samples were kept at -20 until NMR analysis. Prior to analysis, samples were spun at at 14,000 X g for 5 minutes before being transferred to NMR tubes.

^{1}H-NMR were recorded on a Bruker DRX500 with a 5mm BBFO Z-gradient ATM probe. T1 analysis was performed for the complete reaction mixture. The longest T1 was the potassium hydrogen phthalate (3.1 s). The interscan delay was then set to 7X the longest T1 (21.7 s).

Specifically, the acquisition time was set to 2.7 s and the relaxation delay was set to 19.0s. 32 scans were performed. Each experiment was performed three independent times.

Colorimetric formaldehyde quantification assay

Reactions were performed as described in "DfmD activity assays by NMR and LC-HRMS/MS". The colorimetric formaldehyde assay was performed as previously described.[5] Briefly, 15 g of ammonium acetate was mixed with 0.2 mL acetylacetone and 0.3 mL glacial acetic acid and diluted to 100 mL with MilliQ water to yield the acetylacetone solution. 100 µL of acetylacetone solution was added to either freshly made formaldehyde standards or to enzyme reactions. The samples were heated to 60 °C for 10 min and their absorbance at 345 nm was read using a Tecan Platereader. The calibration curve consisted of 3 technical replicates of concentrations varying from 0.5 to 20 µg/mL (0.016 to 0.63 mM). Any samples that were not within the linear range of the controls were diluted to within this range and re-examined.

Kinetic Characterization of DfmD

DfmD (144 µM) was anaerobically reconstituted with 7-fold $(NH_4)_2Fe(II)(SO_4)_2$ (1 mM) in PBS pH 7.4 and the protein solution was allowed to incubate on ice for at least 10 min. The activity of the protein solution was then assayed by monitoring the rate of O_2 consumption in air-saturated buffer (280 µM O_2, PBS pH 7.4) at 20 °C in the presence of DfmD (6.5 µM DfmD and 46 µM Fe (II)) and varying concentrations of trimethyl-2-AEP (0, 25, 50, 75, 100, 150, 250, 500 and 1000 µM). Michaelis-Menten parameters were determined from 3 independent experiments. OriginPro was used for the analysis of the data.

Chemistry

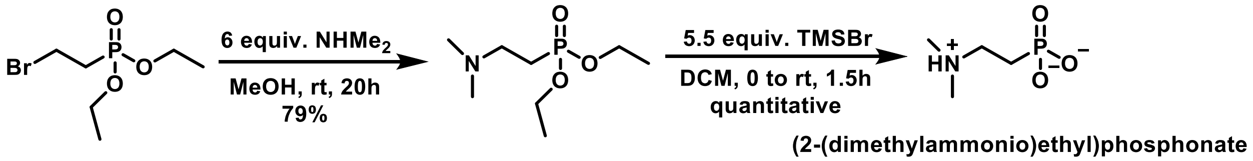

diethyl (2-bromoethyl) phosphonate **diethyl (2-(dimethylamino)ethyl)phosphonate (S1)** **(2-(dimethylammonio)ethyl)phosphonate (dimethyl-AEP)**

diethyl (2-(dimethylamino)ethyl)phosphonate (S1). To an oven dried 20 mL vial with stirbar was added diethyl (2-bromoethyl) phosphonate (243 mg, 0.99 mmol, Sigma-Aldrich). The vial was evacuated and refilled with nitrogen three times, and dimethylamine in methanol was added (3 mL of a 2.0 M solution, 6 mmol, Sigma-Aldrich). The reaction was allowed to stir at room

temperature for 20 h. The reaction was then dried via rotary evaporation to yield 163 mg (0.78 mmol, 79% yield) clean **S1**.
1H NMR (500 MHz, CDCl3) δ 4.10 (m, 4H), 2.59 (q, 2H, J = 8 Hz), 2.25 (s, 6H), 1.96 (m, 2H), 1.32 (td, 6H, J = 7 Hz, 0.9 Hz).
31P NMR (203 MHz, CDCl3) δ 30.34.
13C NMR (126 MHz, CDCl3) δ 61.7 (d, J = 6.4 Hz), 52.8, 44.9, 25.0 (d, J = 139 Hz), 16.6 (d, J = 6.0 Hz).
HRMS (ESI) calcd for $C_8H_{21}NO_3P$ (M+H)+: 210.1259, found: 210.1259, Δppm: 0.0.

(2-(dimethylammonio)ethyl)phosphonate (dimethyl-AEP). To an oven dried 20 mL vial with stirbar was added **S1** (86 mg, 0.41 mmol). The vial was evacuated and refilled with nitrogen three times, and dry DCM (1.5 mL) was added. The reaction was cooled to 0 °C, and bromotrimethylsilane (300 µL, 348 mg, 2.3 mmol, Sigma-Aldrich) was added with stirring. The reaction was allowed to gradually warm to room temperature and was stirred for 1.5 h. The reaction was then dried via rotary evaporation, and the resulting oil was dissolved in dH_2O (3 mL) and allowed to stir for 1h at room temperature. The solution was then dried via rotary evaporation to yield 67 mg (0.41 mmol, quantitative yield) clean DM-2AEP.
1H NMR (600 MHz, d2o) δ 3.22 (m, 2H), 2.78 (s, 6H), 1.95 (m, 2H).
31P NMR (243 MHz, d2o) δ 18.57.
13C NMR (151 MHz, d2o) δ 53.4, 42.4, 23.9 (d, J = 132 Hz).
HRMS (ESI) calcd for $C_4H_{13}NO_3P$ (M+H)+: 154.0633, found: 154.0630, Δppm: -1.9.

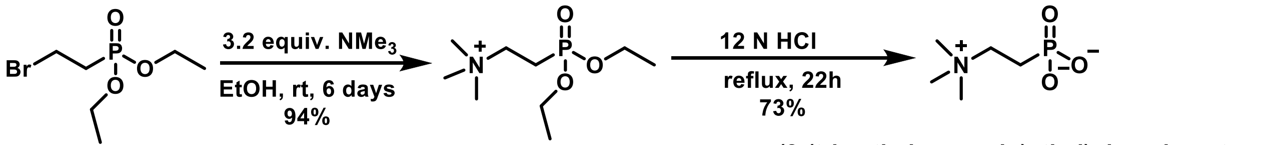

diethyl (2-bromoethyl) phosphonate | **2-(diethoxyphosphoryl)-*N*,*N*,*N*-trimethylethan-1-aminium (S2)** | **(2-(trimethylammonio)ethyl)phosphonate (trimethylAEP)**

2-(diethoxyphosphoryl)-N,N,N-trimethylethan-1-aminium (S2). **S2** was synthesized using a similar procedure to one previously published.[7] Diethyl 2-bromoethylphosphonate (163 mg, 0.67 mmol, Sigma-Aldrich) was added to an oven dried 20 mL vial with stirbar. The vial was evacuated and refilled three times with nitrogen. To this, trimethylamine in ethanol (0.5 mL of a 4.2 M solution, 2.1 mmol trimethylamine, 3.2 equiv., Sigma-Aldrich) was added. The slurry was stirred for 6 days at room temperature. The reaction was then dried via rotary evaporation. The solid was then dissolved in 1 mL of dH_2O and loaded onto a 15.5 g C18Aq. The following gradient was then run using a Combiflash Rf+ and a flow rate of 30 mL/min (Teledyne Isco): 1 min at 100% solvent A (dH2O), linear gradient to 50% solvent B (ACN), 2 min at 100% B, and 1 min at 80%B. Fractions were screened via TLC using $KMnO_4$ stain. When run in EtOAc, Rf of starting material = 0.34. The product does not move from baseline. Fractions containing product were combined and dried via rotary evaporation to yield 191 mg of product as the bromide salt (0.63 mmol, 94% yield). There was a small impurity, but this was taken on through the next step.
1H NMR (600 MHz, D_2O) δ 4.24 (quintet, 4H, J = 7 Hz), 3.66 (m, 2H), 3.19 (s, 9H), 2.56 (m, 2H), 1.38 (t, 6H, J = 7 Hz).

31P NMR (243 MHz, D_2O) δ 27.18.
13C NMR (151 MHz, D_2O) δ 64.1 (d, *J* = 7 Hz), 60.2 (d, *J* = 2 Hz), 52.7 (t, *J* = 4 Hz), 20.1 (d, *J* = 140 Hz), 15.5 (d, *J* = 6 Hz).
HRMS (ESI) calcd for $C_9H_{23}NO_3P$ (M+H)+: 224.1416 , found: 224.1417, Δppm: 0.4.

N,N,N-trimethyl-2-phosphonoethan-1-aminium (trimethyl-AEP). To a 50 mL round bottom flask with stirbar was added **S2** (182 mg, 0.60 mmol) and concentrated HCl (6 mL). The solution was refluxed for 22 h. The solution was dried via rotary evaporation. The product was then recrystallized from hot ethanol. This gave TM-2AEP as white crystals (73 mg, 0.44 mmol, 73% yield).
1H NMR (600 MHz, d2o) δ 3.56 (m, 2H), 3.16 (s, 9H), 2.20 (m, 2H).
31P NMR (243 MHz, D_2O) δ 18.09.
13C NMR (151 MHz, d2o) δ 62.4 (d, *J* = 3 Hz), 52.5 (t, *J* = 4 Hz), 22.9 (d, *J* = 130 Hz).
HRMS (ESI) calcd for $C_5H_{15}NO_3P$ (M+H)+: 168.0790, found: 168.0790, Δppm: 0.0.

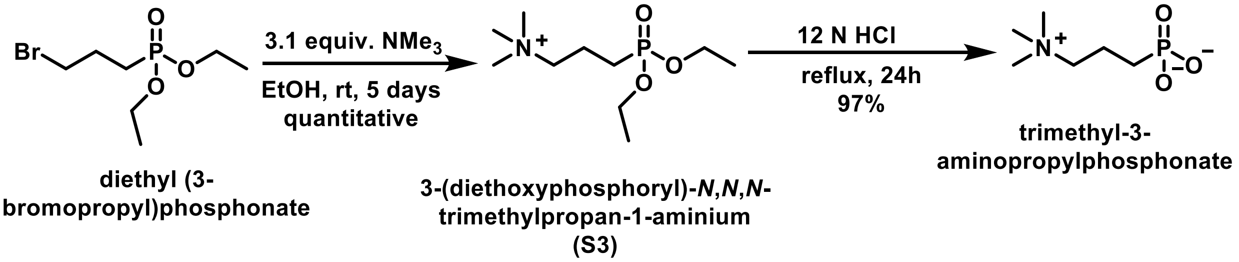

3-(diethoxyphosphoryl)-N,N,N-trimethylpropan-1-aminium (S3). **S3** was synthesized using a similar procedure to one previously published.[7] Diethyl (3-bromopropyl)phosphonate (173 mg, 0.67 mmol, Sigma-Aldrich) was added to an oven dried 20 mL vial with stirbar. The vial was evacuated and refilled three times with nitrogen. To this, trimethylamine in ethanol (0.5 mL of a 4.2 M solution, 2.1 mmol trimethylamine, 3.2 equiv., Sigma-Aldrich) was added. The slurry was stirred for 5 days at room temperature. The reaction was then dried via rotary evaporation to yield 215 mg of product as the bromide salt (0.67 mmol, quantitative yield).
1H NMR (500 MHz, D2O) δ 4.18 (m, 4H), 3.43 (m, 2H), 3.16 (s, 9H), 2.10 (m, 2H), 1.99 (m, 2H), 1.36 (t, 6H, *J* = 7 Hz).
31P NMR (203 MHz, D2O) δ 33.04.
13C NMR (126 MHz, D2O) δ 65.9 (dt, *J* = 21 Hz, 3 Hz), 63.5 (d, *J* = 6.7 Hz), 52.9 (t, *J* = 4.0 Hz), 21.3 (d, *J* = 143 Hz), 16.1 (d, *J* = 4.1 Hz), 15.5 (d, *J* = 5.8 Hz).
HRMS (ESI) calcd for $C_{10}H_{25}NO_3P$ (M+H)+: 238.1572, found: 238.1573, Δppm: 0.4.

(3-(trimethylammonio)propyl)phosphonate (trimethyl-3-aminopropylphosphonate). To a 50 mL round bottom flask with stirbar was added **S3** (210 mg, 0.63 mmol) and concentrated HCl (6 mL). The solution was refluxed for 24 h. The solution was dried via rotary evaporation. The product was then recrystallized from hot ethanol. This gave TM-3APP as white crystals (111 mg, 0.61 mmol, 97% yield).
1H NMR (600 MHz, d2o) δ 3.42 (m, 2H), 3.15 (s, 9H), 2.08 (m, 2H), 1.77 (m, 2H).
31P NMR (243 MHz, d2o) δ 26.20.
13C NMR (151 MHz, d2o) δ 66.4 (dt, *J* = 20 Hz, 3.0 Hz), 52.8 (t, *J* = 4.0 Hz), 23.9 (d, *J* = 137 Hz), 16.9 (d, *J* = 3.5 Hz).
HRMS (ESI) calcd for $C_6H_{17}NO_3P$ (M+H)+: 182.0946, found: 182.0951, Δppm: 2.7.

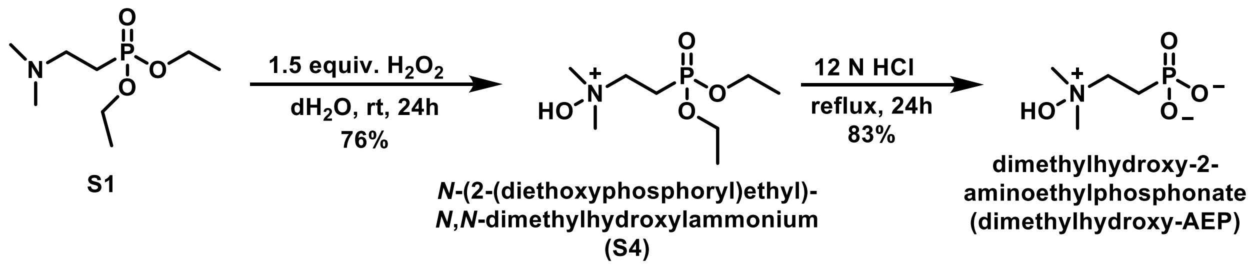

N-(2-(diethoxyphosphoryl)ethyl)-N,N-dimethylhydroxylammonium (S4). **S1** was synthesized as described above. To a 4 mL vial with stirbar was added **S1** (146 mg, 0.70 mmol). The vial was evacuated and refilled three times with nitrogen. dH_2O (900 µL) and H_2O_2 (100 uL of 9.8 M H_2O_2 in dH_2O, 1.5 equiv.) were added to the vial and allowed to stir for 24 h at room temperature. A small spatula full of catalase (Bovine Liver, C9322 from Sigma-Aldrich) was added, and the reaction was allowed to stir until bubbling ceased (~1 h). The reaction was dried via rotary evaporation. The solid was then dissolved in 1 mL of dH_2O and loaded onto a 15.5 g C18Aq. The following gradient was then run using a Combiflash Rf+ and a flow rate of 30 mL/min (Teledyne Isco): 1 min at 100% solvent A (dH2O), linear gradient to 100% solvent B (ACN) over 19 min, 2 min at 100% B, and 1 min at 80%B. Fractions were screened via TLC using $KMnO_4$ stain. Fractions containing product were combined and dried via rotary evaporation to yield 158 mg of product (0.70 mmol, quantitative yield). There was a slight impurity, but this was taken through the next step.
1H NMR (600 MHz, d2o) δ 4.17 (m, 4H), 3.56 (m, 2H), 3.22 (s, 6H), 2.49 (m, 2H), 1.33 (t, 6H, *J* = 7.1 Hz).
31P NMR (243 MHz, d2o) δ 29.17.
13C NMR (151 MHz, d2o) δ 63.8 (d, *J* = 6.6 Hz), 63.5 (d, *J* = 2.1 Hz), 57.2, 20.1 (d, *J* = 140 Hz), 15.5 (d, *J* = 5.8 Hz).
HRMS (ESI) calcd for $C_8H_{21}NO_4P$ (M+): 226.1208, found: 226.1212, Δppm: 1.8.

(2-(hydroxydimethylammonio)ethyl)phosphonate (dimethylhydroxy-AEP). To a 50 mL round bottom flask with stirbar was added **S4** (120 mg, 0.53 mmol) and 12 N HCl (6 mL). The reaction was refluxed for 24 h and then dried via rotary evaporation to yield 75 mg (0.44 mmol, 83% yield) DMOH-2AEP.
1H NMR (600 MHz, d2o) δ 3.80 (m, 2H), 3.47 (s, 6H), 2.20 (m, 2H).
31P NMR (243 MHz, d2o) δ 16.97.
13C NMR (151 MHz, d2o) δ 65.6, 55.5, 23.2 (d, *J* = 129 Hz).
HRMS (ESI) calcd for $C_4H_{13}NO_4P$ (M+): 170.0582, found: 170.0583, Δppm: 0.6.

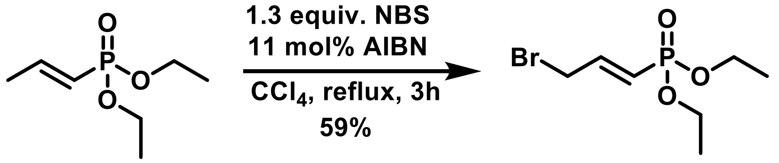

diethyl (*E*)-prop-1-en-1-ylphosphonate **diethyl (*E*)-(3-bromoprop-1-en-1-yl)phosphonate (S5)**

Diethyl (E)-(3-bromoprop-1-en-1-yl)phosphonate (S5). To an oven dried 20 mL vial with stirbar was added diethyl (E)-prop-1-en-1-ylphosphonate (497 mg, 2.79 mmol), *N*-bromosuccinimide (660 mg, 3.71 mmol), and AIBN (50 mg, 0.30 mmol). The vial was evacuated and refilled with nitrogen three times and CCl_4 (5 mL) was added. Reaction was then refluxed (76°C) for 3 h. The solution was cooled to 0°C and the precipitate was filtered off. The crude was purified by silica gel chromatography (9:1 hexanes:ethyl acetate to 1:1 hexanes:ethyl acetate) to yield the product (423 mg, 1.65 mmol, 59% yield). Rf = 0.24 in ethyl acetate, stained with $KMnO_4$ stain.

1H NMR ($CDCl_3$, 600 MHz): δ 6.81 (m, 1H), 5.93 (dt, 1H, *J* =17, 1.2 Hz), 4.10 (dq, 4H, *J* = 7, 1.2 Hz), 3.99 (dt, 2H, *J* = 7, 1.2 Hz), 1.33 (t, 6H, *J* = 7 Hz).
31P NMR ($CDCl_3$, 243 MHz): δ 16.04
13C NMR ($CDCl_3$, 150 MHz): δ 145.8 (d, *J* = 6 Hz), 121.5 (d, *J* = 188 Hz), 62.2 (d, *J* = 6 Hz), 30.7 (d, *J* = 27 Hz), 16.5 (d, *J* = 6 Hz)
HRMS (ESI) calcd for $C_7H_{15}O_3BrP$ (M+H)+: 256.9942, found: 256.9940, Δppm: -0.8.

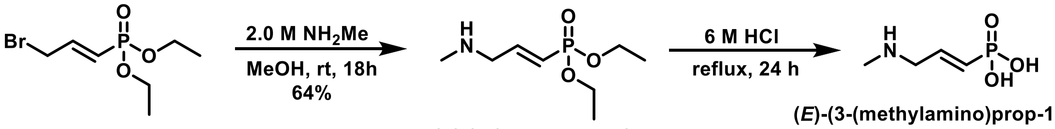

diethyl (*E*)-(3-bromoprop-1-en-1-yl)phosphonate (S5) **diethyl (*E*)-(3-(methylamino)prop-1-en-1-yl)phosphonate (S6)** **(*E*)-(3-(methylamino)prop-1-en-1-yl)phosphonic acid (methyldehydroFOM)**

Diethyl (E)-(3-(methylamino)prop-1-en-1-yl)phosphonate (S6). To an oven dried 20 mL flask with stirbar was added **S6** (37 mg, 0.14 mmol). The vial was evacuated and refilled three times with nitrogen. To it was added methylamine (1.5 mL of 2.0 M in methanol, 3.0 mmol, 21 equiv.), and the reaction was stirred at room temperature for 18 h. The reaction was dried via rotary evaporation. The solid was then dissolved in 1 mL of dH_2O and loaded onto a 15.5 g C18Aq column. The following gradient was then run using a Combiflash Rf+ and a flow rate of 30 mL/min (Teledyne Isco): 1 min at 100% solvent A (dH2O), linear gradient to 50% solvent B (ACN) over 16 min, 2 min at 100% B, and 1 min at 80%B. Fractions were screened via TLC

using $KMnO_4$ stain. Fractions containing product were combined and dried via rotary evaporation to yield 19 mg of product (0.09 mmol, 65% yield).
1H NMR (600 MHz, d2o) δ 6.78 (m, 1H), 6.29 (t, 1H, *J* = 18.9 Hz), 4.19 (m, 4H), 3.92 (d, 2H, *J* = 6.0 Hz), 2.79 (s, 3H), 1.36 (t, 6H, *J* = 7.1 Hz).
31P NMR (243 MHz, d2o) δ 17.83.
13C NMR (151 MHz, d2o) δ 142.1 (d, *J* = 5.6 Hz), 123.0 (d, *J* = 186 Hz), 64.0 (d, *J* = 5.9 Hz), 49.9 (d, *J* = 25.9 Hz), 32.4, 15.4 (d, *J* = 5.9 Hz).
HRMS (ESI) calcd for $C_8H_{19}NO_3P$ (M+H)+: 208.1103, found: 208.1101, Δppm: -1.0.

(E)-(3-(methylamino)prop-1-en-1-yl)phosphonic acid (methyldehydrofosmidomycin). To an 18 mL test tube with stirbar was added **S6** (9 mg, 0.04 mmol) dissolved in 0.5 mL dH_2O. To this was added 0.5 mL of 12 N HCl. The reaction was refluxed for 24 h and then dried via rotary evaporation to yield 6 mg (0.04 mmol, quantitative yield) of methyldehydroFOM. Note that there is a small contaminant in the product. Given that this material was only used for spiking experiments to confirm the product of the reaction, we do not feel this is a major issue.
1H NMR (600 MHz, d2o) δ 6.46 (m, 1H), 6.23 (t, 1H, *J* = 17.1 Hz), 3.81 (d, 2H, *J* = 6.0 Hz), 2.75 (s, 3H).
31P NMR (243 MHz, d2o) δ 10.95.
13C NMR (151 MHz, d2o) δ 134.9 (d, *J* = 5.3 Hz), 130.0 (d, *J* = 177 Hz) , 50.2 (d, *J* = 24.0 Hz), 32.2. There was also a small contaminant of deuterated methanol.
HRMS (ESI) calcd for $C_4H_{11}NO_3P$ (M+H)+: 152.0477, found: 152.0471, Δppm: -3.9.

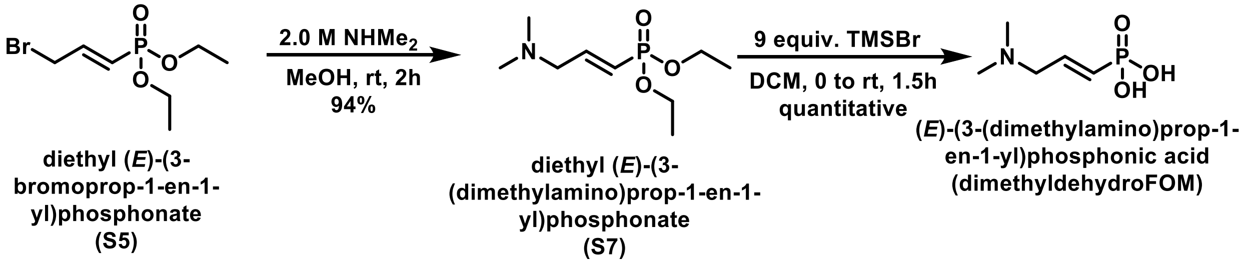

diethyl (E)-(3-(dimethylamino)prop-1-en-1-yl)phosphonate (S7). To an oven dried 20 mL flask with stirbar was added **S6** (104 mg, 0.40 mmol). The vial was evacuated and refilled three times with nitrogen. To it was added dimethylamine (1.2 mL of 2.0 M in methanol, 2.4 mmol, 6 equiv.), and the reaction was stirred at room temperature for 2h. The reaction was dried via rotary evaporation. The solid was then dissolved in 1 mL of dH_2O and loaded onto a 15.5 g C18Aq column. The following gradient was then run using a Combiflash Rf+ and a flow rate of 30 mL/min (Teledyne Isco): 1 min at 100% solvent A (dH2O), linear gradient to 50% solvent B (ACN) over 16 min, 2 min at 100% B, and 1 min at 80%B. Fractions were screened via TLC using $KMnO_4$ stain. Fractions containing product were combined and dried via rotary

evaporation to yield 84 mg of product (0.38 mmol, 94% yield). There was a minor impurity that was carried through the next reaction.

1H NMR (600 MHz, d2o) δ 6.81 (m, 1H), 6.45 (t, 1H, *J* = 18.8 Hz), 4.22 (m, 4H), 4.07 (d, 2H, *J* = 6.7 Hz), 2.98 (s, 6H), 1.38 (t, 6H, *J* = 7.1 Hz).

31P NMR (243 MHz, d2o) δ 17.08.

13C NMR (151 MHz, d2o) δ 140.2 (d, *J* = 5.8 Hz), 126.3 (d, *J* = 184 Hz), 64.1 (d, *J* = 5.8 Hz), 58.6 (d, *J* = 26.3 Hz), 42.8, 15.6 (d, *J* = 5.9 Hz).

HRMS (ESI) calcd for $C_9H_{21}NO_3P$ (M+H)+: 222.1259, found: 222.1255, Δppm: -1.8.

(E)-(3-(dimethylamino)prop-1-en-1-yl)phosphonic acid (dimethyldehydrofosmidomycin). To an oven dried 20 mL vial with stirbar was added **S7** (55 mg, 0.25 mmol). The vial was evacuated and refilled three times with nitrogen, and dry DCM (1.5 mL) was added. The solution was cooled to 0 °C, and bromotrimethylsilane (300 µL, 348 mg, 2.3 mmol, 9 equiv., Sigma-Aldrich) was added. The solution was allowed to gradually warm to room temperature and stirred for 2 h. The reaction was dried via rotary evaporation to yield an oil. The oil was then dissolved in dH_2O (3 mL) and stirred for 1h. It was dried drying via rotary evaporation to yield dimethyldehydroFOM (50 mg, quantitative yield).

1H NMR (600 MHz, d2o) δ 6.47 (m, 1H), 6.28 (t, 1H, *J* = 17.7 Hz), 3.85 (d, 2H, *J* = 6.8 Hz), 2.82 (s, 6H).

31P NMR (243 MHz, d2o) δ 12.04.

13C NMR (151 MHz, d2o) δ 135.2, 131.1 (d, *J* =178 Hz), 58.9 (d, *J* = 25.2 Hz), 42.6 (d, *J* = 3.6 Hz).

HRMS (ESI) calcd for $C_5H_{13}NO_3P$ (M+H)+: 166.0633, found: 166.0637, Δppm: 2.4.

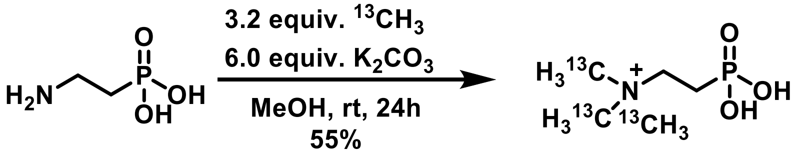

(2-aminoethyl)phosphonic acid (2AEP)

trimethyl-$^{13}C_3$-2-aminoethylphosphonate (^{13}C-trimethyl-AEP)

N,N,N-tri(methyl-13C)-2-phosphonoethan-1-aminium (trimethyl-$^{13}C_3$-2-aminoethylphosphonate). To an oven dried 20 mL vial was added 2AEP (63 mg, 0.5 mmol, Sigma-Aldrich), K_2CO_3 (823 mg, 6.0 mmol, 6.0 equiv.), and $^{13}CH_3I$ (100 uL, 0.22 g, 1.6 mmol, 3.2 equiv., Cambridge Isotope Laboratories). The vial was evacuated and refilled three times with nitrogen, and methanol (10 mL) was added. The resulting white slurry was stirred at room temperature for 24 h. The solution was dried via rotary evaporation. The resulting white solid was dissolved in 0.1% AcOH (40 mL) and loaded onto Fe-Chelex. The Fe-Chelex resin was generated by washing Chelex 100 sodium form (10 g, Sigma-Aldrich) with dH_2O (4 X 40 mL), 1 M HCl (1 X 30 mL), dH_2O until neutral, 150 mM $FeCl_3$ (40 mL), and 0.1% AcOH (2 X 40 mL). All washes except the iron wash were 5 min. The iron wash was 30 min. After washes were complete, the compound in 0.1% AcOH was loaded onto the resin (30 min incubation at room temperature). The following series of washes was performed (40 mL for each wash): 0.1% AcOH, dH_2O, 10 mM NH_4HCO_3, 1 M NH_4HCO_3, 1 M NH_4HCO_3, 1 M NH_4OH. Each wash was dried via rotary evaporation and screened via ^{1}H NMR. Washes containing product were combined and dried via rotary evaporation to give ^{13}C-TM-2AEP (47 mg, 0.27 mmol, 55% yield).

1H NMR (600 MHz, d2o) δ 3.55 (m, 2H), 3.28 (d, 9H, *J* = 144 Hz), 2.14 (m, 2H).
31P NMR (243 MHz, d2o) δ 16.76.
13C NMR (151 MHz, d2o) δ 62.8, 52.5 (t, *J* = 4.1 Hz), 23.2 (d, *J* = 128 Hz).
HRMS (ESI) calcd for $^{12}C_2{}^{13}C_3H_{15}NO_3P$ (M+): 171.0890, found: 171.0885, Δppm: -2.9.

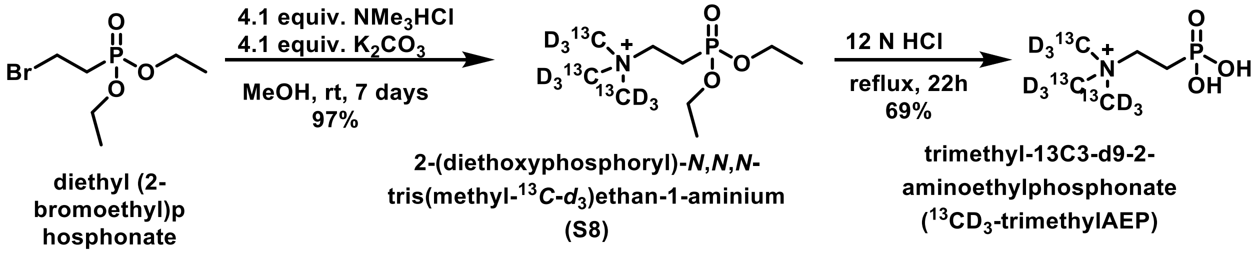

2-(diethoxyphosphoryl)-N,N,N-tris(methyl-13C-d3)ethan-1-aminium (S8). Diethyl 2-bromoethylphosphonate (149 mg, 0.61 mmol, Sigma-Aldrich), $^{13}CD_3$-trimethylamine-HCl (267 mg, 2.48 mmol, 4.1 equiv. Cambridge Isotope Laboratories), and K_2CO_3 (345 mg, 2.50 mmol, 4.1 equiv., Sigma-Aldrich) were added to an oven dried 20 mL vial with stirbar. The vial was evacuated and refilled three times with nitrogen, and MeOH (1.5 mL) was added. The slurry was stirred for 7 days at room temperature. The reaction was then dried via rotary evaporation. The solid was then dissolved in 1 mL of dH_2O and loaded onto a 15.5 g C18Aq. The following gradient was then run using a Combiflash Rf+ and a flow rate of 30 mL/min (Teledyne Isco): 1 min at 100% solvent A (dH_2O), linear gradient to 50% solvent B (ACN), 2 min at 100% B, and 1 min at 80%B. Fractions were screened via TLC using $KMnO_4$ stain. When run in EtOAc, Rf of starting material = 0.34. The product does not move from baseline. Fractions containing product were combined and dried via rotary evaporation to yield 142 mg of product (0.60 mmol, 97% yield). There is a slight impurity that was pushed through the next reaction.
1H NMR (600 MHz, d2o) δ 4.25 (m, 2H), 3.67 (m, 2H), 2.61 (m, 2H), 1.40 (t, 6H, *J* = 7.1 Hz).
31P NMR (243 MHz, d2o) δ 27.22.
13C NMR (151 MHz, d2o) δ 64.1 (d, *J* = 6.7 Hz), 59.9, 51.8 (sep. *J* = 22.5 Hz), 20.1 (d, *J* = 139 Hz), 15.6 (d, *J* = 5.8 Hz).
HRMS (ESI) calcd for $NO_3P^1H_{14}{}^2H_9{}^{12}C_6{}^{13}C_3$ (M+): 236.2081, found: 236.2089, Δppm: 3.4.

(2-(trimethylammonio)ethyl)phosphonate (trimethyl-$^{13}C_3$-d_9-2-aminoethylphosphonate). To a 50 mL round bottom flask with stirbar was added **S8** (140 mg, 0.60 mmol) and concentrated HCl (6 mL). The solution was refluxed for 22 h. The solution was dried via rotary evaporation. This gave $^{13}CD_3$-TM-2AEP (91 mg, 0.42 mmol, 69% yield).
1H NMR (600 MHz, d2o) δ 3.50 (m, 2H), 2.23 (m, 2H).
31P NMR (243 MHz, d2o) δ 20.83.
13C NMR (151 MHz, d2o) δ 61.2, 51.6 (sep., *J* = 21.8 Hz), 22.2 (d, *J* = 133 Hz).
HRMS (ESI) calcd for $^{12}C_3{}^{13}C_2{}^1H_7{}^2H_9NO_3P$ (M+): 180.1500 found: 180.1497, Δppm: -1.7.

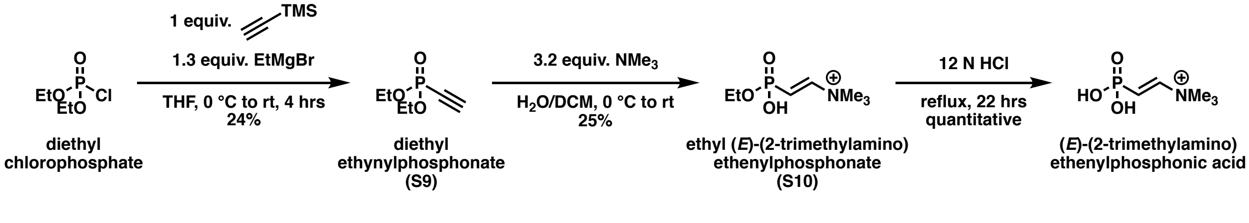

Diethyl ethynylphosphonate (S9). Trimethylsilyl acetylene (0.826 mL, 5.80 mmol, Oakwood Chemical) was added to dry THF (19.2 mL) in a flame-dried 50 mL round bottom flask with stirbar

under nitrogen at 0 °C. Then, EtMgBr (2.51 mL, 7.53 mmol, 3 M in THF, Sigma-Aldrich) was added dropwise. The mixture was removed from the ice bath and stirred at room temperature for 2 hrs. The flask was cooled to 0 °C once more and diethyl chlorophosphate (1 g, 5.80 mmol, Sigma-Aldrich) was added dropwise and the reaction was stirred for 1 hr, removed from the ice bath, and stirred at room temperature for 1 hr. The reaction was quenched with sat. aq. NH_4Cl (5 mL) and the solids were filtered and washed with Et_2O (2 x 5 mL). Organics were dried over anhydrous Na_2SO_4, filtered, and concentrated. The crude oil was left in a freezer for 2 months (-20 °C), and then purified on column chromatography (50% EtOAc in hex to 70% EtOAc in hex) to yield 230 mg of product (1.42 mmol, 24% yield) as a clear, colorless oil.

1H NMR (400 MHz, D_2O) δ 4.18 (p, J = 7.7 Hz, 4H), 2.88 (d, J = 13.2 Hz, 1H), 1.38 (t, J = 7.1 Hz, 6H).

31P NMR (200 MHz, D_2O) δ -8.36.

13C NMR (126 MHz, D_2O) δ 87.70 (d, J = 50.7 Hz), 75.70, 63.64 (d, J = 5.5 Hz), 16.18 (d, J = 6.9 Hz).

HRMS (ESI) calcd for $C_6H_{11}O_3P$ $(M+H)^+$: 163.0524, found: 163.0525, Δppm: 0.6.

Ethyl (E)-(2-trimethylamino)ethenylphosphonate (S10). To a 20 mL vial with stirbar was added **S9** (50 mg, 0.31 mmol), de-ionized H_2O (0.53 mL), and DCM (0.05 mL). The flask was cooled to 0 °C and trimethylamine (0.065 mL, 0.478 mmol, 7.3 M in H_2O, Acros Organics) was added dropwise. The reaction was removed from the ice bath and vigorously stirred at room temperature for 3 hrs. Once TLC indicated completion (2:1 EtOAc:hexanes, visualize by $KMNO_4$), the reaction was washed with DCM (3 x 1 mL) and the aqueous was concentrated until a viscous orange oil was left. Residual H_2O was azeotroped with dioxanes (3 x 3 mL) and a light yellow solid formed. The solid was triturated with MeCN (3 x 1 mL) until the yellow color disappeared to give 15 mg of product (0.08 mmol, 25% yield) as a white solid.

1H NMR (400 MHz, D_2O) δ 6.78 (t, J = 14.4 Hz, 1H), 6.47 (dd, J = 15.0, 5.7 Hz, 1H), 4.00 – 3.90 (m, 2H), 3.37 (s, 9H), 1.28 (t, J = 7.1 Hz, 3H).

31P NMR (160 MHz, D_2O) δ 8.34.

13C NMR (100 MHz, D_2O) δ 145.72 (d, J = 14.3 Hz), 119.84 (d, J = 167.6 Hz), 61.63 (d, J = 5.4 Hz), 54.04, 15.72 (d, J = 6.3 Hz).

HRMS (ESI) calcd for $[C_7H_{17}NO_3P]^+$ (M^+): 194.0946, found: 194.0946, Δppm: -0.24.

(E)-(2-trimethylamino)ethenylphosphonic acid. To a 2 dram vial with stirbar was added **S10** (15 mg, 0.07 mmol) and concentrated HCl (0.7 mL). The solution was refluxed at 100 °C for 22 h and dried via rotary evaporation to yield 14 mg of product (0.07 mmol, quantitative yield) as a white solid.

1H NMR (400 MHz, D_2O) δ 6.78 (t, *J* = 14.4 Hz, 1H), 6.48 (dd, *J* = 14.7, 5.6 Hz, 1H), 3.32 (s, 9H).

31P NMR (160 MHz, D_2O, pH = 1.5) δ 7.22.

13C NMR (100 MHz, D_2O) δ 145.31, 120.45 (d, *J* = 171.5 Hz), 54.05 (d, *J* = 18.3 Hz).

HRMS (ESI) calcd for $[C_5H_{13}NO_3P]^+$ (M^+): 166.0633, found: 166.0628, Δppm: -2.99.

Supplemental Figure 1

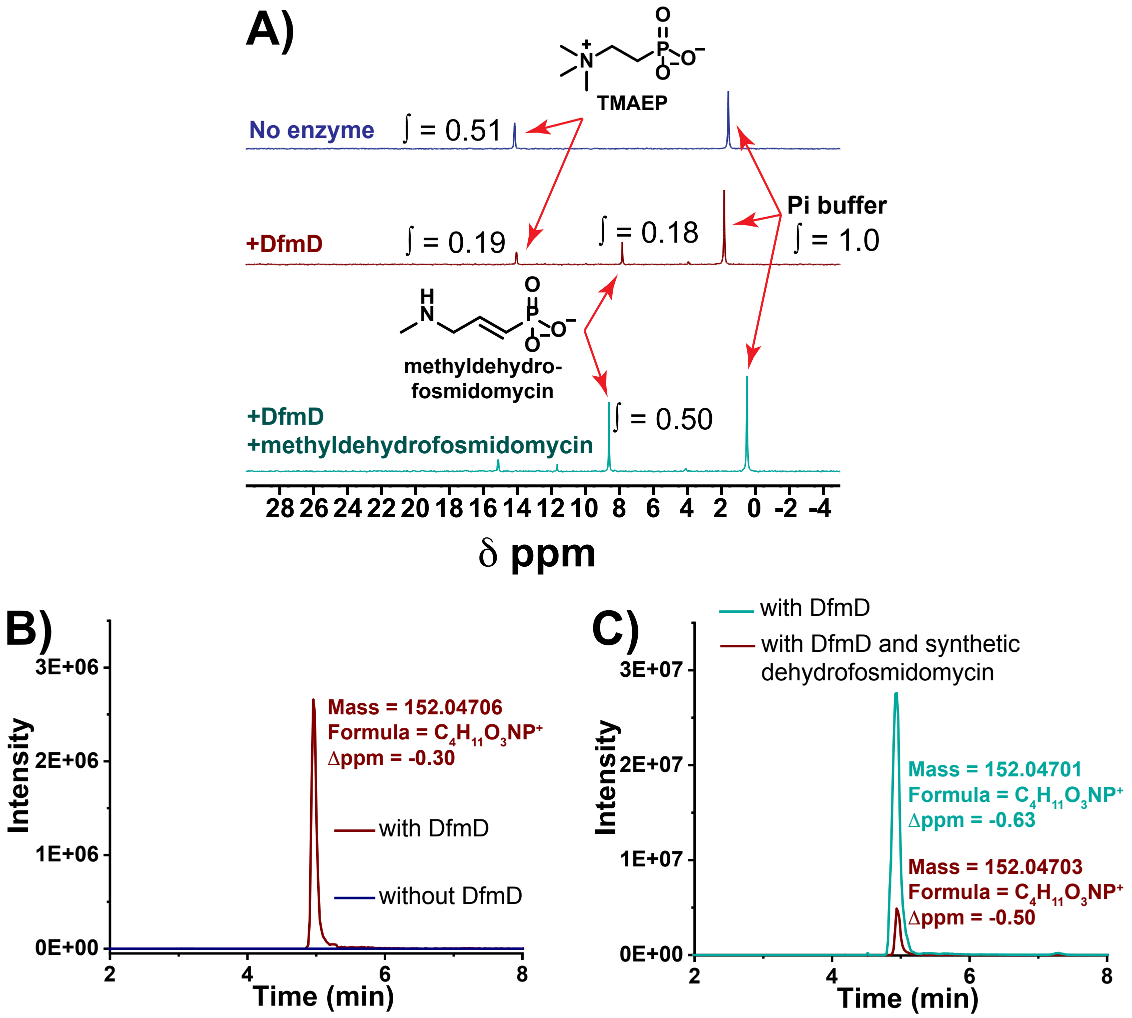

Fig S1. Methyldehydrofosmidomycin is the main product of the DfmD catalyzed reaction. *Panel A:* ^{31}P NMR spectra of the reaction of TMAEP without enzyme added (blue), with enzyme added (red), and with enzyme added and spiked with synthetic methyldehydrofosmidomycin (teal). The reactions were run for 24 h at room temperature as described in Fig 2. Integrations of each peak are relative to the phosphate buffer. *Panel B:* Extracted ion chromatrograms (EIC) from the HR-LC-MS analysis of the reactions shown in panel A with (brown) and without (blue) enzyme. *Panel C:* Extracted ion chromatrograms (EIC) from the HR-LC-MS analysis of the reactions shown in panel A with enzyme (brown) and with enzyme and spiked with synthetic methyldehydrofosmidomycin (teal).

Supplemental Figure 2

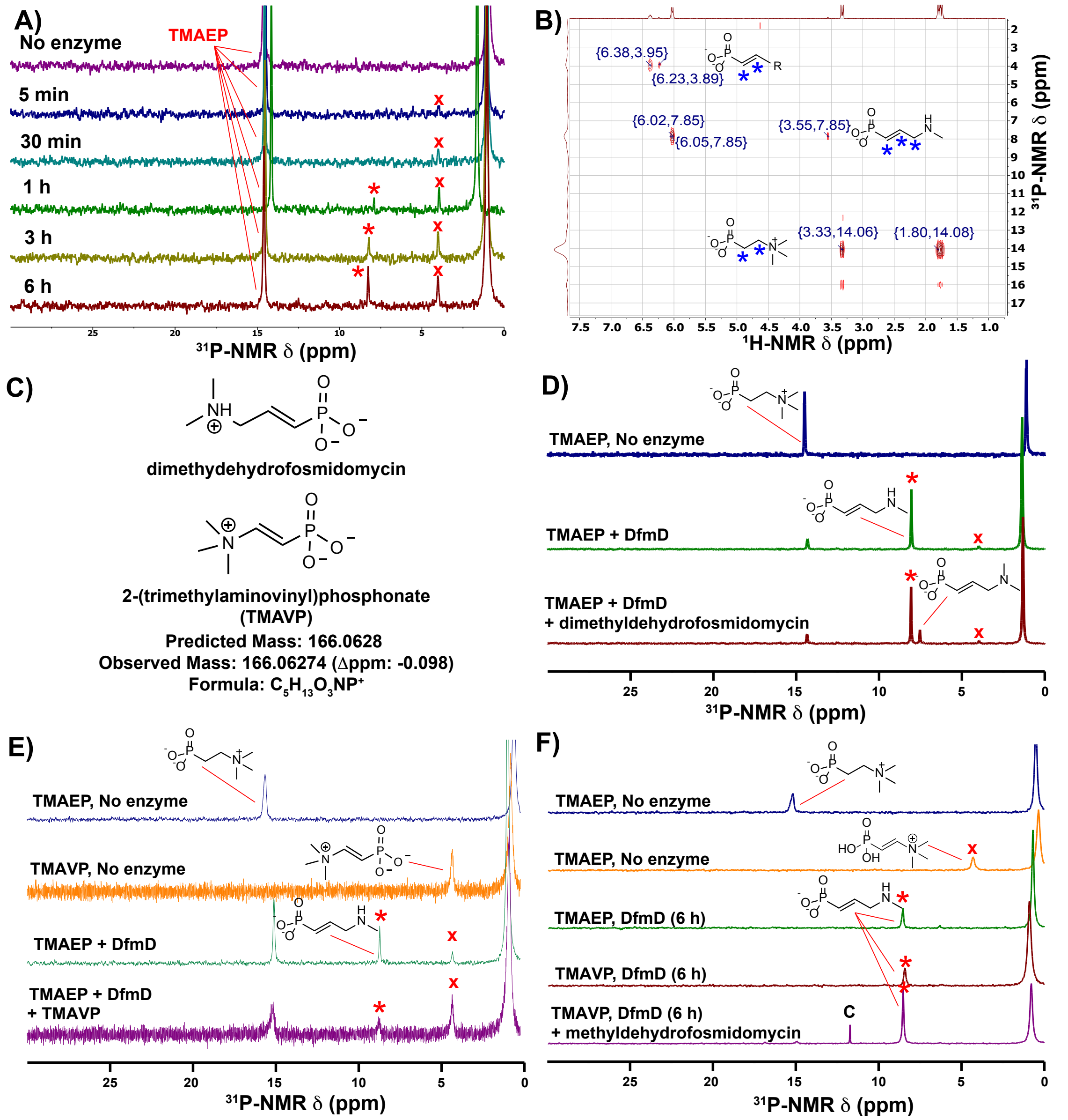

Fig S2. Identification of TMAVP as an intermediate in the DfmD reaction. *Panel A:* A time course showing the ^{31}P NMR analysis of the DfmD reaction. The peak labeled with an asterisk was shown to be methyldehydrofosmidomycin (see Fig S1). The peak labelled with “X” is the putative intermediate. *Panel B:* ^{1}H:^{31}P HMBC NMR analysis of the DfmD reaction with structures of the molecules giving rise to signals shown adjacent to each set of cross peaks. The ^{1}H cross-peaks at 6.38 and 6.23 for the putative intermediate suggest the presence of a vinyl moiety adjacent to the C-P bond. *Panel C:* Structures of vinylphopshonates that are consistent with the 166.0628 Da product observed in HR-LC-MS analysis of the DfmD reaction. Panel D: ^{31}P NMR of a no enzyme control (blue line), the full DfmD reaction (green line), and the full DfmD reaction spiked with dimethyldehydrofosmidomcin. Panel E: ^{31}P NMR of a no enzyme control with TMAEP (blue line), no enzyme control with TMAVP (orange line), the full DfmD reaction using TMAEP(green line), the full DfmD reaction using TMAEP, spiked with TMAVP after the reaction was complete. Panel F: ^{31}P NMR of a no enzyme control with TMAEP (blue line), no enzyme control with TMAVP (orange line), the full DfmD reaction using TMAVP(green line), the full DfmD reaction using TMAVP, spiked with methyldehydrofosmidomycin after the reaction was complete. “C” indicates a contaminant in the synthetic standard.

Supplemental Figure 3

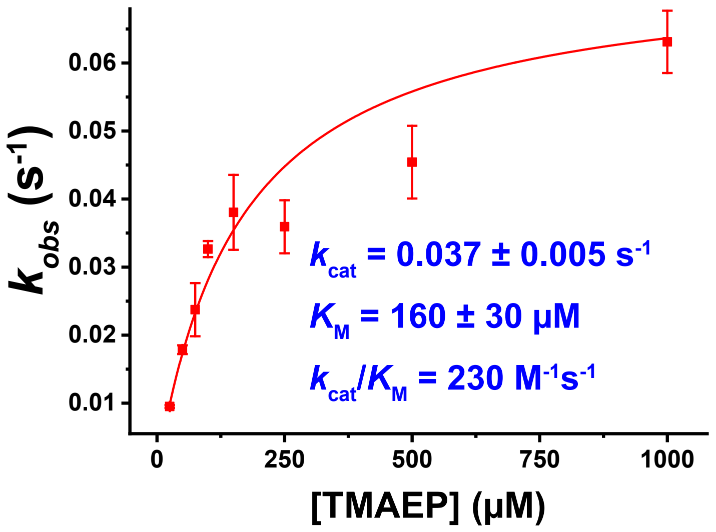

Fig S3. Kinetics analysis of the DfmD reaction. The rate of oxygen consumption was measured using a Clark-type electrode during the DfmD-catalyzed reaction (6.5 μM DfmD with 46 μM Fe (II)) with 10 mM 2-oxoglutarate and varying concentrations of TMAEP (25 – 1000 μM) at saturating concentrations of O_2 (280 μM). Kinetic parameters were determined by fitting the data to standard Michaelis-Menten parameters using OriginPro software. n = 3, error bars = standard error of the mean.

Supplemental Figure 4

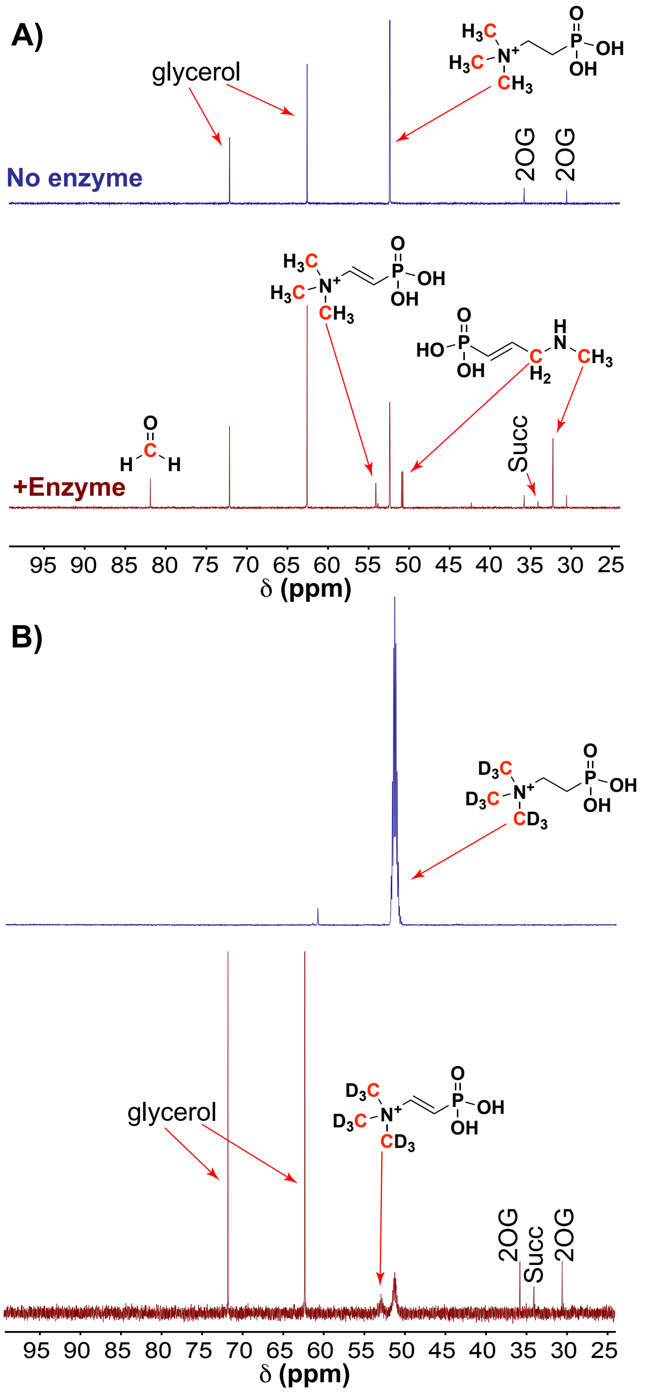

Fig S4. ^{13}C NMR analysis of DfmD activity with isotope labelled substrates. Panel A: Trimethyl-$^{13}C_3$ trimethyl-2-aminoethylphosphonate was incubated without (dark blue) or with (red) DmfD for 24 h prior to analysis. The structures of the compounds giving rise to each peak are also shown, with labelled carbons (shown in red) pointing to their respective signals. B) Analysis of trimethyl-$^{13}C_3$-d_9-2-aminoethylphosphonate alone (blue) or after incubation with DfmD for 24h by ^{13}C NMR. Representative data from 3 independent experiments. The enzyme storage buffer contains 10% glycerol accounting for the presence of these signals. Abbreviations: succinate (Succ), 2-oxoglutarate (2OG).

Supplemental Figure 5

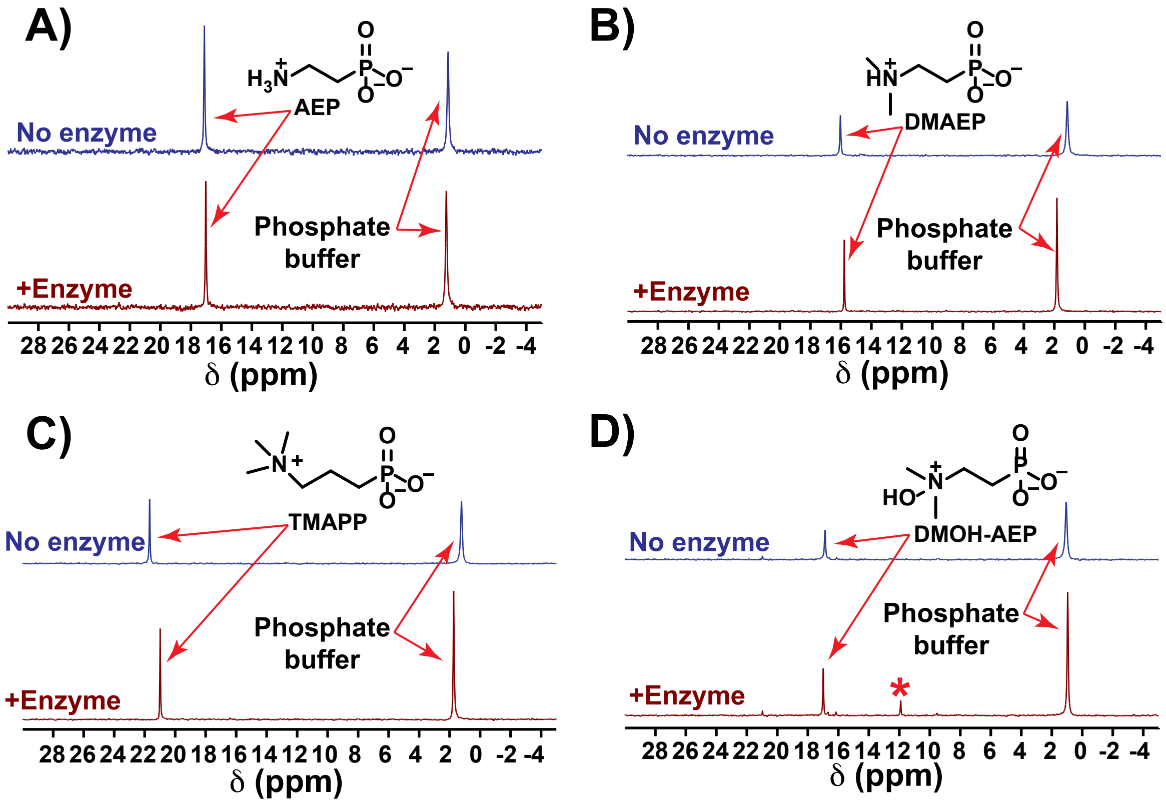

Fig S5. Substrate scope of DfmD. Each phosphonate substrate was incubated with (+Enzyme, red spectra) or without DfmD (No enzyme, blue spectra) for 6 hours, then analyzed by ^{31}P NMR. The structures of the compounds giving rise to each signal are also shown. *Panel A:* 2-aminoethylphosphonate (AEP), *Panel B:* dimethyl-2-aminoethyl-phosphonate (DMAEP), *Panel C:* trimethyl-3-aminopropylphosphonate (TMAPP) an *Panel D:* dimethyl-hydroxy-2-aminoethylphosphonate (DMOH-AEP). Only the DMOH-AEP reaction produced a product (marked with an asterisk), which was shown to be vinylphosphonate (Fig S6).

Supplemental Figure 6

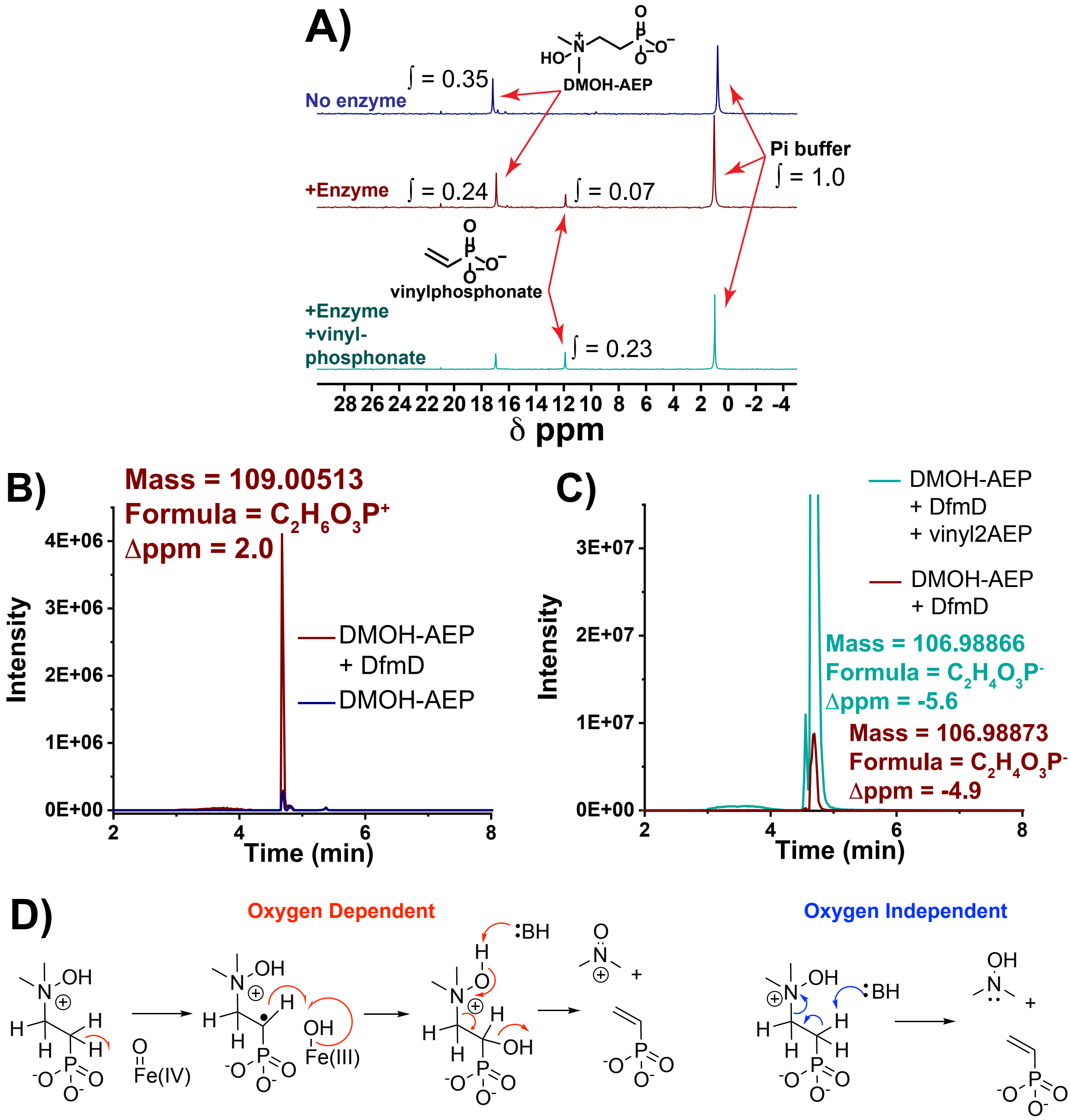

Fig S6. Vinylphosphonate is the sole product of the DfmD catalyzed reaction using dimethylhydroxy-2-aminoethylphosphonate as substrate. *Panel A:* ^{31}P NMR spectra of the reaction of dimethylhydroxy-2-aminoethylphosphonate (DMOH-AEP) without enzyme added (blue), with enzyme added (red), and with enzyme added and spiked with synthetic vinylphosphonate (teal). The reactions were run for 6h at room temperature. Integrations of each peak are relative to the phosphate buffer. *Panel B:* Extracted ion chromatrograms (EIC) from the HR-LC-MS analysis of the reactions shown in panel A with (brown) and without (blue) enzyme. *Panel C:* Extracted ion chromatrograms (EIC) from the HR-LC-MS analysis of the reactions shown in panel A with enzyme (brown) and with enzyme and spiked with synthetic vinylphosphonate (teal). Panel D: Putative mechanisms for production of vinyl phosphonate from DMOH-AEP.

Supplemental Figure 7

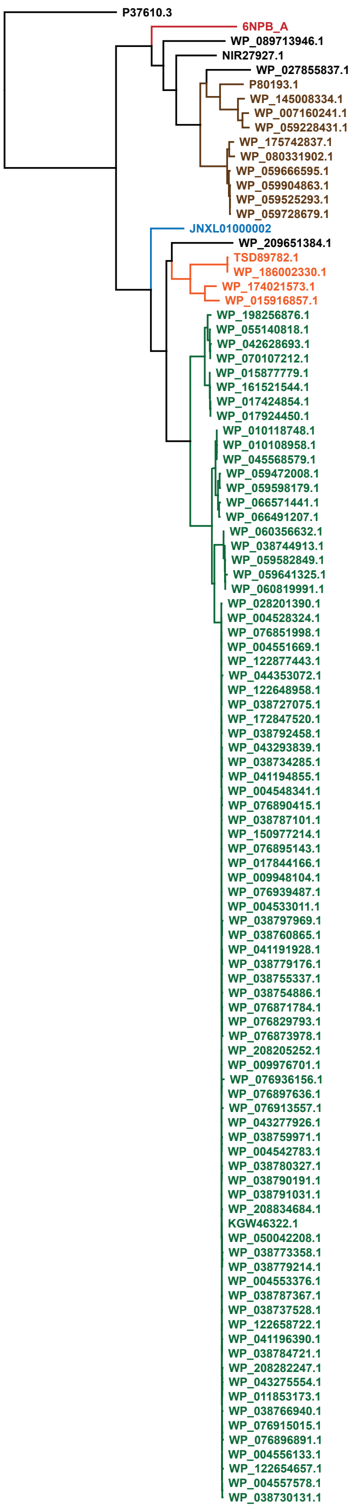

Fig S7. The accession numbers for the proteins depicted in Fig 1. Color coding and tree topology is identical to Fig 1.

Supplemental Figure 8

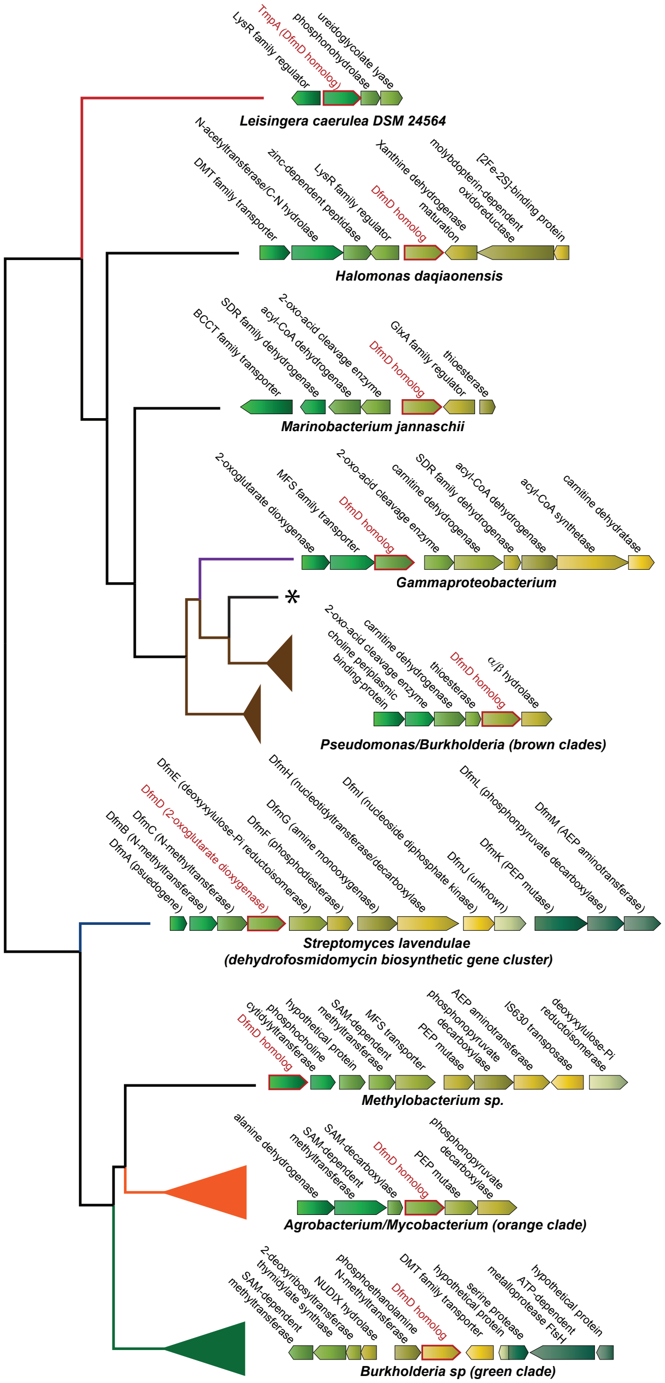

Figure S8. Gene neighborhoods of DfmD homologs. The gene neighborhoods of the top 100 DfmD homologs are presented next to a phylogenetic tree of the proteins. The branching pattern and color coding are identical to that shown in Fig 4. Nearly identical neighborhoods are shown only once. The putative functions of each gene, as found in the existing genome annotations or defined by conserved domain searches, are shown above the genes. The genome of *Pseudomonas sp.* AK-1(marked with an asterisk) has not been sequenced so the genomic context is unknown.

Supplemental Figure 9

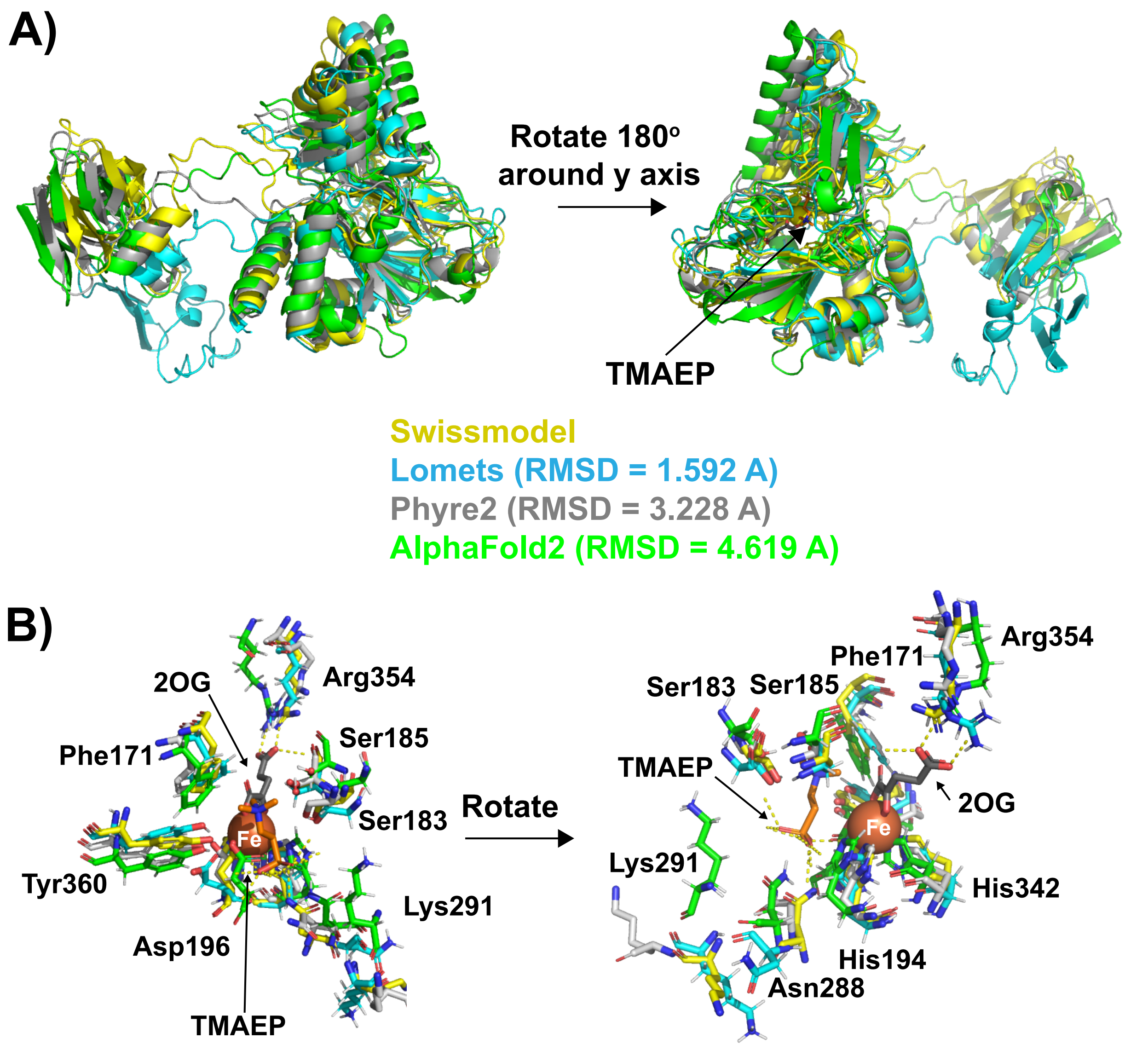

Figure S9. Comparison of DfmD model structures. *Panel A:* Superimposed models of DfmD made using Swissmodel (gold), Lomets (light blue), Phyre2 (grey) and AlphaFold2 (green). RMSD values relative to the Swissmodel are shown. *Panel B:* Closeup of the active sites in each model using the same color scheme as panel A.

Supplemental Figure 10

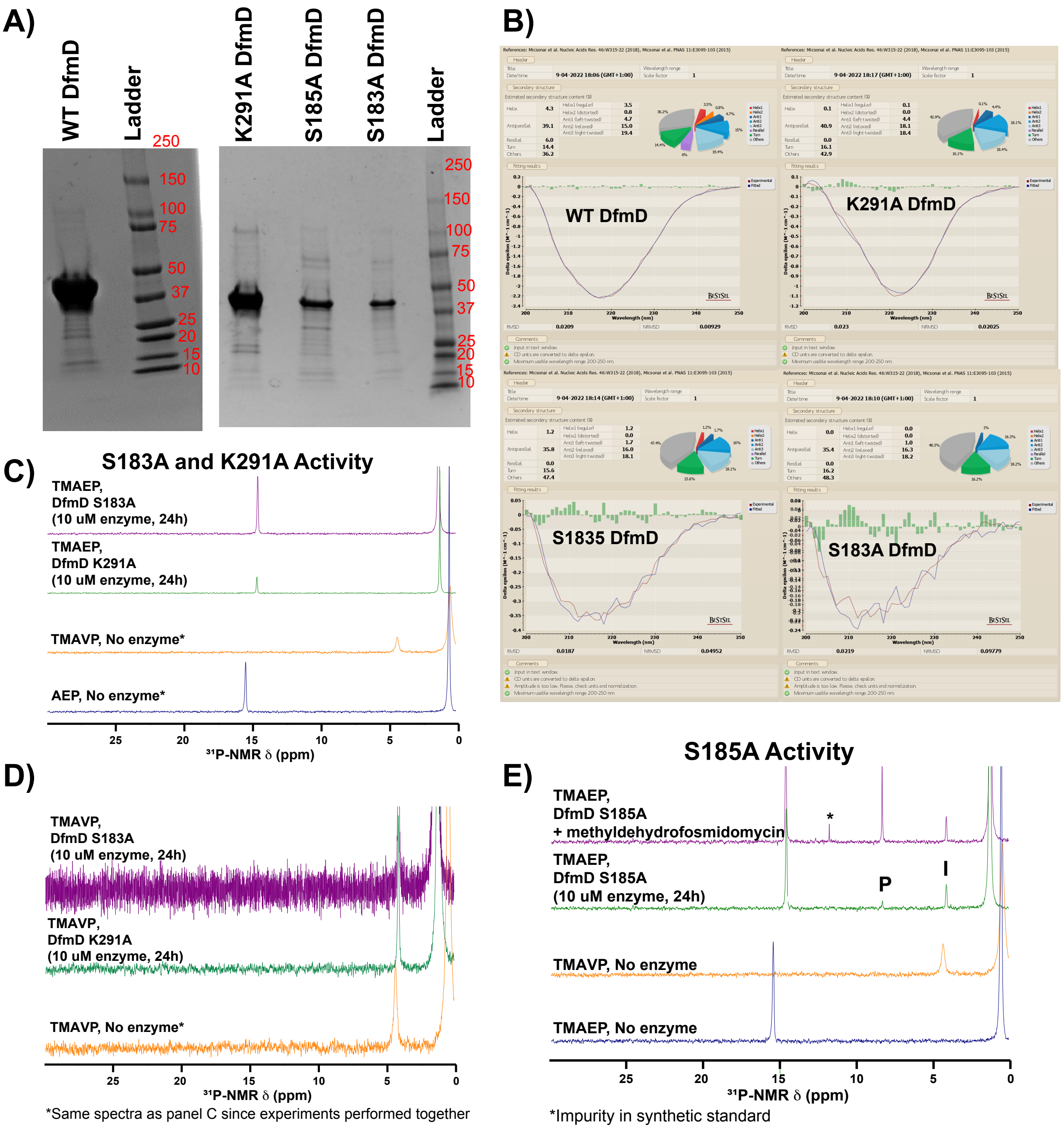

Figure S10. Purification and enzymatic activity of DfmD mutants. *Panel A:* SDS-PAGE analysis of purified DfmD, DfmD-K291A, DfmD-S185A and S183A. *PanelB:* CD analysis of purified proteins analyzed with BeStSel. *Panel C:* ^{31}P-NMR analysis of DfmD-K291A, and DfmD-S183A reactions and controls using TMAEP as substrate. *Panel D:* ^{31}P-NMR analysis of DfmD-K291A, and DfmD-S183A reactions and controls using TMAVP as substrate. *Panel E:* ^{31}P-NMR analysis of DfmD-S185A reactions and controls using TMAEP as substrate.

Supplemental Table 1

Stoichiometry of the DfmD reaction[a]

2OG consumed	Succ produced	TMAEP consumed	TMAVP produced	MDF produced	Ratio 2OG/Succ	Ratio 2OG/2MDF +TMAVP	Ratio Succ/2MDF +TMAVP
5.39	6.31	2.06	0.09	2.21	0.85	1.31	1.52
6.03	6.10	2.39	0.09	2.12	0.99	1.51	1.53
5.55	6.07	2.22	0.08	2.01	0.91	1.46	1.59
				**AVG**	**0.92**	**1.43**	**1.55**
				**STD**	**0.07**	**0.10**	**0.04**

[a]The molar equivalents of substrates consumed and products formed were calculated by subtracting the concentrations of the indicated compounds from those found in no enzyme controls after a six-hour reaction. Concentrations were determined via quantitative ^{1}H-NMR (as described above). Data from three replicates are shown, along with averages and standard deviation of the substrate/product ratios calculated as indicated in the headings of each column. Abbreviations used: 2-oxoglutarate (2OG); succinate (Succ); 2-(trimethylamino)ethyl-phosphonate (TMAEP); 2-(trimethylamine)vinylphosphonate (TMAVP); methydehydrofosmidomycin (MDF).

Supplemental References

1. Altschul, S. F.; Madden, T. L.; Schaffer, A. A.; Zhang, J.; Zhang, Z.; Miller, W.; Lipman, D. J., Gapped BLAST and PSI-BLAST: a new generation of protein database search programs. *Nucleic Acids Res* **1997,** *25* (17), 3389-402.
2. Edgar, R. C., MUSCLE: a multiple sequence alignment method with reduced time and space complexity. *BMC Bioinformatics* **2004,** *5*, 113.
3. Stamatakis, A., RAxML version 8: a tool for phylogenetic analysis and post-analysis of large phylogenies. *Bioinformatics* **2014,** *30* (9), 1312-3.
4. Parkinson, E. I.; Erb, A.; Eliot, A. C.; Ju, K. S.; Metcalf, W. W., Fosmidomycin biosynthesis diverges from related phosphonate natural products. *Nat Chem Biol* **2019,** *15* (11), 1049-1056.
5. Bisgaard, P.; M∅lhave, L.; Rietz, B.; Wilhardt, P., Quantitative Determination of Formaldehyde In Air Using the Acetylacetone Method. *Analytical Letters* **1983,** *16* (17-18), 1457-1468.
6. Micsonai, A.; Wien, F.; Bulyaki, E.; Kun, J.; Moussong, E.; Lee, Y. H.; Goto, Y.; Refregiers, M.; Kardos, J., BeStSel: a web server for accurate protein secondary structure prediction and fold recognition from the circular dichroism spectra. *Nucleic Acids Research* **2018,** *46* (W1), W315-W322.
7. Tars, K.; Leitans, J.; Kazaks, A.; Zelencova, D.; Liepinsh, E.; Kuka, J.; Makrecka, M.; Lola, D.; Andrianovs, V.; Gustina, D.; Grinberga, S.; Liepinsh, E.; Kalvinsh, I.; Dambrova, M.; Loza, E.; Pugovics, O., Targeting carnitine biosynthesis: discovery of new inhibitors against gamma-butyrobetaine hydroxylase. *J Med Chem* **2014,** *57* (6), 2213-36.

GenBank-formatted sequence of the dehydrofosmidomycin biosynthetic gene cluster.

```
LOCUS       JNXL01000002 ext       15394 bp    DNA     linear   UNA 28-OCT-2021
DEFINITION  Streptomyces lavendulae strain Fujisawa #8006 contig2.1, whole
            genome shotgun sequence.
ACCESSION   JNXL01000002
KEYWORDS    .
SOURCE      Streptomyces lavendulae
  ORGANISM  Streptomyces lavendulae
            Bacteria; Actinobacteria; Actinobacteridae; Actinomycetales;
            Streptomycineae; Streptomycetaceae; Streptomyces.
FEATURES             Location/Qualifiers
     source          <1..>15394
                     /organism="Streptomyces lavendulae"
                     /mol_type="genomic DNA"
                     /strain="Fujisawa #8006"
                     /db_xref="taxon:1914"
                     /collection_date=2010
     CDS             100..612
                     /product="DfmA (pep mutase pseudogene)"
                          /translation="MALSELTRDRLLRGRSVIPSRTMARHGLRELFEQP
                          QIVRVVGAHDAPGAHLRARAGFGAIGSSCLQATASHGVPDAD
                          LSTMSESLDTAHWTPQRWMHRWSPTATRTTATSSNAHITRPY
                          EAAGAAADPRCHGNRLGSRPGPSELTLQPRSRSACGARRRARS
                          RVIPVPGS"
     CDS             698..1534
                     /product="DfmB"
                          /translation="
                          MGSLVVTSVPQSDAPKTLEAQETIGQRFSRHGALRSEKIYGHG
                          YQGPSGFDVFSRLAAKIAWAPGMRVLDVGAGLGGDAFRMAD
                          QFGARVTGLDISPDMTEICRQRAADGGVTGVEFVTGDVRTADL
                          REAAYDVIWTRDCGMYLPLADKYRVWERLVSLLRPGGQVLIT
                          DYCRGAGKASAAFEQHVRDCGHDLLTLDGYRQVLESAKFTGL
                          AVEDQTEELLASMRGELARLEADRPKFLAEFTEDEYRALVDR
                          WQKKIQFSSDHELVWMVMTARVSAS"
     CDS             1541..2455
                          /product="DfmC"
                          /translation="
                          MSDGRPPGADSEAILRGPSARTKRHQYQPPLGRAMSNNDSPRR
                          SDVGREYETTRYAVPHHILRSEVVYGEGFQSSGGLAAFQETVL
                          PELPLAPGSRLLDVGSGLGGAAFHLVAEYEVEVVGVDPAELMI
                          SITESRRSLMDPQDRTSFVLGDVFSDALEPESFDAIYSQDSLLYS
                          ADKVKLLARCAELLRPGGAIVINDFCRGGSTPDFEEYVESAGY
                          HLVAVPVYPELLAEAGFVDVAARDVSTQTANLLRRDLDAYLA
                          RAEHDPEIHQADVEHLVDRWHRKIGFMDVGALTQGLFRAIKPI
                          SN"
     CDS             2466..3599
```

/product="DfmD"
/translation="MTTTAEAYLSIDWGSGETGRYHWIWLRDSCLCET
CRNAFAKQKYFDSATLPLDIRPRSVTRSAENGLEIVWEDGHES
RYPDSWLREHSTPVKPSRPEQRWSPWSSAEVVADGTFAHADV
MADNKALVGALEHLFRYGLVVLRGTDAEDVDPDALCSRLAGF
VDRSYFGEYFDLEVKPDNRTDSISFSTRQLPLHTDIPYYSTPPDY
QFLFGLEVNDAATASQGGRTRFVDGVAAALSLKERDPEAFAV
LTSTEVIYRAEYGDAEKIYHHQTPVIHLNNDGEVVRLVNNPTK
MFFDNVPFDEVTGVYRAYSAFKALMDEEGRAYHHSWRQGDM
IIFDNRRIFHGREQFDQTGMRRKLRGGYFSEVELRARSRFADET
V"

CDS 3727..4890
/product="DfmE"
/translation="MTESTSEYGLDRPRRVAVIGVTGSVGRQAVEVIER
SPRLEMVGAVALRDAGGLAAAGARLGCEHLALIETATDDRRS
WADPTPERFLHAVKPDVVLNAVVGVAGLAWTLAAIDAGHDIA
LANKESLVAGGRVVNERLAASSSRLLPVDSEHAALHQLLEGH
RHHAQTLTITASGGPFRGRRWADLHDVTVEAAMAHPTWAMG
NKNTLDSATLVNKGLELIEASYMFDWPQERIEIAVQPRSVIHAA
ITLRDGTHVANVSPPDMRRSIAYALHHPECVDIGLPHVPLAGL
GTLELEPEPEDFPGLALSRAALRAQDHGGTAAFNAANEEAVAA
FIAGRIRFTDITEIIARALDDLAPTPVTTLADVLAADARARQTAR
AVISARAVSS"

CDS 4887..5669
/product="DfmF"
/translation="MTVLIGQLSDPHVQDPDVDPEALRRFTDALAGMA
AEVGPDGRLLVTGDLTANGRPAEYAAVARAIEATDRAVHVLP
GNHDDPALIARLVPAGPGEQRVDLGSRMPSRCDFGEVVVLML
DSVVAGHSHGELGAEQLEWVDAQLAAAPDTMHLVAVHHPPF
AIGVVGIDRVGLADAEAFAEVLRDHDNVGRLLTGHVHRAVIT
GFAGVVATSCPSVWRSLPLELRPDAPHRQGPFQDLGLLHLADP
ATV
VTHVVSMPTRSAG"

CDS 5805..7022
/product="DfmG"
/translation="MKRNFDLAMVGLGPAAIGLLVAAARTGRLEPLLD
GGVIAYEQRTDRLGGKLSEYELAGNSLADVFLESLDEPGSREL
LAGLGDTPEADALREWSGQYPPLPLAGAFAELVAQRLIEVLER
HPACEVRLGTAVTAVDVAAPGEITVHDSSGGAETVPAVLICCG
GEEFSDSGLVATLNTPAVPGHAVYIDPTTLAHADRVTVLGGSH
TAWGVAARALVDYPGAQITLVQHRMPKLYFATTAEKAAGY
AFDPQDDVCPLSDRVFRYGGLRGPARELAVRTLSGPVDQLRVI
ELPGEWTAETLHENGALPADVVVPCFGYRAVLPEIRMAGRPV
DRATADDLVGTHGTDTESKVGLFAYGMGAGPRPNPKSGGEPS
YRGRLHGVWVYQHDAGDVMMDRLTGWLGKQR"

CDS 7019..8884

/product="DfmH"
/translation="MKGSTPTRAIILAAGMGRRLGRDSDHRPKPLTPIA
GVPIVHRSMAALAAVGVTEAVVVTGHLDAVVRASLGDEFAGI
KVHYLYNERYHETGDAYSVWLAREWFDRSLYLVEGDIVLRED
AFATVSTAPRANIVFAGEPVRRLGGTVVKAAADGTLDRLCTD
REQGPGFSPDGWLKTSNVFLLDAEYLRDGFAPALDALVTAGP
DGDKAYYDYAIVDSLVADQWAVCTWGIDEWFEVDDPGDRLQ
AEYLFSPPRRQRELLSESGGAYWRFDVRDHRSLRNAGFPTPEM
YESLTAPLRELLVDSPADHGLSTELVASLYGLHPEQVCVANCA
ADLLGPLCEAGGPIAVLEPGGDGHEAAVRSAQLVPFTLEPPQF
GLDVAAFAAFVREHGCSVAVIGSPNGKTGIAVPLSELRHLCKA
MPDVTVLVDESFVDFSMAPAGSLLPHLEEHPNLVVLRDLSEVY
GVGGIPLGFAATADPVRAAWLRTRLSTSKLSGLAEEFLRVLPH
YDKDFRRACALVSSDARALAAGLADLGPLTVVPPDANFVFAG
LPDGVSAAVVADELFTADRILVKAYSDTSDTVGRQWLRIASRG
RVADERLTSVLDVVLRRLTADSRNDVRPPGAEGH"

CDS 9027..9980
/product="DfmI"
/translation="MSIVWDALTRIPMKATLYEREIYFREGFADAEQIL
GDRFDELLRSTALMMLKPDGLLTGKLAPILEFLAEQRFTPVAA
QHVEFTPVLWREMWRYQLTSATLDRLAVTEHVYSTGPCLLLV
VRDDAPSSIPAAVRLSGLKGSSDPDVRRPDSLRARLELSNRIINH
LHVADEPADLVREIGLLLRPAERRRILRAVADGSMSERDRERL
RNEVDREVDKLYTLDVEGPLTRLYPKVQASLAGDPASVETAS
RVLACMERIRQGLIVPWRAFCADVESLGISVTDWDLAVLGSVS
VASDDPGATKVIDNPEAGLWGHDG"

CDS 9973..10932
/product="DfmJ"
/translation="MGDSVLDEGDWRALTMSPRKRELFVEDLYFRET
WEDLVQVAGGRAAEVLGPLALLAFKPDGVVGRRMRRTLAFIE
EEGFELVGFERVRYNRHSMRELWRYDWNLYTTDRLALSSLMH
AATDTFLLVLRDTAYTGVTPGTVRLADLKGSVLHPWGPRHLR
SVLQAPNKVIKFCHVADEPADLVRELGIFLDRGARRELLRGAE
EADASKVAASLLHEVGQLEAATIEHGFDIDETLGRLVGEGRLS
QRAEARIREVMAGGSRMSFDELASAMPPATDDQALWDFVLV
ASWVTPLERDGHVGVLPAPTAADWRGHRAPGSG"

CDS 11204..12808
/product="DfmK(PEP mutase)"
/translation="MTAAATRASEGITDGTSLRDLFERPGVVRIAGAHN
PLGARLAERAGFDGVWSSGLEVSASQGVPDTDILTMSELLAVA
GSLASAVSVPVVADCDAGYGNAHNVMNMIRRYEAAGISAVSI
EDKRFPKVNSFIPGRQELAPIGEFCGKLAAAKAAQRGSELMVIA
RIEALIAGWGMDEALLRGEAYADAGADAVLIHAKGSSPDPILE
FLNRWRLPVPVVVVPTTYHTITAAELGEAGAKMVIYANHGLR
AGITAVSRAFEAILREGRTTGIEEQIAPLATVFDLQGMPQQKQH
EKLYITPYGTSPRAFMVPGEQQQTGLDQRADVLVCQTAELRRS

GVEQVAVYAADQPPTRELQDVDVVAGVGTDAAAWVLSTPAN
YPGSTLVLPADVFLEAGPLRQLASNESDVAVLVDVSTRSGAAR
RPDAVGVSLSSSIRGGRRLTAGSSLVTGFGGSEVEAEFTGAAVF
SARGFAALVDAAEKRRAEGSTATVVELLADVLLGGLQVHAIE
VASGWTELRTADDLRYVGEAMATGGAER"

CDS 12805..13923
/product="DfmL (phosphonpyruvate decarboxylase)"
/translation="MTDTVSRLQAAELVNLLRDRGFGPFTGVPCSFLGP
VISCLQAEHPQDYLVAANEGEAVALAAGARLAGRRPVVILQNS
GLGNAVNPLTSLCHTLRLPVLLLVTWRGRPSTTDEPQHELMGR
ITPDLLTLMDVRNELLPSDPAVLAERLTAAEEHMESTGLPFAFV
VPKGSIAPYASEPSEETSGLMLRAEAIGHVVRAMDPDGCLIATT
GKTARELERDWDRPRNLYVVGSMGCASSVALGVALYSGQRG
VVVLDGDGAALMRLEAMATIGRQAPANLLHVLLDNESYESTG
GQPSGSGTVDFAGLALACGYAGAYDVSEPDELARAVADGQSL
PGPRLIRVRIAPGSHPQLGRPALQPPASAARFMESIAR"

CDS 13920..15035
/product="DfmL (AEPn aminotransferase)"
/translation="MKEASQLTGSDRLVLMNPGPVVTDHRVRAALAG
PDLCHREPEFADLMRSVRQRTTQLCAGDDRHTSVILTGSGTSA
VEAAISSVVPLDAGLLVIDNGHYGERFQHIAVSHNIRTHHLRLG
WDSPVDLDAVDRILSADSTLTHVGVVHHETSTGRLNDVAAVT
RIAHARGRQVIVDAVSSVGADAISLAGDEVDWLAGSSNKCVE
GAPGLSFVSGRHSAFEALGGLPRRTFYLDLHRHYTAQEKALAP
AFTPGIPAFYAFDTALGLALEEGREARHARYFALAQQLRTGLE
ELGLEIMLAPEERAVGLTAIRIPEGLTYEWLHDGMRASGFVIYS
AQEQLKNRFFRLSTMGCMSSRDITDFLNCLQRLLQQS"

ORIGIN
1 actcgccggg agttaaatca cttgagcccg gcggtgcgcc tgatgtagcg tccctcaaaa
61 ctctgatgat cttcatggct gcggggggct gtgggacgca tggcgttatc cgaattaacg
121 agagatcggc ttctgcgggg caggtcggtc atcccgagcc gaaccatggc ccgccacggg
181 ctgcgagaac tcttcgagca gccacagatc gtacgggtgg taggagcgca cgacgcgccg
241 ggcgcccatc tccgcgcccg agccggcttc ggcgcgatcg ggtccagctg tctgcaggcg
301 accgcctccc acggggtgcc tgacgcggac ctgtccacca tgagcgagtc gctcgacacc
361 gcccactgga cgccgcagcg gtggatgcac cgctggtcgc cgactgcgac acggactacg
421 gcaacgtcct caaacgcgca catcacacgg ccgtacgagg ccgccggcgc agcggccgat
481 ccgcgttgtc acggcaaccg gctcggctcc cgacctgggc cgtccgagct cactctccag
541 ccgagatcgc ggagcgcctg cggcgcacgc cggcgtgcca gatcacgggt gatccccgta
601 ccagggtcat agcctcgggc ggtttcggat acaccgttcg attccgctga caagcgctac
661 ttacacagac tgctgcgatg atccgcagcg cgacggcatg gggagtcttg tcgtgacatc
721 cgtaccacaa agcgatgcgc caaaaacgct ggaagcacag gagacgatcg ggcagcggtt
781 cagtcggcac ggcgccctgc ggtccgagaa gatctacgga cacggatacc agggtccctc
841 cggattcgac gtcttctcgc gcttggccgc gaagatcgct tgggcccccg gcatgcgcgt
901 tctggatgtc ggagccggcc tcggaggcga cgcgttccgc atggccgacc agttcggcgc
961 gagggtgacc ggactcgaca tctcgccgga catgaccgag atctgccggc agagagctgc

1021 cgacggtggg gtcacaggcg tcgagttcgt caccggcgac gtccggaccg cagacctccg
1081 cgaggctgcc tacgacgtca tctggaccag agactgcggg atgtacctgc cgctggccga
1141 taagtaccgc gtgtgggagc gcctcgtctc actgctgcga ccgggcgggc aggtgttgat
1201 caccgactac tgcaggggcg cgggaaaggc gtcggccgcc ttcgagcagc acgtccgtga
1261 ctgcggccac gatctgctca cgctggacgg gtaccgccag gtgctggagt cagcgaagtt
1321 caccggactg gcggtcgaag accagaccga ggagctgctc gcgtcgatgc gcggcgaact
1381 cgcccggctt gaggcggacc gccccaagtt cctcgcggag ttcaccgagg acgagtaccg
1441 ggcgctcgtc gaccggtggc agaagaagat ccagttcagc tcggaccacg aactggtctg
1501 gatggtcatg acagcgcgtg tctccgcgtc ctgagtcggc atgtcggacg gacggccgcc
1561 gggagcggac tccgaagcga tcctgcgcgg gccctcggcc cggaccaagc ggcaccaata
1621 ccagccacct ctagggaggg cgatgtcgaa caacgattcc ccacgtcggt cggacgtggg
1681 ccgggagtac gagaccaccc gatacgcggt gccgcaccac atcctgcggt ccgaggtcgt
1741 ctacggggag ggcttccaga gctccggcgg cttggccgcg ttccaggaga cggtcctgcc
1801 ggagctgccg ctggcacccg gtagccggct gctggacgtc ggctccggcc tcggcggcgc
1861 cgcgttccac ctggtcgccg agtacgaggt cgaagtcgtc ggggtcgatc ctgcggaact
1921 gatgatatcg atcacggaga gccgtcggtc gctcatggac ccccaggatc gtacgagttt
1981 cgtgctcggc gacgtcttct cggacgcact ggagccggag tccttcgacg cgatctacag
2041 ccaggattca ctgctctaca gcgcggacaa ggtgaagttg ctcgcccggt gcgccgaact
2101 gctccggccc ggtggggcga tcgtcatcaa cgacttctgc cgtggcggga gcacgccgga
2161 cttcgaagag tatgtcgaaa gcgccggata ccacctcgtc gccgttcccg tctatccgga
2221 gctcttggcg gaggcgggtt tcgtggacgt cgcggccagg gacgtcagta cgcagaccgc
2281 caacttgctg cggcgcgact tggacgccta cttggcccga gccgaacacg accccgagat
2341 ccaccaagca gatgtcgagc atctcgtgga ccggtggcac cgaaagatcg gattcatgga
2401 cgtgggagcg ctgacccagg gcctgttccg tgcaatcaag ccgatatcga actaaggaga
2461 cgaccatgac tacaacagcc gaagcatatc tgtcgatcga ctggggttcc ggcgagaccg
2521 ggcggtacca ctggatctgg ctccgagact cgtgcctgtg cgaaacctgc cgcaacgctt
2581 ttgccaaaca gaagtatttc gattcggcga ccctgccgct cgacatacgg cctcgaagcg
2641 tgacgcgcag cgcggagaac ggcctggaga tcgtctggga ggacggccac gagagccgtt
2701 acccggactc ctggctgcga gagcacagca caccggtgaa gccgagccgc cctgaacagc
2761 ggtggtcccc gtggtcgagc gccgaggtcg tggccgacgg caccttcgcc cacgccgacg
2821 tgatggccga caacaaggct ctcgtcggcg cgctggagca cctgttccgc tacggcctcg
2881 tcgtgctgag gggaacggac gcggaggacg ttgaccccga tgcgctgtgt tctcggttgg
2941 ccggcttcgt cgaccggtcc tacttcggcg aatacttcga tctggaggtc aagcccgaca
3001 accggaccga cagcatctcc ttcagcaccc gccagctgcc gctgcacacg gacatcccgt
3061 actacagcac gccccccggac taccagttcc tgttcggact cgaagtcaac gacgcggcca
3121 ccgccagcca gggcggacgg acccggttcg tggacggagt ggccgccgcg ctgagcctga
3181 aggaacggga ccccgaggcg ttcgccgtac tgaccagcac cgaggtcatc tatcggggccg
3241 agtacggcga tgccgagaag atctaccacc accagacccc ggtcatccac ctgaacaacg
3301 acggcgaagt cgtccgcttg gtgaacaacc ccaccaagat gttctttgac aacgtgcctt
3361 tcgacgaggt gacgggcgtc taccgggcct acagcgcatt caaggcgctg atggacgagg
3421 agggtcgcgc ctatcaccac tcctggcgac agggcgacat gatcatcttc gacaaccggc
3481 gcatcttcca cggccgggag cagttcgacc agaccggtat gcggcgcaag ctgcgcggag
3541 gctacttcag cgaggtcgag ctgcgcgccc ggtcgcgttt cgccgacgaa acggtgtagg
3601 actccgcctc ccgtgcgtcg cctcggcggc cgatcacctc aagcgacctg cacgtcgaat
3661 tcatgacacc tgcgggattc gacccaccgc tcatatccag ctccactcga tgacagaacg
3721 gcagccatga ccgaatccac atctgagtac ggtctcgacc gtccgaggcg agtagcggtc

3781 atcggagtga ccggttcggt cgggcgtcag gccgtcgagg tcatcgagcg ttccccacgg
3841 ctggagatgg tcggggccgt ggccctgcgc gacgccggcg gtctggccgc cgccggcgcg
3901 cgcttgggct gcgagcatct cgcactgatc gagaccgcga cggacgaccg gcggagctgg
3961 gcggatccga cgcccgaacg cttcctgcac gccgtcaagc cggacgtcgt gctcaatgcc
4021 gtggtcggtg tcgccggcct ggcgtggacc ctcgccgcga tcgacgcggg gcacgacatc
4081 gcgctggcga acaaggaatc actggtcgcc ggcgggcgtg tggtcaacga gcgcctggcg
4141 gccagttcgt cccggctcct gcccgtggac tccgagcacg ccgcgctgca ccaactgctc
4201 gaaggccacc ggcaccacgc acagaccttg accatcaccg catccggcgg ccccttccgc
4261 gggcggaggt gggccgatct gcacgacgtg accgtcgaag cggcgatggc gcaccccacc
4321 tgggcgatgg ggaacaagaa caccctcgac tccgcgaccc tggtcaacaa gggactcgag
4381 ttgatcgagg cgagctacat gttcgactgg ccgcaggaac ggatcgagat agccgtgcag
4441 ccccgctcgg tcatccacgc cgccatcacc ctgcgcgacg gcacgcacgt cgcgaacgtc
4501 tcgccgcctg acatgcggcg cagcatcgcc tacgcgctgc accacccgga atgcgtcgac
4561 atcgggctgc cccacgtgcc cctcgccgga ctcgggacgc tggaactcga acccgagccc
4621 gaggacttcc cgggcctcgc gctctcccgg gccgccctgc gcgctcagga ccacggcggc
4681 accgcggcgt tcaatgcagc caacgaggaa gccgtcgcag cgttcatcgc cgggcgcatc
4741 cggttcaccg acatcacaga gatcatcgcg cgcgccttgg acgacctggc cccgacgccg
4801 gtcaccacct tggccgacgt cctggccgcc gatgctcggg ccaggcagac ggcgcgggcc
4861 gtcatcagcg caagggccgt gtcgtcatga cggtcttgat cggtcagttg agcgacccgc
4921 acgtacagga cccggacgtc gatccggagg ccctgcggcg gttcaccgac gcgcttgcgg
4981 gaatggcggc cgaggtgggc ccggacggcc gcctgctggt caccggcgac ctcaccgcaa
5041 acggccggcc ggcggaatac gcggctgtgg cacgcgccat cgaggcgacc gaccgcgccg
5101 tacacgtgct ccccgggaac catgacgacc ctgcgctgat cgcccggctc gttcctgccg
5161 ggccgggggga gcagcgcgtc gacctgggtt cacgcatgcc gagccgctgt gacttcggcg
5221 aggtcgtggt cctcatgctg gattcggtgg tcgccggcca cagccacggg gagctcggcg
5281 ccgagcaact ggaatgggtc gacgcccagc tggcggccgc tcccgacacg atgcacctgg
5341 tcgccgtaca ccacccgccg ttcgccatcg gagtggtcgg catcgaccgt gtcggactcg
5401 ccgacgccga ggcgttcgcc gaagtgctgc gcgaccacga caacgtgggc cgcttgctca
5461 ccggccatgt gcaccgcgcg gtgatcaccg gcttcgccgg cgtcgtcgcg acctcctgcc
5521 cgtccgtctg gcgcagcctc ccgctggagc tgcgaccgga cgcaccgcat cgccagggcc
5581 cgttccaaga cctcggcctg ctgcacctgg cggaccccgc gaccgtcgtc acccacgtgg
5641 tgtccatgcc gacgcggtcg gccggctgag gagcggacct tgcgaagcat cagattcgcg
5701 tacatgtgcg accggcggcg tatgcgctgt ggtgtcgacg agggccgaac tcccggagtc
5761 cgccggctcc gggtggccgt tcactgtgat ctatcggagg acgtatgaag aggaactttg
5821 atctcgcgat ggtcggactc ggcccggccg cgatcggctt gctcgtggct gccgcccgca
5881 ccggacggtt ggaacccctg ctggacggtg gtgtgatcgc ctacgagcag cgcaccgatc
5941 ggctcggcgg caaactgtcc gagtacgaac tcgccggcaa cagcctcgcc gacgtgttcc
6001 tggagtctct ggacgagccc gggtcgcgcg aactcctcgc cggcctcggt gacacgcccg
6061 aggcggacgc cctgcgggag tggtcgggcc agtacccgcc gctgccgttg gccggggcgt
6121 tcgccgaact ggtggcacag cgtctcatcg aggtgttgga gcggcacccg gcctgcgagg
6181 tgcggctggg gacggcggtc acggcggtcg acgtggcggc tccgggagag ataacggtgc
6241 acgactcctc gggcggggcc gagacggtgc ccgccgtctt gatctgctgc ggcggcgagg
6301 agttcagcga ttccggcctg gtcgcgacgt tgaatacgcc cgctgtgccc ggtcacgccg
6361 tctacatcga tccgaccacg ctcgcccacg cggatcgggt gaccgtcctc ggcgggtcgc
6421 acaccgcctg gggcgtcgcg gcacgggcgc tcgtcgacta ccccggcgcg cagatcacgc
6481 tggtgcagca ccgcatgccg aagctctact tcgccaccac cgcagaggcg aaggccgcag

6541 gttacgcctt cgacccgcag gacgacgtct gcccgctcag cgaccgggtg tttcgctacg
6601 gcgggctgcg cggccccgcc cgagagctgg cggtgcgtac cctgagcggt ccggtggacc
6661 agctgcgggt gatcgagctg ccgggtgagt ggaccgcgga gacgttgcac gaaaacgggg
6721 ccttgccggc ggacgtcgtc gtgccgtgct tcggctaccg tgccgtgctg ccggagatcc
6781 gcatggccgg tcgcccggtg gaccgggcca ccgctgacga tctggtcggc acgcacggaa
6841 ccgacaccga aagcaaggtg ggactgttcg cctacgggat gggtgccggt ccccgaccca
6901 acccgaagtc cggcggcgag ccctcctacc ggggacgcct gcacggggtc tgggtctacc
6961 agcacgacgc cggcgacgtg atgatggacc gcctcacggg ctggttgggc aagcagaggt
7021 gaagggctcc acaccgactc gagcgatcat cctggccgcc ggcatggggc ggcggctggg
7081 gcgggactcg gaccaccggc ccaaaccgct caccccgatc gccggcgtac cgatcgtgca
7141 ccgcagcatg gccgcgctgg ccgcggtcgg ggtgaccgag gccgtcgtgg tcaccgggca
7201 cctcgacgcc gtggtgcgtg cctcgctcgg agacgagttc gccggaatca aggttcacta
7261 cctgtacaac gagcgctacc acgagaccgg ggatgcgtat tcggtctggc tcgcgcgcga
7321 gtggttcgac cgcagcctgt acctggtcga gggcgacatc gtgttgcgcg aggacgcgtt
7381 cgccacggtg tcgactgcgc cgagggccaa catcgtgttc gccggtgaac cggtgcggcg
7441 cttgggcggc accgtggtca aagccgctgc ggacggcacc ttggaccggc tgtgcaccga
7501 tcgggagcag ggaccgggct tctcgccgga tgggtggctc aagacgagca acgtcttcct
7561 gctcgacgcc gagtacctgc gagacggctt cgcccctgct ctcgacgcat tggtgaccgc
7621 cggcccggac ggcgacaagg cgtactacga ctacgcgatc gtggacagcc tcgtggccga
7681 ccagtgggcg gtgtgcacat ggggcatcga cgagtggttc gaagtggacg atcccggtga
7741 ccggctgcag gcggagtacc tgttcagccc gccgcgccgg caacgggaac tgctgtccga
7801 gtcgggcggt gcgtattggc ggttcgacgt ccgcgatcac cggtcgctgc ggaatgccgg
7861 cttcccgacg ccggagatgt acgagtcgct caccgcgccc ctccgcgagt tgctggtcga
7921 ctccccggcg gaccacggcc tgtcgacgga gctggtggca tcgctgtacg ggctgcaccc
7981 cgagcaggtg tgtgtggcga actgcgctgc cgatctgttg ggcccgctgt gcgaagcggg
8041 cggtccgatc gcggtgttgg agcccggtgg cgacggtcat gaggccgccg tccggtccgc
8101 gcagctggtg ccgttcacgc tggagccgcc tcagttcggg ctcgacgtgg ctgcctttgc
8161 cgcgttcgtg cgcgaacacg ggtgctcggt ggctgtgatc ggctccccga acggcaagac
8221 gggcatcgcc gtacccctgt cggagctgcg ccacctgtgc aaggcgatgc ccgacgtcac
8281 cgtcctcgtg gacgagtcgt tcgtggactt ctcgatggcg cccgccggct ccctgttgcc
8341 ccatctggaa gaacacccca acttggtggt gttgagggac ctgagcgagg tctacggtgt
8401 tggcggcatc ccgctcgggt tcgccgccac ggccgacccg gtccgcgcgg cctggttgcg
8461 cacccggctg tcgacctcga agctcagcgg cctggcagaa gagttcctcc gagtgctgcc
8521 gcactacgac aaggacttcc gccgcgcgtg cgcactggtg tcgtccgacg ctcgtgcact
8581 cgccgccggc ctggccgatc tcggcccgct gaccgtcgtt ccaccggacg cgaacttcgt
8641 gttcgccggg ctgcccgacg gcgtctcggc cgccgtggtc gcggatgagc tgttcaccgc
8701 ggatcggatc ctggtgaagg cctacagcga cacgtcggac accgtcggcc gacagtggct
8761 gcgcatcgcc agccgcggcc gggtggcgga cgagcggctg acgagcgtgc tcgacgtcgt
8821 gcttcggcgc ctgacggccg attcgcgaaa tgacgtccgc ccgccgggag cggaaggcca
8881 ttaacggacc gaaggctcga cgccctgaca gacgtgcggc acgttttatg agattgtgag
8941 cgatacgccg ttttcgttga tatgagattc agccggtaca cccgcgtcac tgttcgcgag
9001 gagcgagaaa ggcagaacca gaaatcgtgt cgatagtgtg ggacgctctg acgcggatac
9061 caatgaaggc caccctctat gagcgtgaaa tctacttccg tgagggcttc gcggacgccg
9121 aacagatcct cggagaccgt ttcgacgaac tgctgcgctc gactgcgttg atgatgctca
9181 agccggacgg actgctcacg ggcaagctgg caccgattct ggagttcctg gccgaacaac
9241 ggttcacgcc ggtcgcggcg cagcatgtcg agttcacgcc ggtgctctgg cgggagatgt

9301 ggcggtacca gctcacttcc gcgacgctcg atcgactcgc ggtgacagag cacgtctact
9361 cgaccgggcc ttgcctgctg ctcgtcgtgc gggacgacgc gccgtcatcg attccggcgg
9421 cggtccgcct gagcgggctg aaaggatcgt cggatcccga tgtgcggcgt cctgattcgc
9481 tgcgggccag gcttgaactc tccaatcgaa tcatcaatca ccttcatgtc gctgacgaac
9541 ctgccgacct cgtccgtgaa atcggcttgt tgctccgccc tgcggaacgt cggcggatcc
9601 tgcgtgccgt cgcggacggc tcgatgtcgg agcgtgaccg cgagcgcctt cgcaacgaag
9661 tggaccgcga ggtcgacaag ctctacacgt tggacgtgga gggccctctg accaggctgt
9721 acccgaaggt tcaggcgagc ctcgccgggg atcccgcgtc cgtcgagacg gcctcccgcg
9781 tccttgcgtg catggagcgg attcgccagg gtctgatcgt gccgtggcgt gcgttctgtg
9841 cggatgtcga gtccttgggc atcagcgtga ccgactggga cctcgccgtg ctcggcagcg
9901 tcagcgtggc cagtgacgat ccgggcgcga ccaaggtgat cgacaatccg gaagcggggc
9961 tgtggggaca cgatgggtga cagcgtgctg gatgaaggtg actggcgtgc cctgacgatg
10021 tcaccgcgca agcgggagct cttcgtcgag gacctctact tccgcgagac gtgggaggac
10081 ctggtgcagg tggccggtgg gcgggccgcc gaggtgctgg ggccgttggc cctgctggcc
10141 ttcaagccgg acggcgtggt gggtcggcgc atgcgccgca cgttggcttt catcgaggaa
10201 gagggtttcg aactcgtcgg cttcgagcgc gtgcgctaca accgtcactc gatgcgcgag
10261 ctctggagat acgactggaa cctgtacacc acggatcggc tggccctctc gtcgctgatg
10321 cacgccgcca ccgatacgtt cctgctcgtg ctccgcgaca ccgcctacac cggggtgacg
10381 ccggggacgg ttcgattggc ggacctcaag ggctccgtgc tccacccctg gggaccgcgg
10441 cacctgaggt ccgtgctcca ggccccgaac aaggtcatca agttctgcca tgtcgccgat
10501 gaaccggcgg atctcgtgcg cgaactcgga atcttcctcg accgcggggc caggagagaa
10561 ctgctccgcg gtgccgagga ggctgacgcg agcaaagtcg ccgcatcgct cctgcacgag
10621 gtgggccagc tcgaagcggc cacgatcgag cacggtttcg acatcgacga gaccctggga
10681 cggctggtgg gcgaaggacg gctctcccag cgcgccgagg ctcgcattcg ggaggtcatg
10741 gcgggcggat cgcgcatgtc gttcgacgag ttggcatcgg cgatgcctcc tgccacggac
10801 gaccaggcgt tgtgggactt cgtgctggtg gccagctggg tcacgcccct cgaacgggat
10861 ggtcacgtcg gagtactgcc ggctcccacg gctgccgact ggcgtggtca tcgcgcacca
10921 ggatccggat gatcactgtc cgacggcggt acccgcctca tcttccacgg caggttcctg
10981 cctcagcccc tccgaccatg gcggaagtac gggaaaattc cgcaagtgca gctttggaat
11041 tccatatgat cacaactcct gacgctatcc gatgatgatg tcggaagaac tagcgaaggc
11101 caattctcgg gcactccagg aattatttca aggaaactca tcatcaaggg tctcagccaa
11161 tgtcgactga ccccgcctcc tcattccccc aaggagtatc ctcatgaccg cggcagctac
11221 aagggcatca gagggcatca cagatggcac ctccctccgt gacttgttcg agcggccagg
11281 agtggttcgg atcgccggtg cccacaaccc cctgggtgcc cgactcgccg agagagccgg
11341 attcgacggc gtgtggtcca gcggactgga ggtttctgcc tcccagggag tcccggacac
11401 cgacatcctg acgatgagtg aactactggc tgtggcgggc tcgctggcct cggccgtgag
11461 cgttccggtg gtggcggact gtgacgcggg atacggcaac gcccacaacg tcatgaacat
11521 gatccgccgc tacgaggcgg ccggcatctc cgcggtctcc atcgaggaca agcggttccc
11581 caaggtcaac agcttcatac cggggcgtca ggaactcgct ccgatcgggg agttctgcgg
11641 gaagctggcg gccgcgaagg cggcgcagcg cggctccgaa ctgatggtga tcgcccggat
11701 cgaggcgctg atcgctggct ggggcatgga cgaggcgctt ctgcgcggtg aggcctacgc
11761 cgacgcgggc gccgacgcgg tgctcatcca cgccaagggg tcgtcccccg atcccatcct
11821 ggagttcctc aaccggtggc ggctcccggt gccggtcgtg gtcgtgccca ccacctatca
11881 cacgatcacc gccgcagagc tgggtgaagc cggcgcaaag atggtgatct acgccaacca
11941 cggtctgcgt gccgggatca ccgccgtcag ccgcgccttc gaggcgatcc tgcgcgaggg
12001 gcggaccacc ggaatcgaag agcagatcgc gcccctggcc accgtcttcg acctgcaagg

12061 catgccccag cagaagcagc acgagaagct ctacatcacc ccctacggca ccagcccccg
12121 ggccttcatg gtgcccggcg aacagcagca gaccggcctc gaccaacggg cggacgtcct
12181 cgtctgccag accgccgaac tccgtcgcag cggcgtggag caggttgccg tctacgctgc
12241 cgatcagccc ccgacccgcg aactgcagga cgtcgacgta gtcgccgggg tcggcacgga
12301 tgccgcggcc tgggtgctga gtacgcccgc gaactacccg ggctccaccc tggtcctgcc
12361 cgccgacgtc ttccttgagg ccggaccact gcgccagctc gcctcgaacg agtcggacgt
12421 cgcggtcctg gtcgacgtgt cgacgcggtc cggtgcggcc cgtcgtcctg acgcggtcgg
12481 ggtctccctg agttcaagca tccgtggcgg ccgcaggctc actgccggaa gcagtctggt
12541 caccggtttc ggcggcagcg aggtggaggc ggagttcacc ggcgctgcgg tcttctcggc
12601 acggggcttc gccgcgctgg tggacgcggc ggaaaagcgt cgggccgagg gatcgacggc
12661 gaccgtcgtc gaactgctgg ccgacgtcct gctcggcggg cttcaggtcc acgcgatcga
12721 ggtcgcctcc ggctggaccg aactccgtac cgccgacgac ctccggtatg tcggcgaagc
12781 catggcaacg ggcggggccg agcgatgacc gacactgtca gccggctgca ggccgcagaa
12841 ctggtcaacc tgctccgcga ccggggattc ggcccgttca ccggagtccc gtgctcgttc
12901 ctgggcccgg tgatcagctg cctgcaggcc gagcacccgc aggactacct ggtggcggcg
12961 aacgaagggg aagccgtcgc tctcgccgcg ggcgcccgtc tggcggggcg ccgacccgtg
13021 gtcatcttgc agaactcggg cctgggcaac gccgtgaacc cgctcacctc gctgtgtcac
13081 acgctgcggt tgccggtgct cctgctggtg acctggcggg gccggccgtc aacgaccgac
13141 gagccgcagc acgagttgat gggccggatc acccccgacc tgctcaccct catggacgtc
13201 cgcaacgagt tgcttccgtc cgacccggcc gtgctggcgg agaggttgac cgcggccgag
13261 gagcacatgg agtccacggg cctgccgttc gccttcgtcg tccccaaggg ctccatcgcc
13321 ccgtacgctt cggagccctc ggaggagacg tccggcctca tgcttcgagc cgaagccatc
13381 gggcacgtcg tgcgcgccat ggaccccgac gggtgcctga tcgcgaccac cggcaagacg
13441 gctcgggagc tggagcggga ctgggatcgc ccgcgcaacc tctacgtcgt cggttccatg
13501 ggctgcgcat ccagcgtcgc cctgggcgtg gcgctgtact ccgggcagcg cggtgtcgtc
13561 gtcctggacg gcgacggagc ggcgctgatg cgcctcgaag cgatggccac catcggtcgc
13621 caggcaccgg cgaacctgct ccatgtcctg ctggacaacg agtcctacga gtccaccggc
13681 ggtcagccca gtggttccgg caccgtggac ttcgccggcc tcgcgctcgc ctgcggctac
13741 gcgggcgcat acgacgtatc ggagccggac gagctggccc gggcggtcgc cgatggccag
13801 tcgctgcccg gcccacgtct catccgggtc cggatcgcgc cgggctccca tccgcagctc
13861 ggccgccctg ctcttcagcc acccgcttcg gcggcccgat tcatggagtc gatagcccga
13921 tgaaagaggc cagccagctc accggatccg accgcctggt cctgatgaac cccggcccgg
13981 tggtgaccga ccaccgggtc agggcggccc tggccggacc cgatttgtgc cacagggagc
14041 ccgagttcgc ggacttgatg cgcagcgtgc ggcaacggac cacacagctc tgcgccggcg
14101 acgaccggca cacctcggtg atcctgaccg gatcaggcac ctccgcagtg gaggccgcga
14161 tcagctcggt ggtcccactg gacgccgggc tgctggtgat cgacaacggt cactacgggg
14221 agcggttcca gcacatcgcc gtctcccaca acatccggac ccaccacctg aggctgggct
14281 gggattctcc ggtcgacctg gatgcggtcg accgcatcct cagcgcggac agcacgttga
14341 cgcacgtcgg cgtcgtgcac cacgagacca gcaccggtcg gctcaacgac gtcgccgccg
14401 tgacccggat cgcccatgcc aggggccgcc aggtgatcgt cgacgctgtg agcagtgtcg
14461 gtgccgacgc catctcgctg gccggcgacg aggtggattg gctggccgga tcatccaaca
14521 aatgtgtcga gggggctcct gggctcagct tcgtcagcgg tcgccactcg gccttcgaag
14581 cgctgggggg cctcccgcgt cggaccttct acctggatct gcaccgccac tacacggccc
14641 aggaaaaggc gctcgcgccc gcgttcacgc ccggcattcc cgccttctac gcctttgaca
14701 ccgcgctcgg gctggccctg gaggaggggc gcgaggcacg acatgcccgc tacttcgccc
14761 tggctcagca gctgcgtacc ggcttggagg agctagggct ggagatcatg ctcgccccgg

14821 aggagagggc ggtgggcctg accgcgatcc gcatccccga ggggctgacc tacgagtggt
14881 tgcacgacgg gatgcgcgcg agcggattcg tcatctacag cgcccaggag cagttgaaga
14941 accgcttctt ccggttgtcg accatggggt gcatgagcag ccgtgacatc accgacttcc
15001 tgaattgcct gcaacggctt cttcagcagt cctgatcgcg tggagaccgc cttgggatga
15061 gccgggcgcg cgaggcgctc actggccaag cggcgcccgg cacggaggtg gcgcgagccc
15121 ggtctgcggc gttcgtgtga agcacggccg tggccgggca gatggcatgc tccggctagt
15181 gctgtggccg ggtgtccacc ggcatcgcgt gggacgggcg ccggtcctgg ccccacaggg
15241 cagcgagggc acggcgggaa gtcgtgcggg aggggccgcc aggcgtagcc ggcccgcagc
15301 cggcacgcca aggcgtcggt gatctccctg cgggggtgct ccggtggccg cccgcccgct
15361 ttcggcggcg gcacgagcgg caccggcacc accc
//

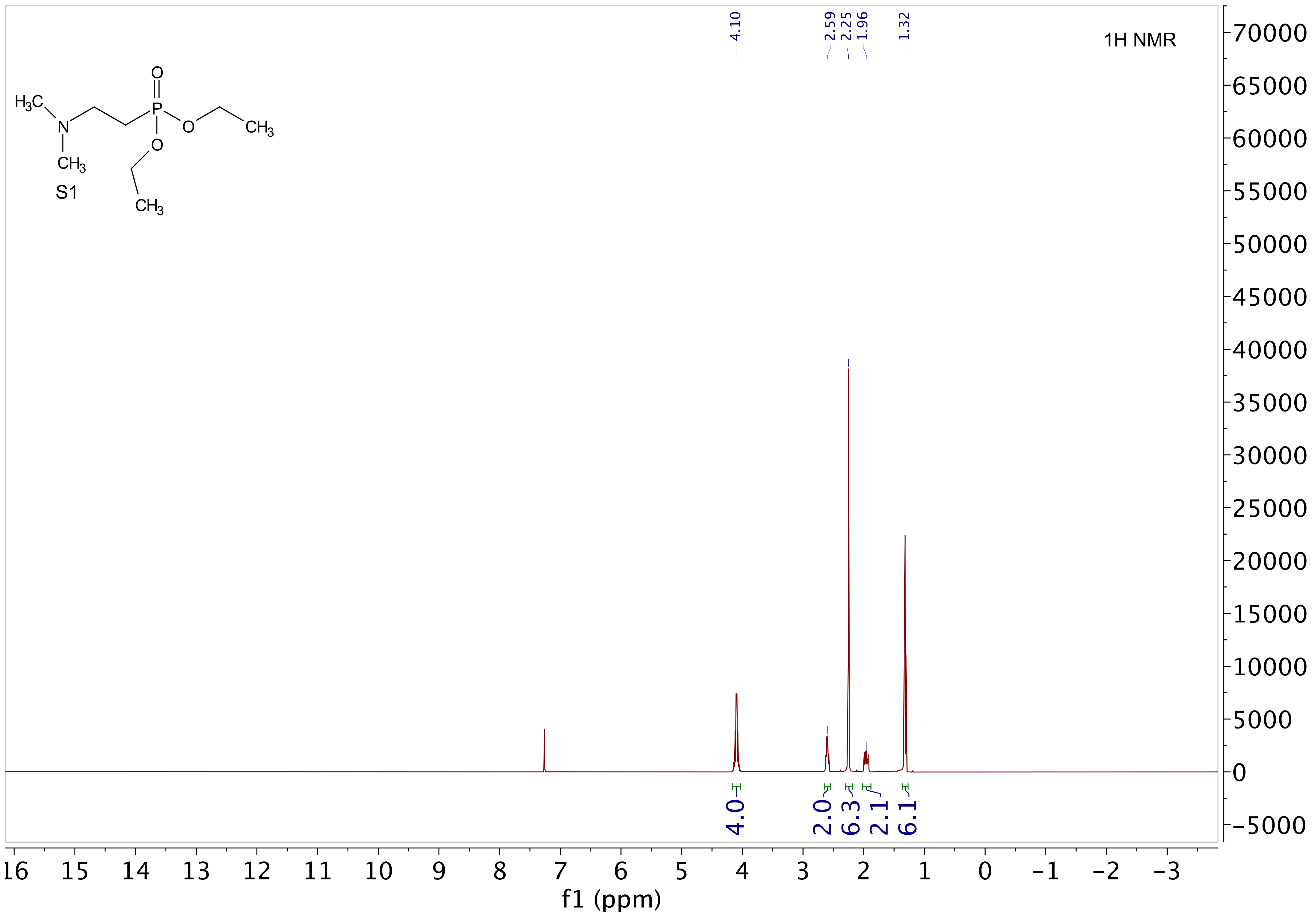

1H NMR
S1
4.10
2.59
2.25
1.96
1.32
4.0
2.0
6.3
2.1
6.1
f1 (ppm)

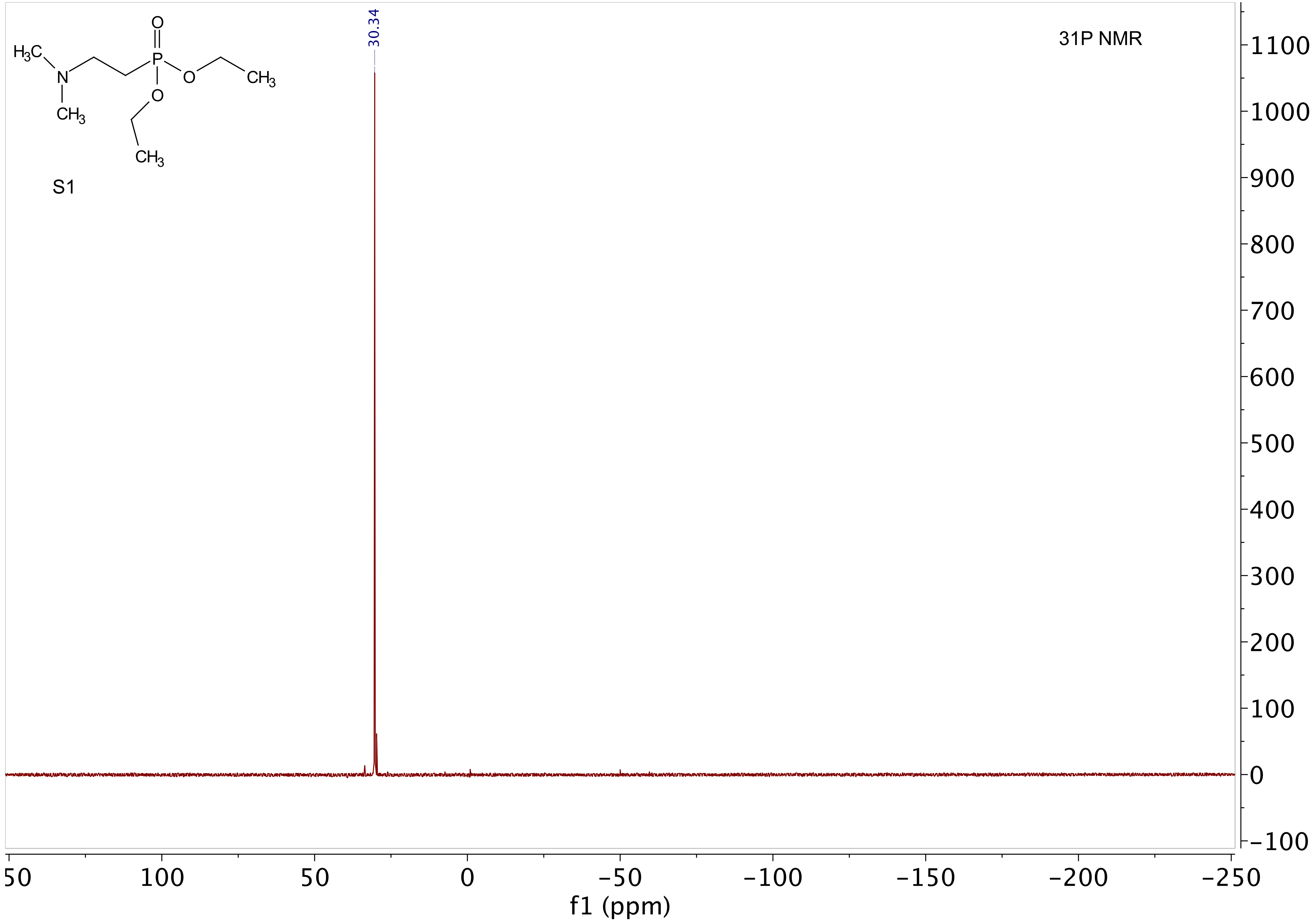
H3C
N
CH3
O
P
O
CH3
O
CH3
S1
30.34
31P NMR
1100
1000
900
800
700
600
500
400
300
200
100
0
−100
50
100
50
0
−50
−100
−150
−200
−250
f1 (ppm)

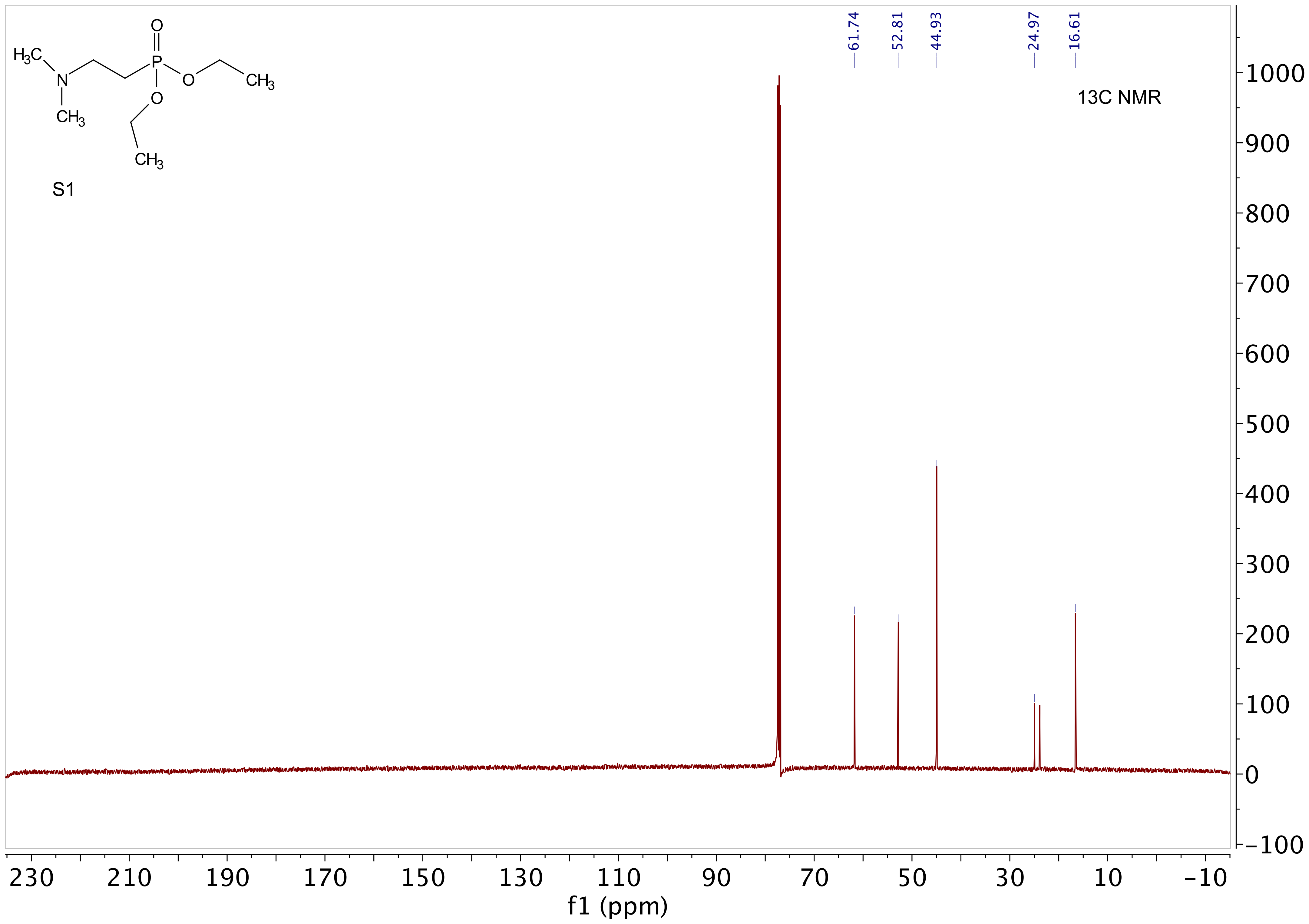
H3C
N
CH3
O
P
O
CH3
O
CH3
S1
13C NMR
61.74
52.81
44.93
24.97
16.61
1000
900
800
700
600
500
400
300
200
100
0
−100
230
210
190
170
150
130
110
90
70
50
30
10
−10
f1 (ppm)

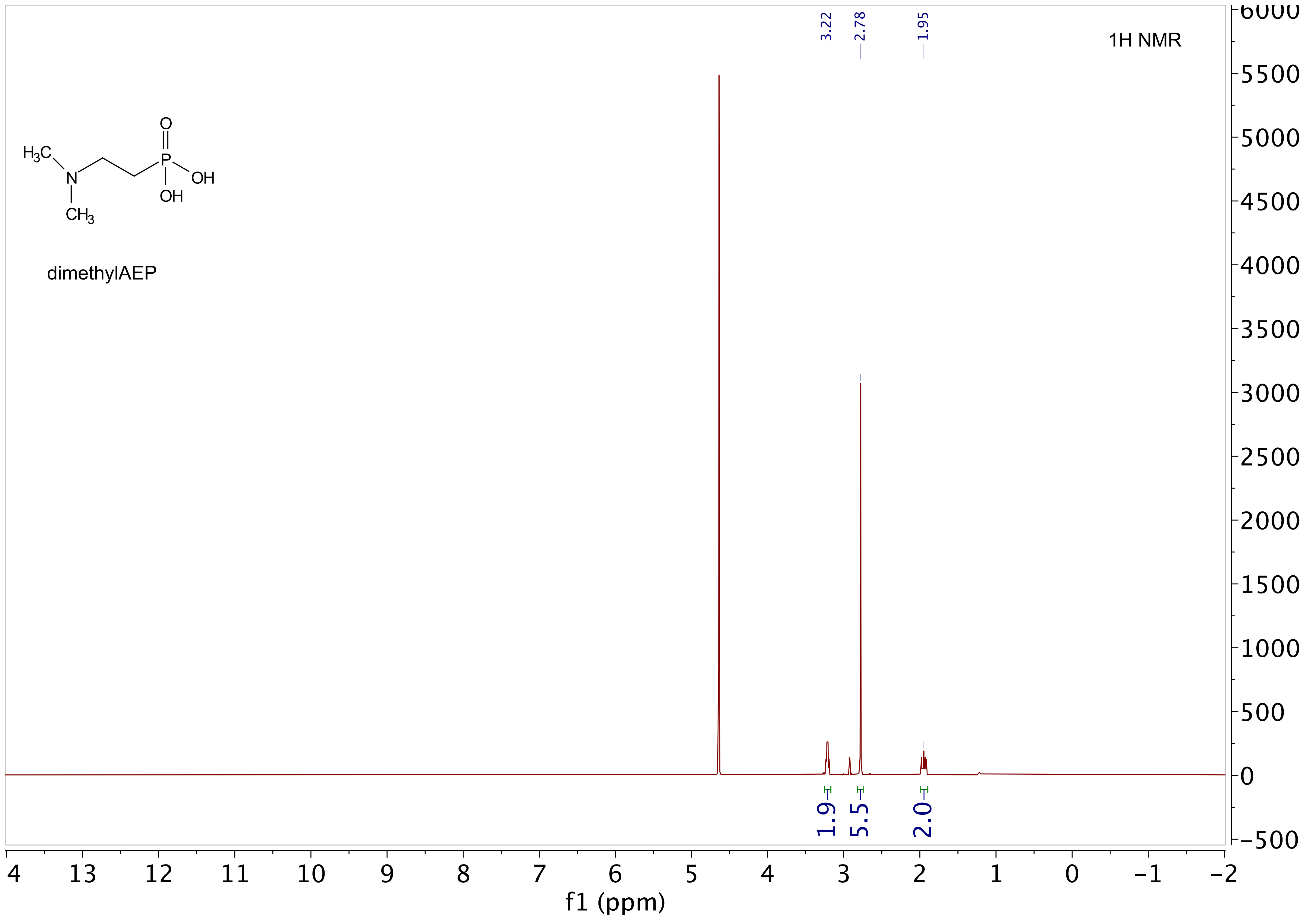
1H NMR
dimethylAEP
H3C
N
CH3
P
O
OH
OH
3.22
2.78
1.95
1.9
5.5
2.0
f1 (ppm)

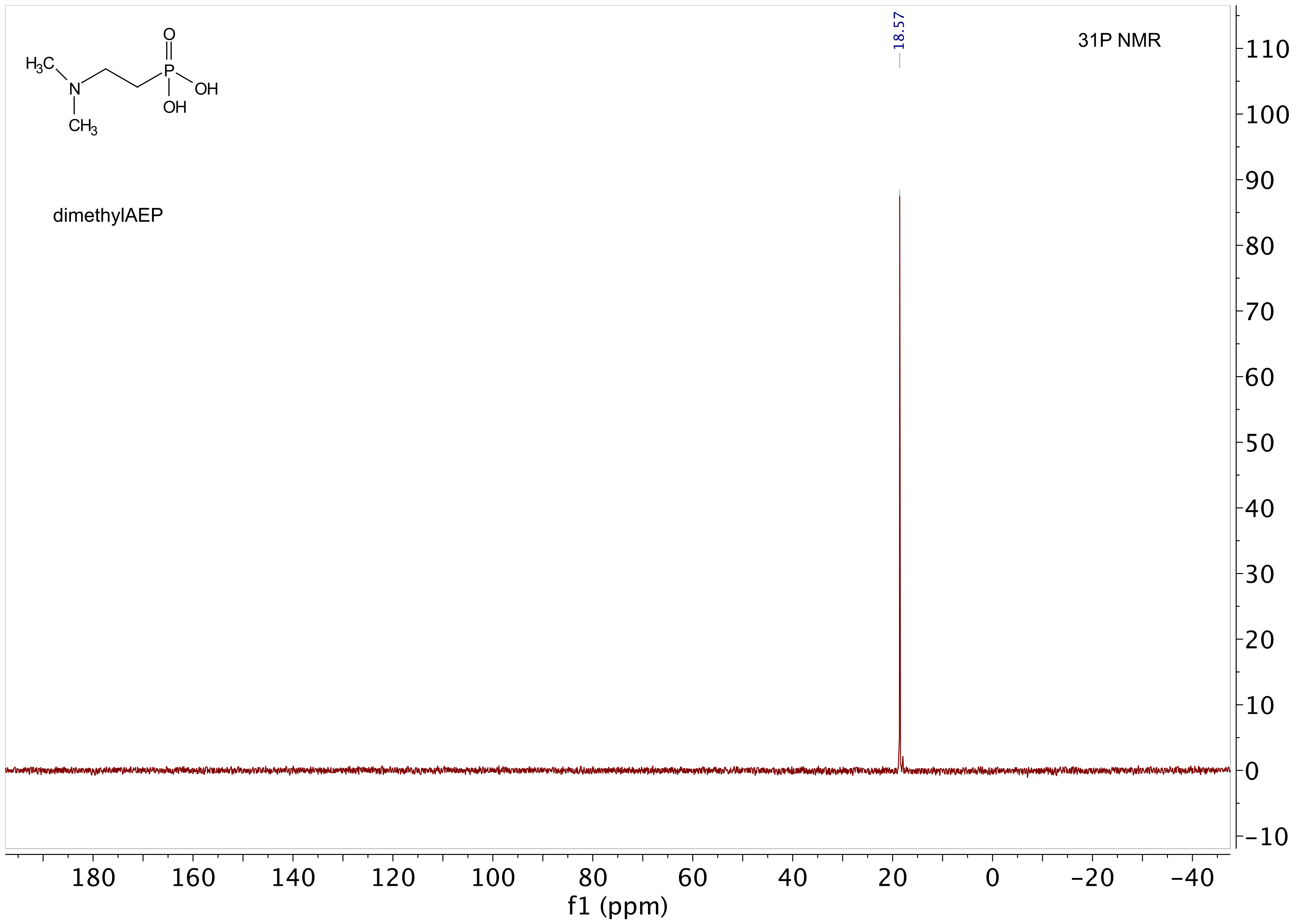

dimethylAEP
31P NMR
18.57
f1 (ppm)

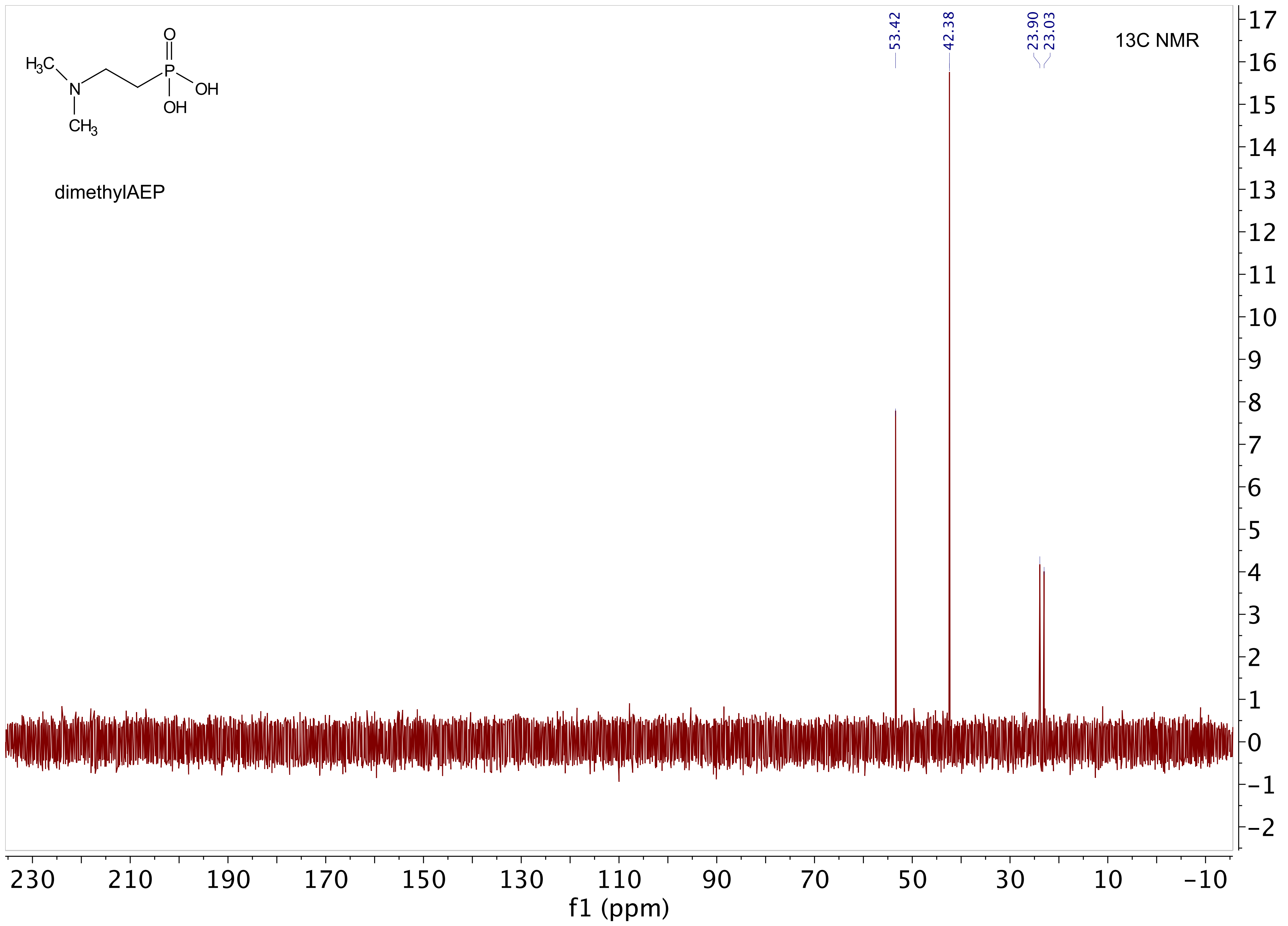

dimethylAEP
13C NMR
53.42
42.38
23.90
23.03
f1 (ppm)

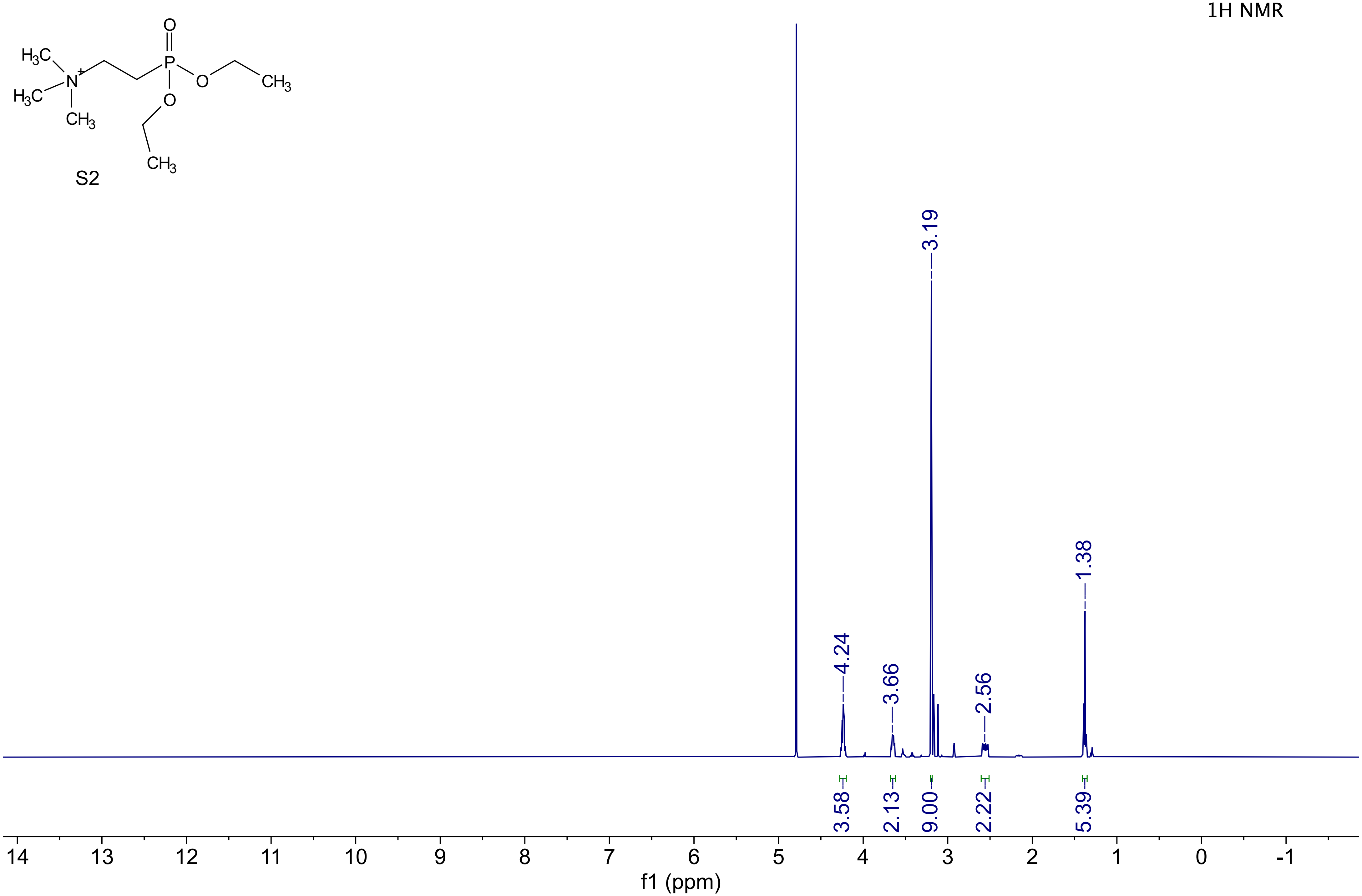

1H NMR
S2
4.24
3.66
3.19
2.56
1.38
3.58
2.13
9.00
2.22
5.39
14 13 12 11 10 9 8 7 6 5 4 3 2 1 0 -1
f1 (ppm)

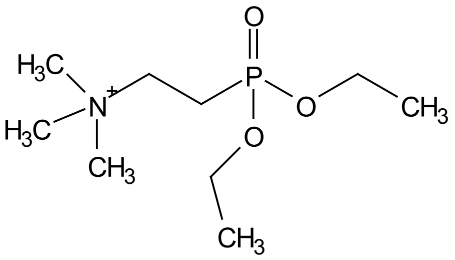

S2

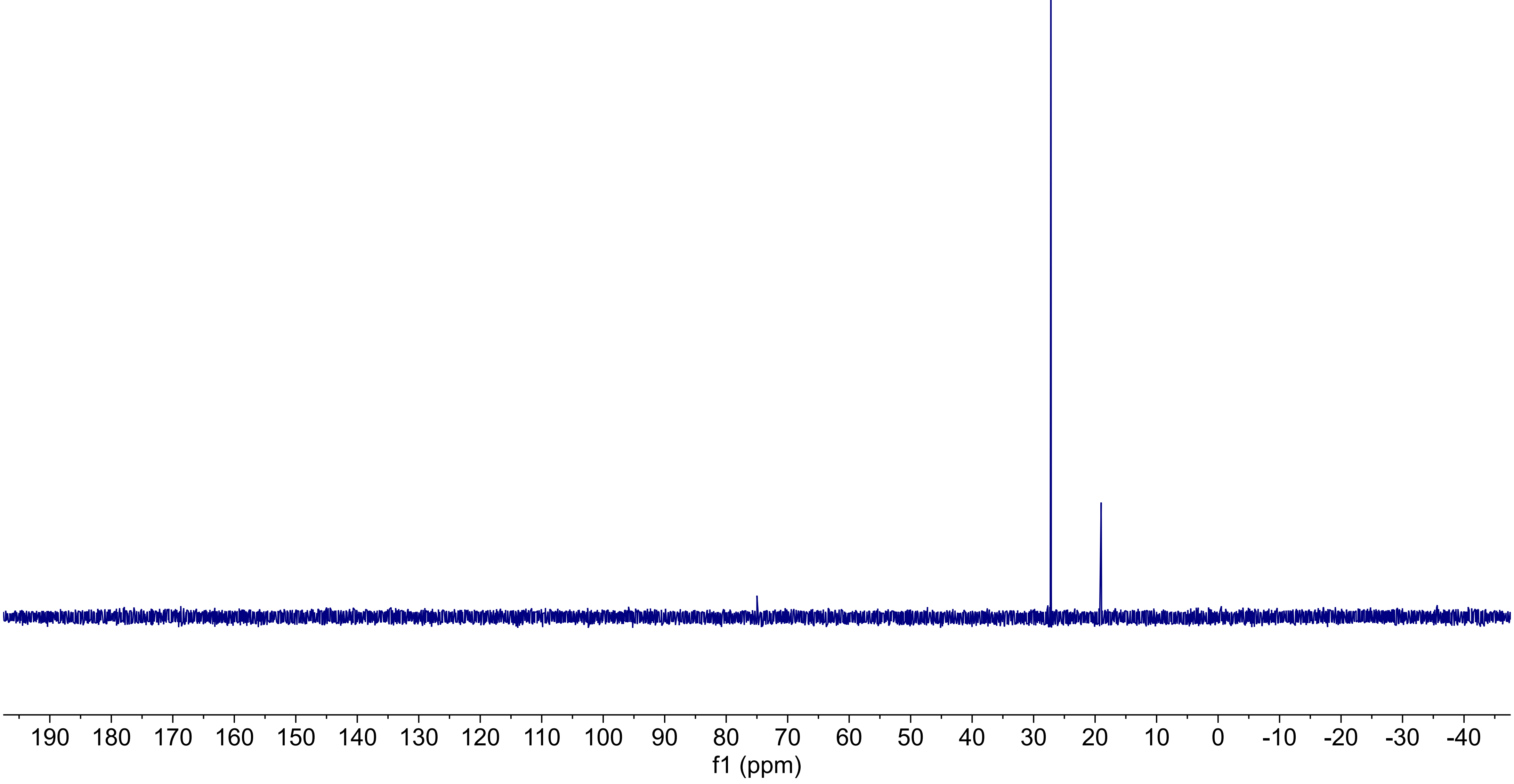
27.18
31P
NMR
190 180 170 160 150 140 130 120 110 100 90 80 70 60 50 40 30 20 10 0 -10 -20 -30 -40
f1 (ppm)

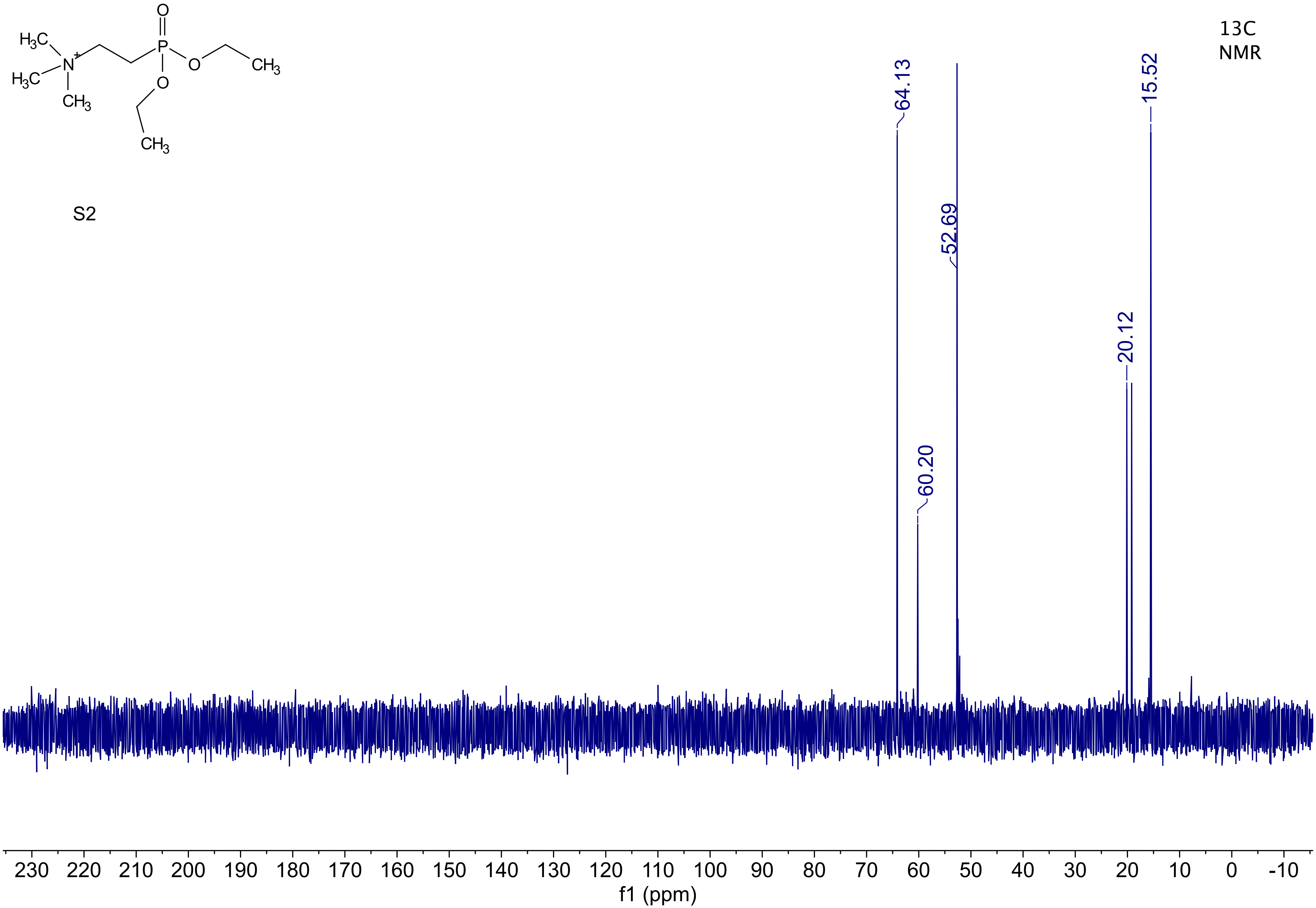

S2
13C
NMR
64.13
60.20
52.69
20.12
15.52
230 220 210 200 190 180 170 160 150 140 130 120 110 100 90 80 70 60 50 40 30 20 10 0 -10
f1 (ppm)

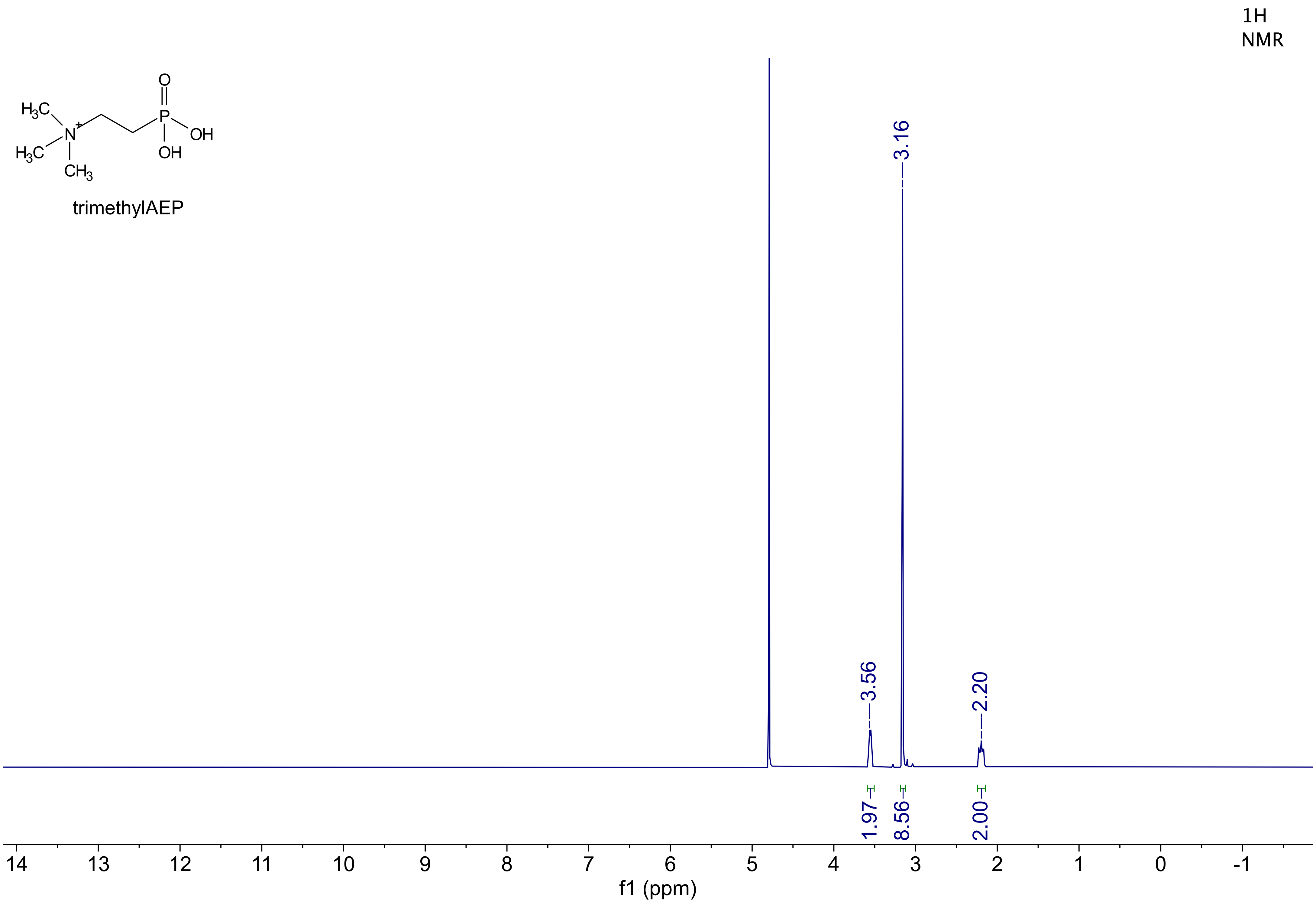
1H
NMR
trimethylAEP
3.56
3.16
2.20
1.97
8.56
2.00
14 13 12 11 10 9 8 7 6 5 4 3 2 1 0 -1
f1 (ppm)

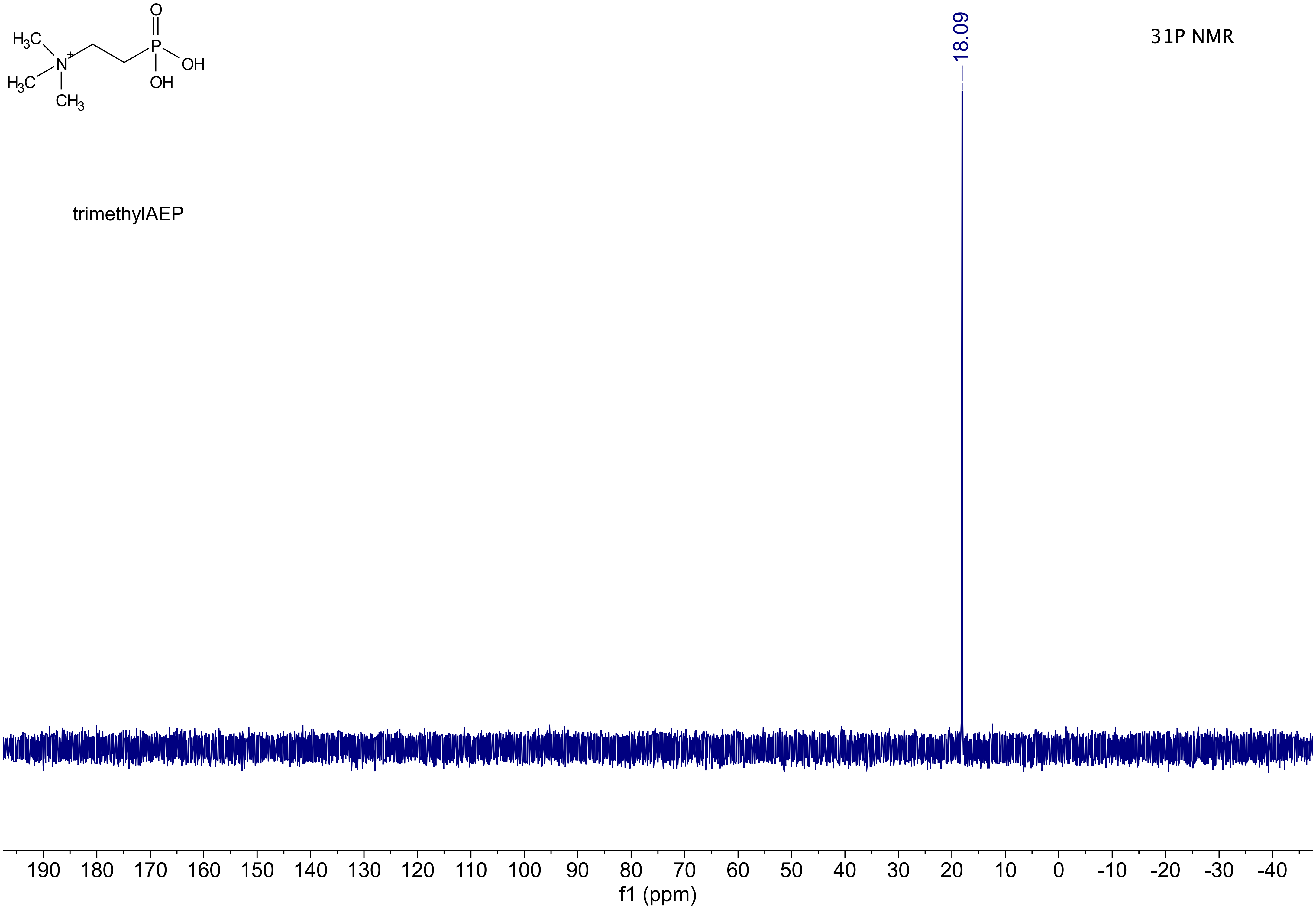
trimethylAEP
31P NMR
18.09
190 180 170 160 150 140 130 120 110 100 90 80 70 60 50 40 30 20 10 0 -10 -20 -30 -40
f1 (ppm)

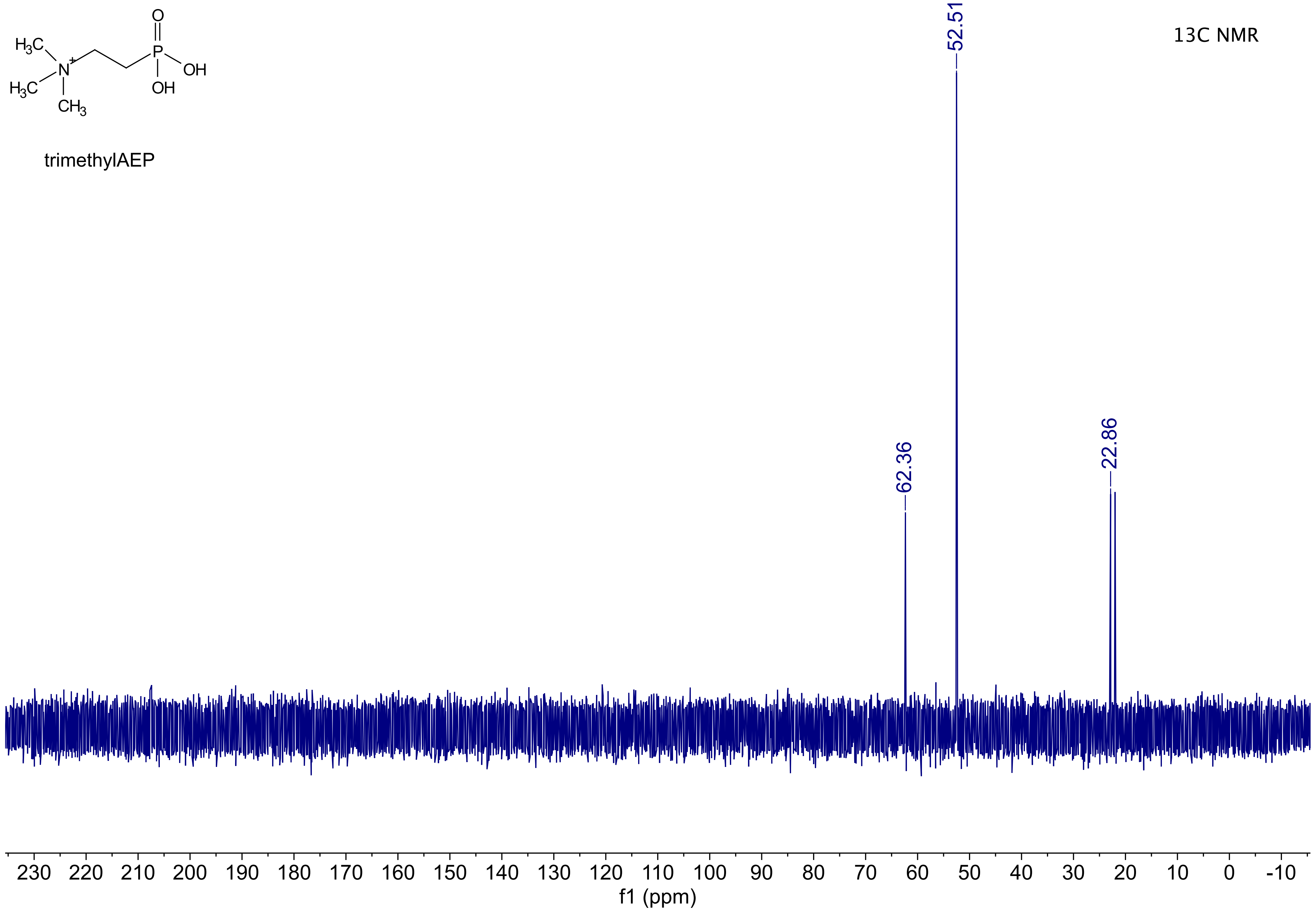
13C NMR
trimethylAEP
62.36
52.51
22.86
230 220 210 200 190 180 170 160 150 140 130 120 110 100 90 80 70 60 50 40 30 20 10 0 -10
f1 (ppm)

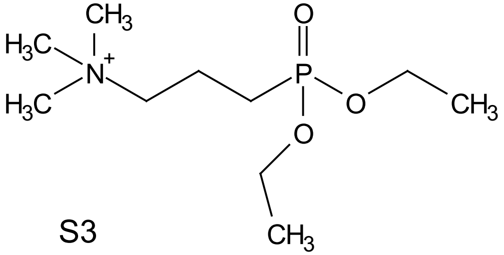

S3

1H NMR

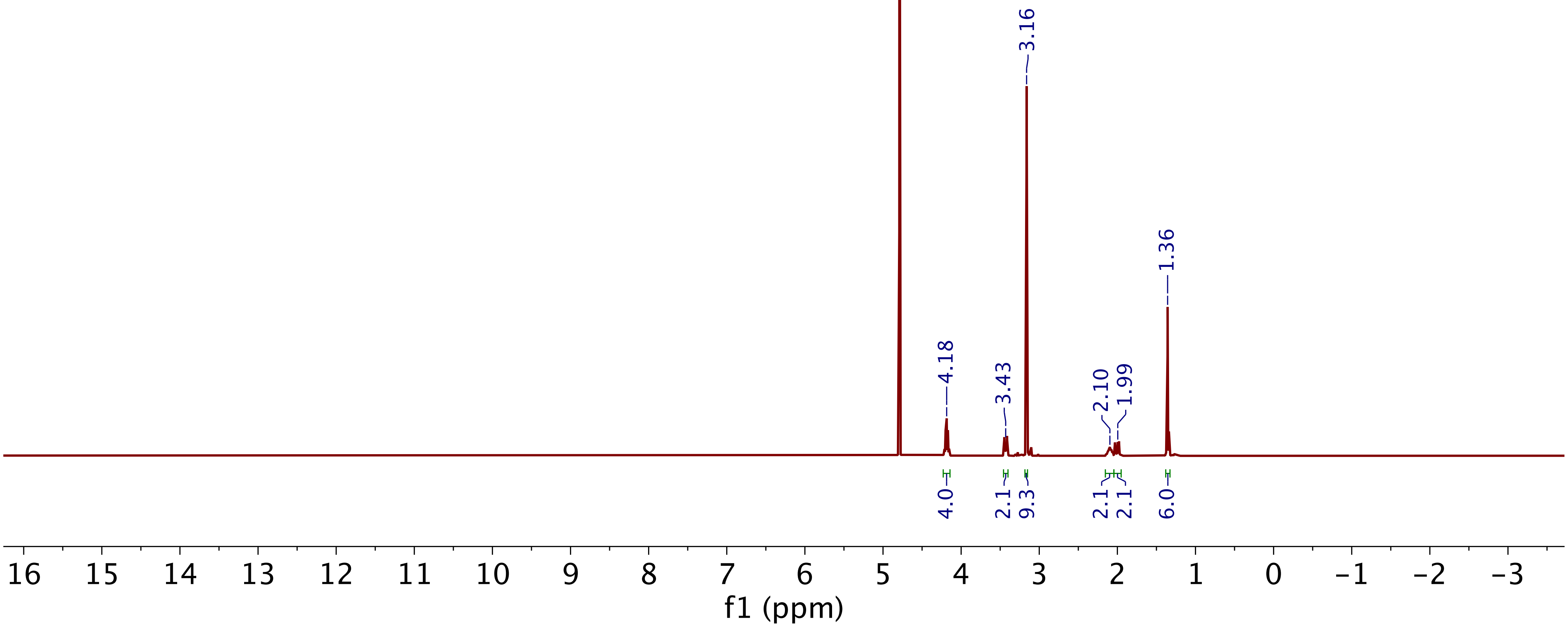
4.18
3.43
3.16
2.10
1.99
1.36
4.0
2.1
9.3
2.1
2.1
6.0
16 15 14 13 12 11 10 9 8 7 6 5 4 3 2 1 0 −1 −2 −3
f1 (ppm)

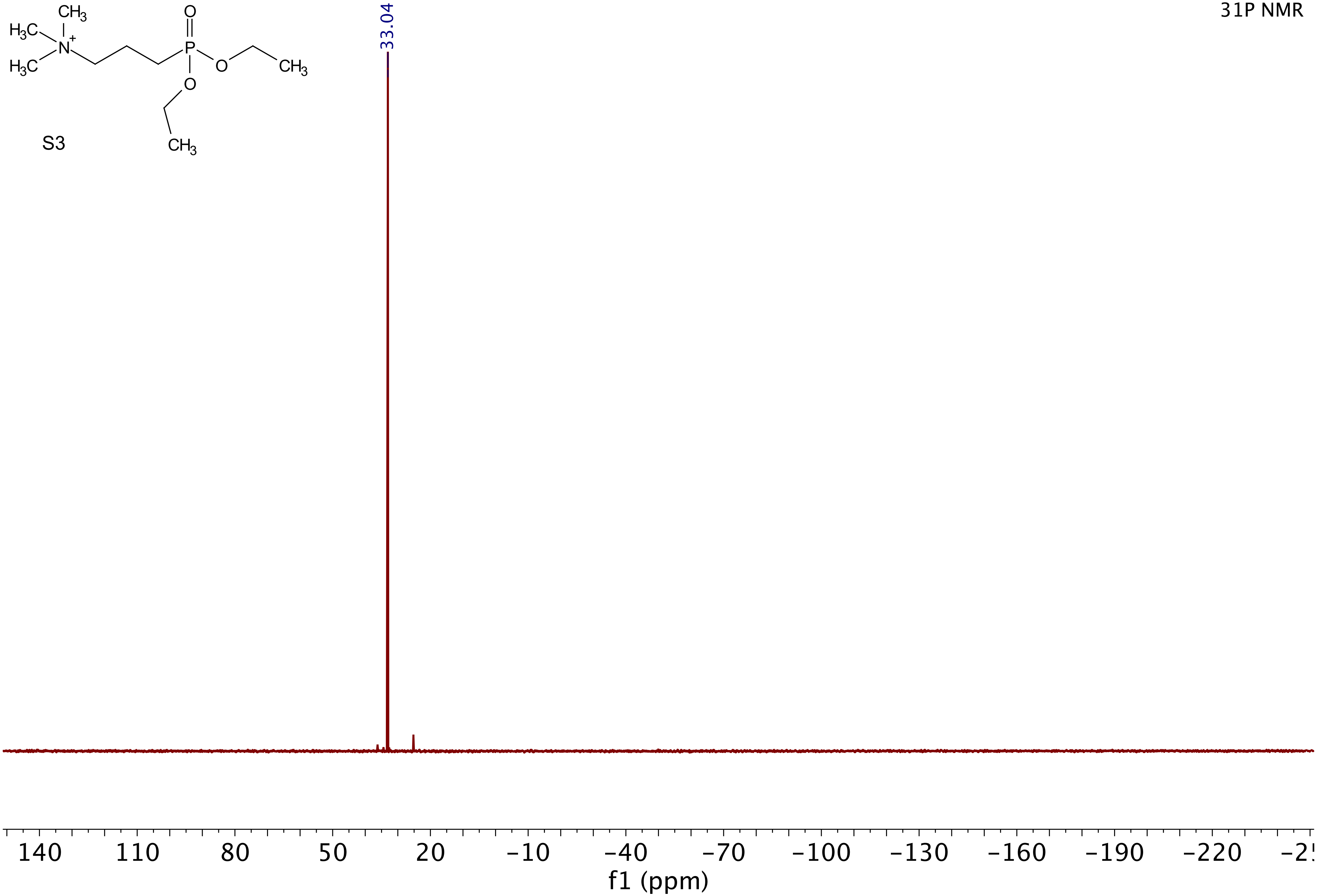
S3
31P NMR
33.04
140 110 80 50 20 −10 −40 −70 −100 −130 −160 −190 −220
f1 (ppm)

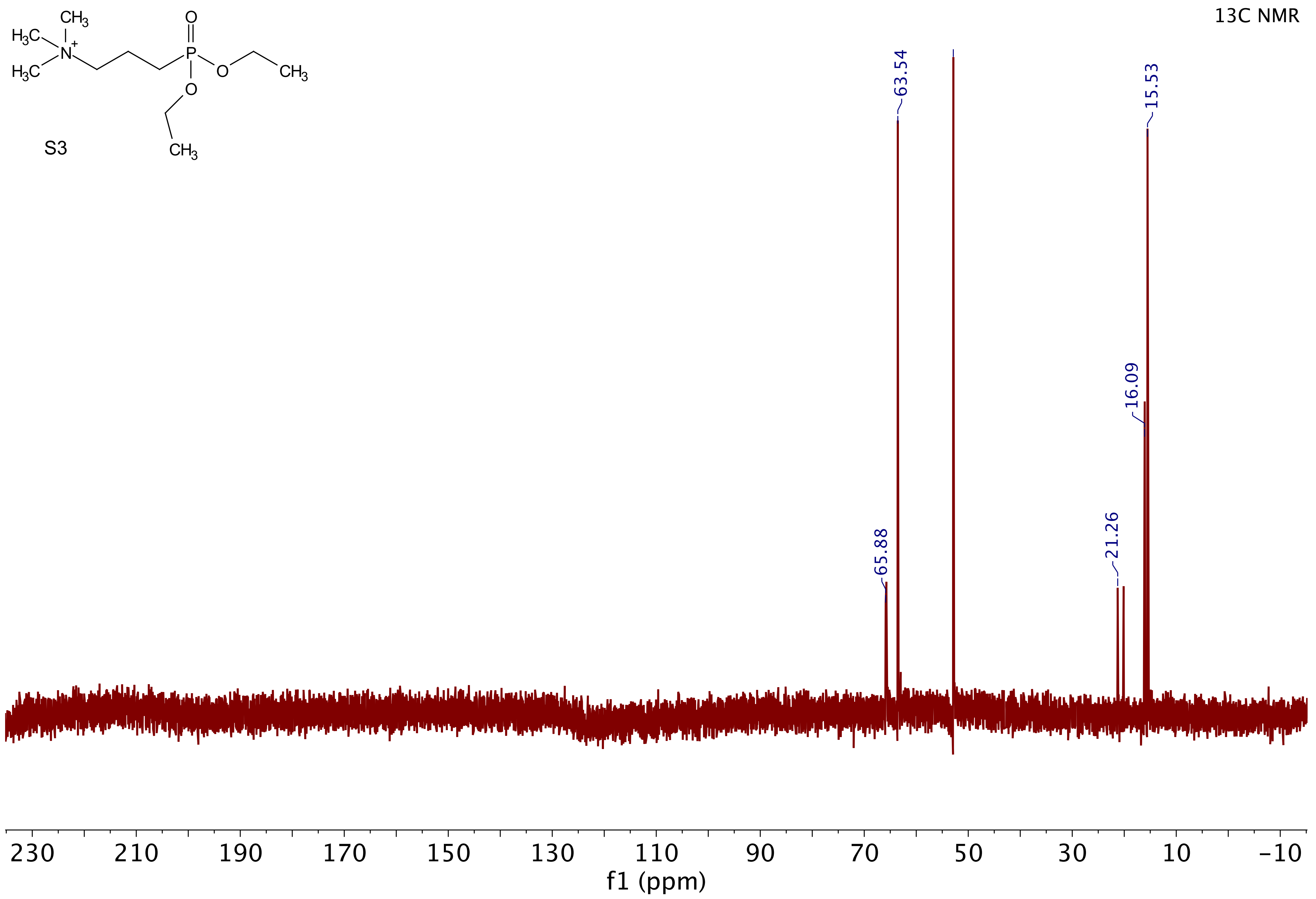

13C NMR
S3
65.88
63.54
21.26
16.09
15.53
230 210 190 170 150 130 110 90 70 50 30 10 −10
f1 (ppm)

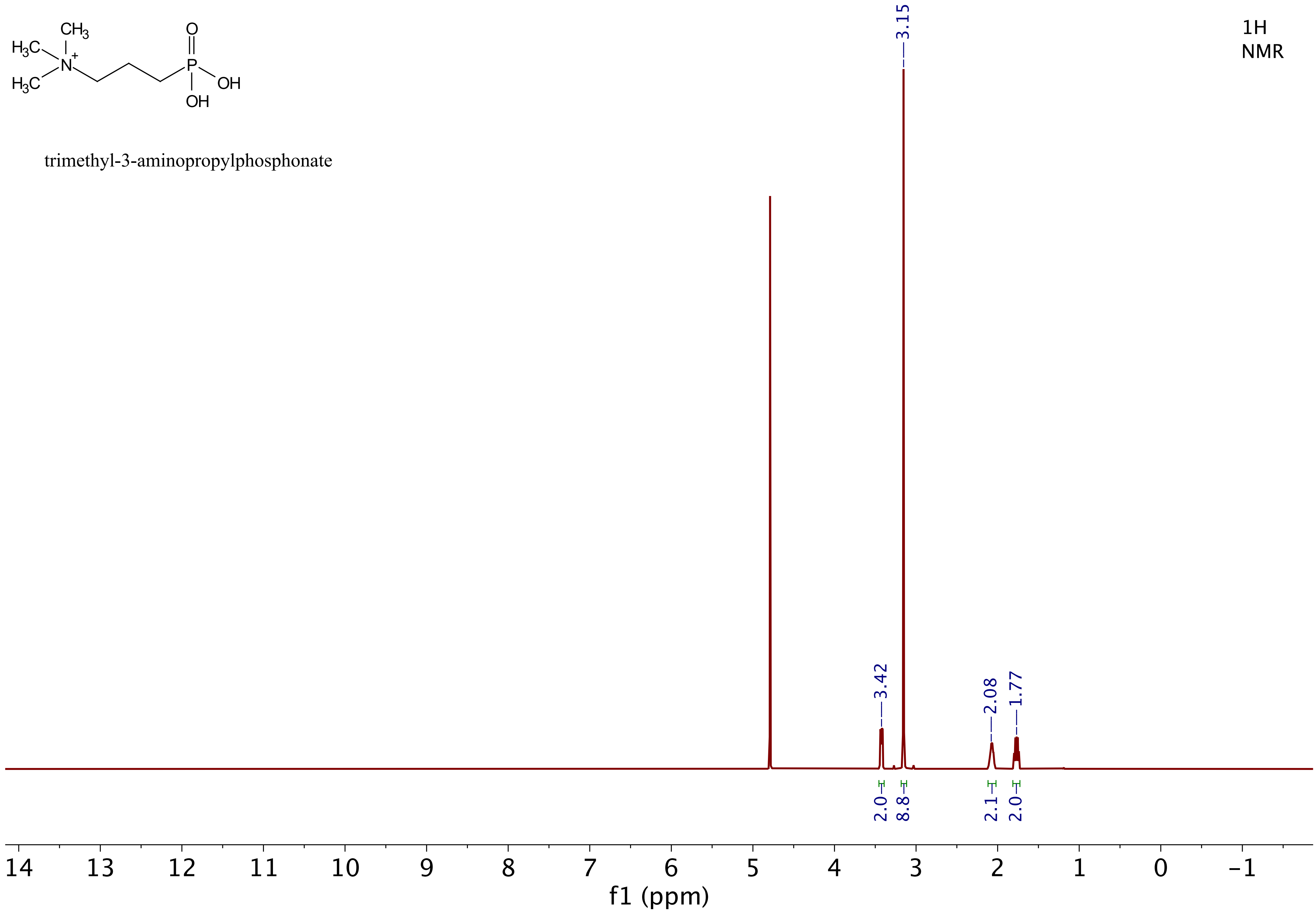

1H
NMR
trimethyl-3-aminopropylphosphonate
3.15
3.42
2.08
1.77
2.0
8.8
2.1
2.0
14
13
12
11
10
9
8
7
6
5
4
3
2
1
0
–1
f1 (ppm)

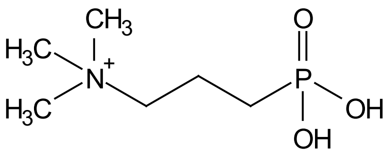

trimethyl-3-aminopropylphosphonate

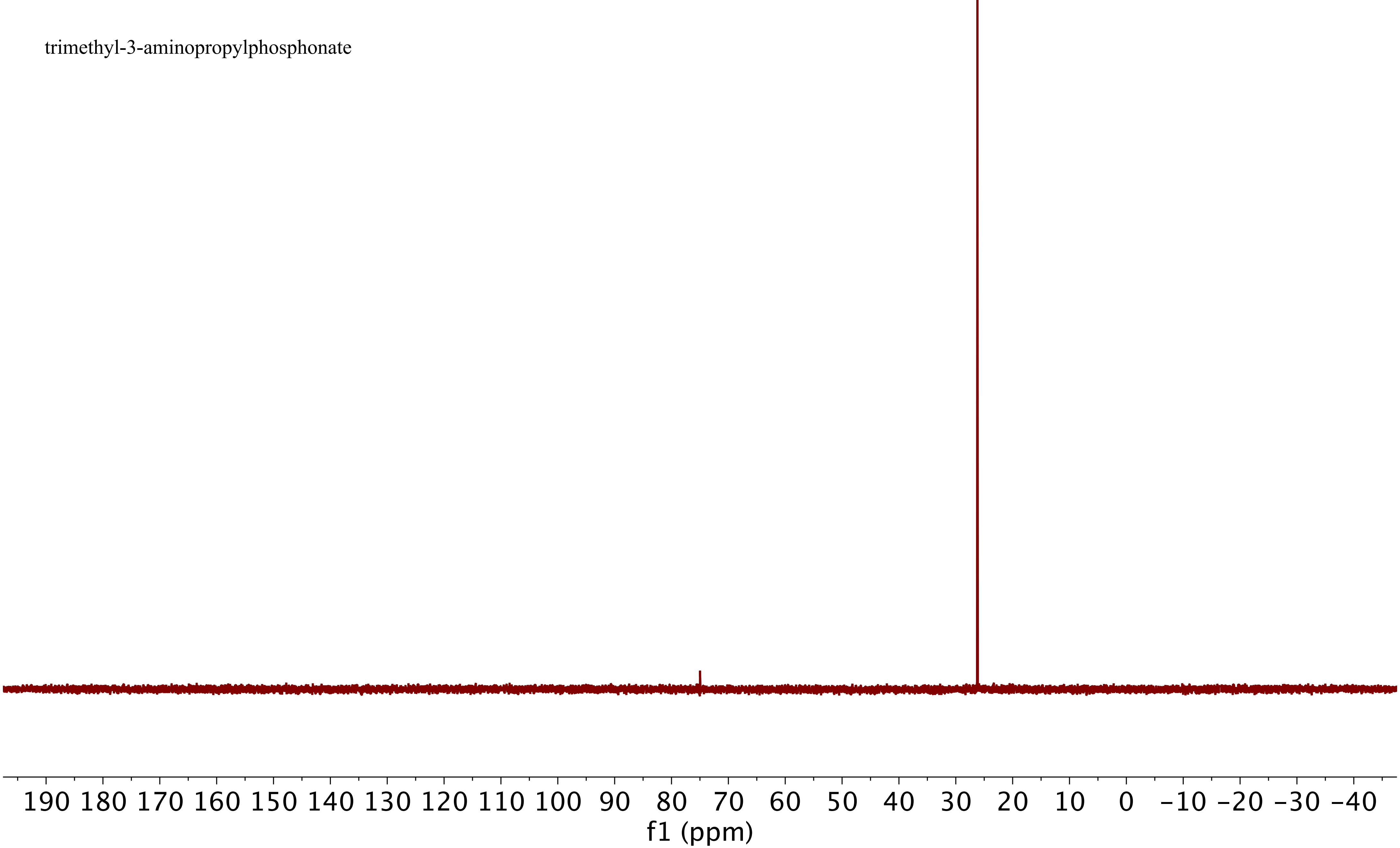

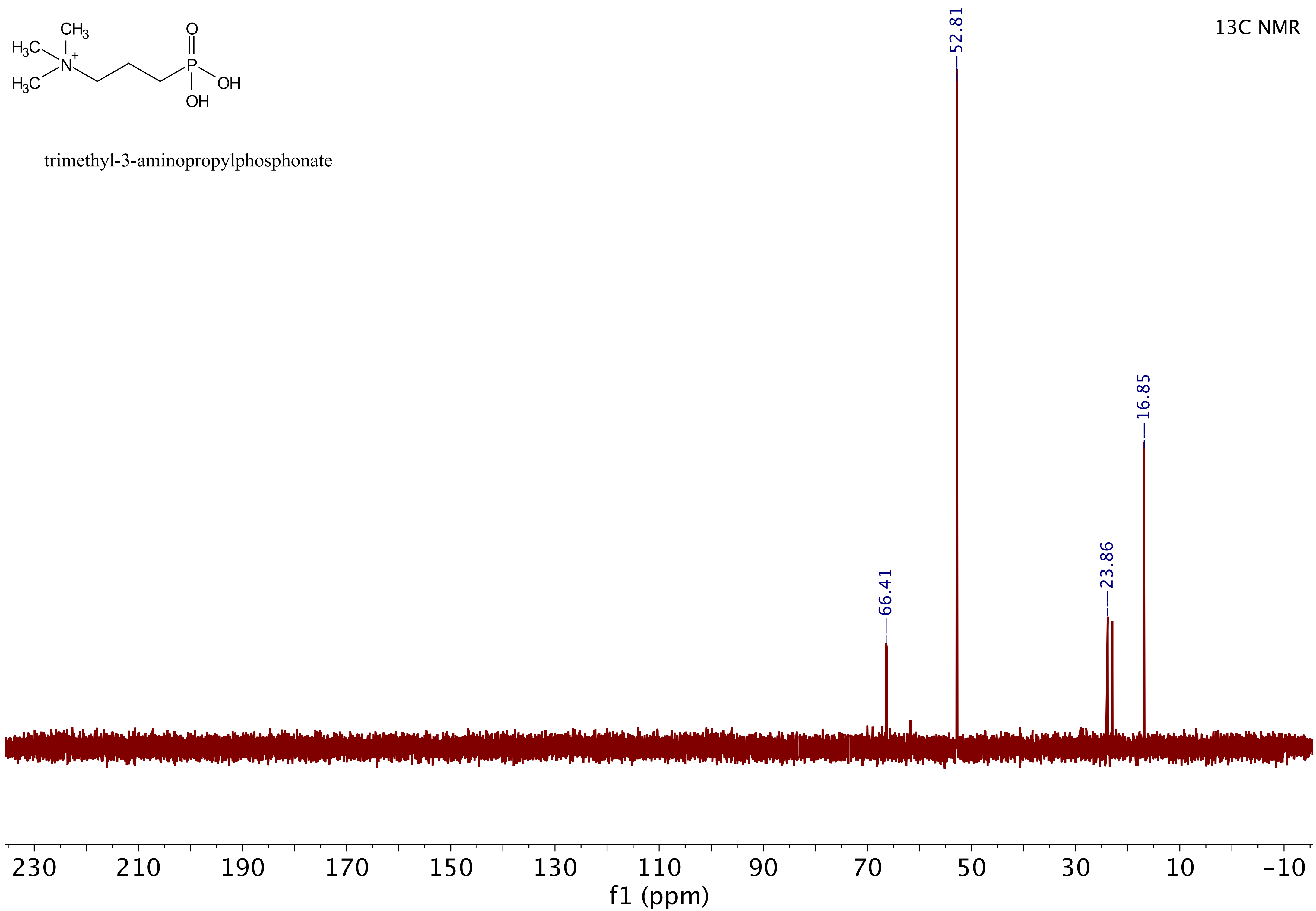

13C NMR
trimethyl-3-aminopropylphosphonate
66.41
52.81
23.86
16.85
230
210
190
170
150
130
110
90
70
50
30
10
−10
f1 (ppm)

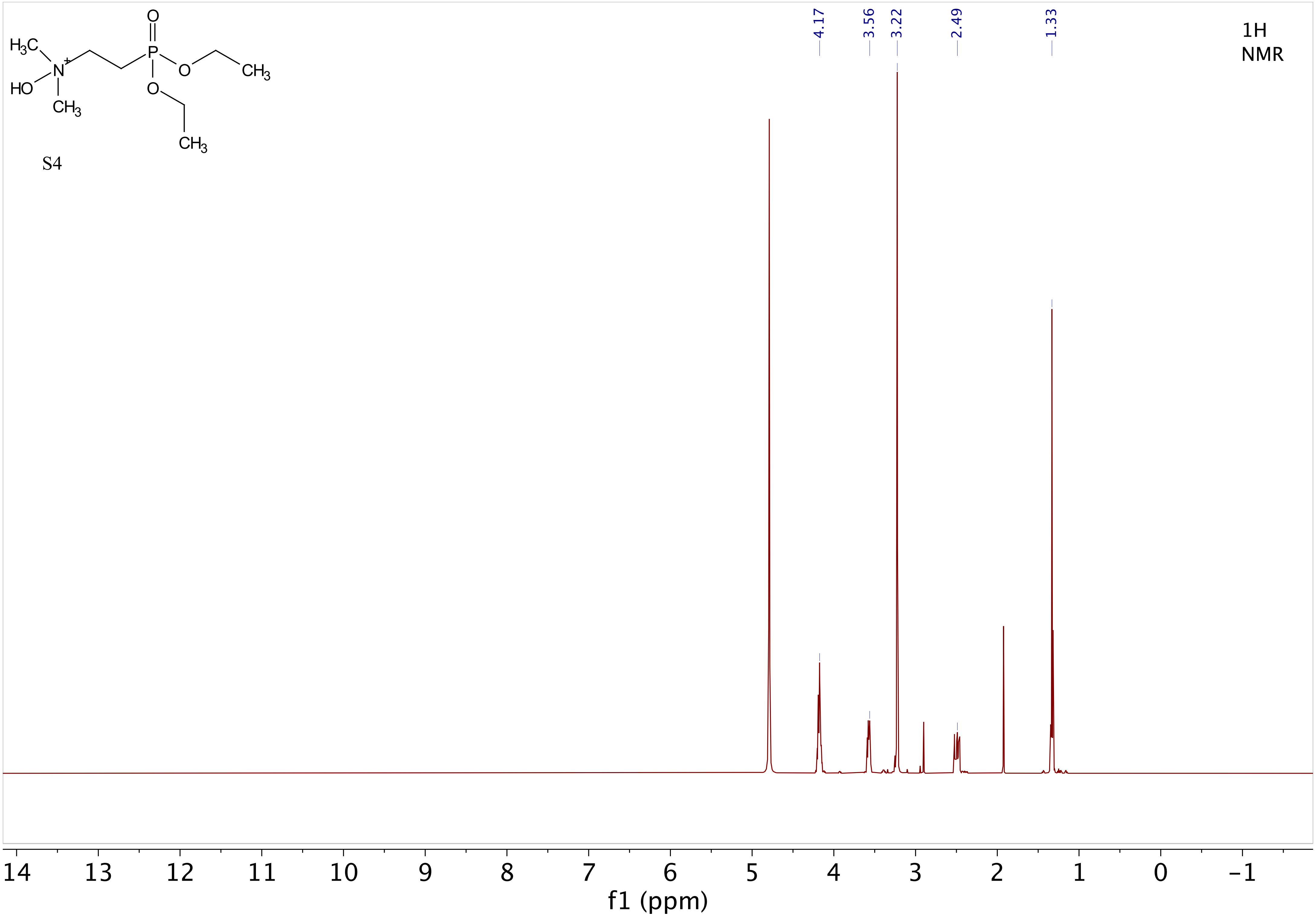

S4
4.17
3.56
3.22
2.49
1.33
1H
NMR
14 13 12 11 10 9 8 7 6 5 4 3 2 1 0 –1
f1 (ppm)

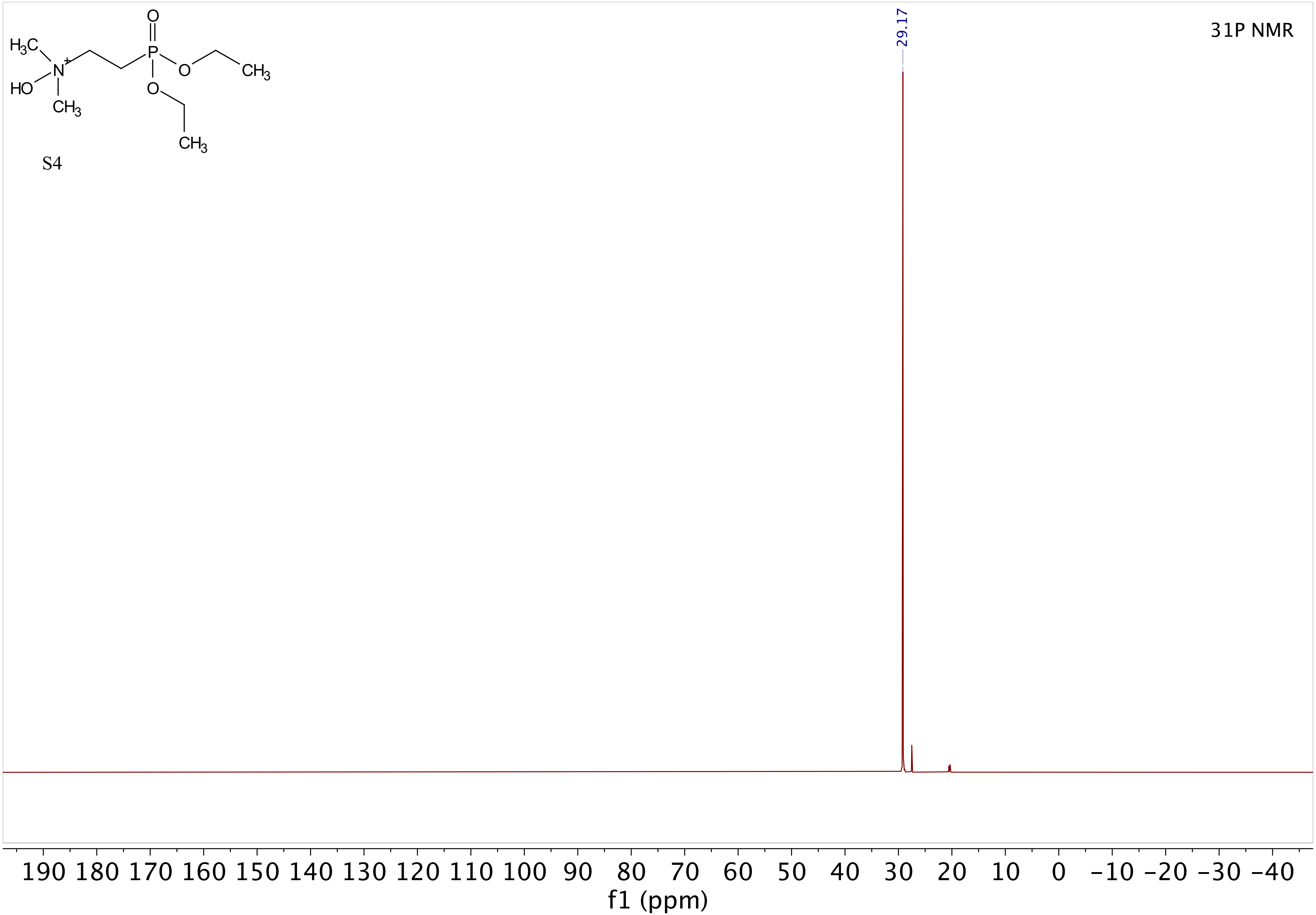

29.17
31P NMR
S4
190 180 170 160 150 140 130 120 110 100 90 80 70 60 50 40 30 20 10 0 −10 −20 −30 −40
f1 (ppm)

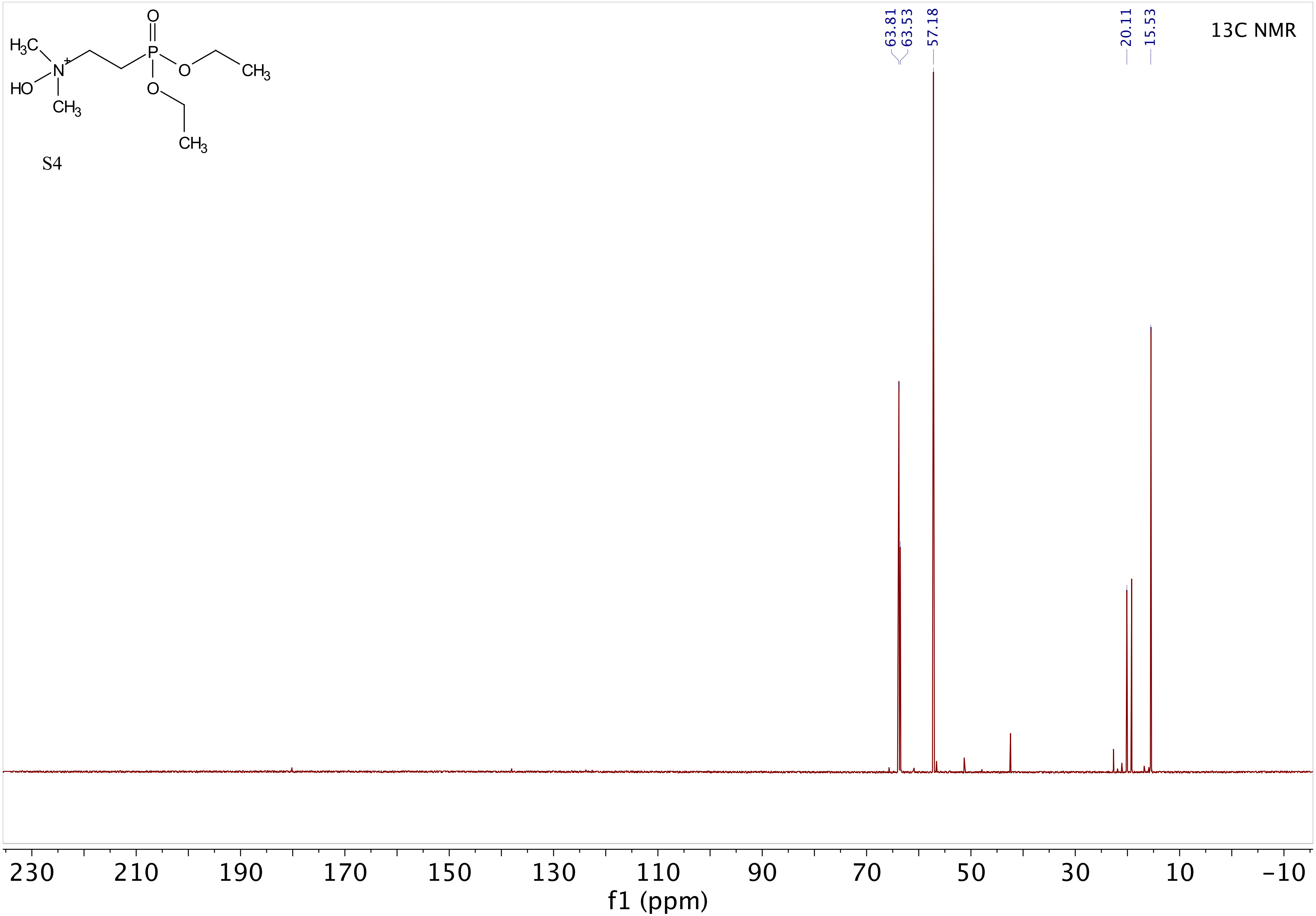

S4
13C NMR
63.81
63.53
57.18
20.11
15.53
230 210 190 170 150 130 110 90 70 50 30 10 −10
f1 (ppm)

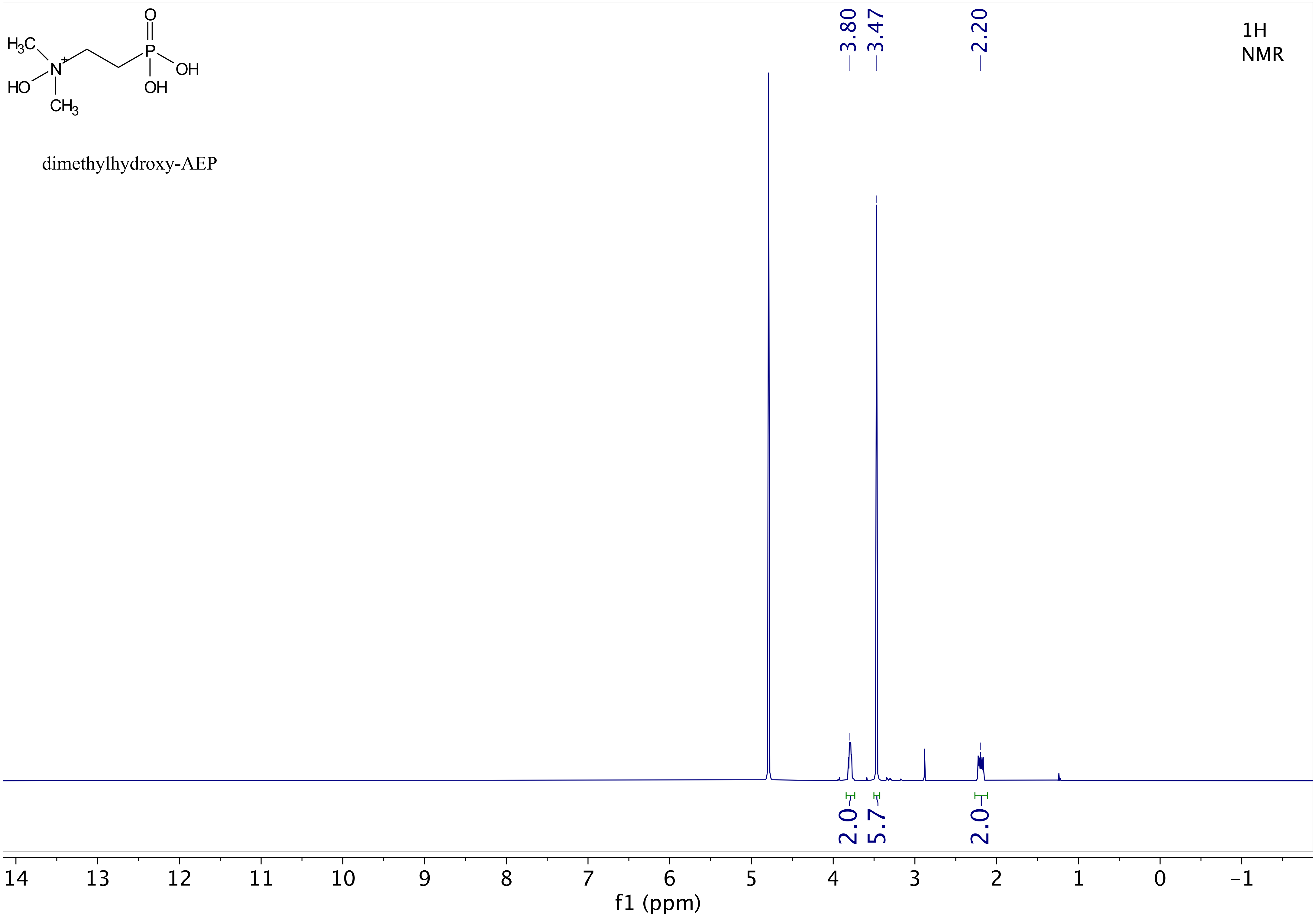
H3C
HO
N+
CH3
O
P
OH
OH
dimethylhydroxy-AEP
1H
NMR
3.80
3.47
2.20
2.0
5.7
2.0
14
13
12
11
10
9
8
7
6
5
4
3
2
1
0
−1
f1 (ppm)

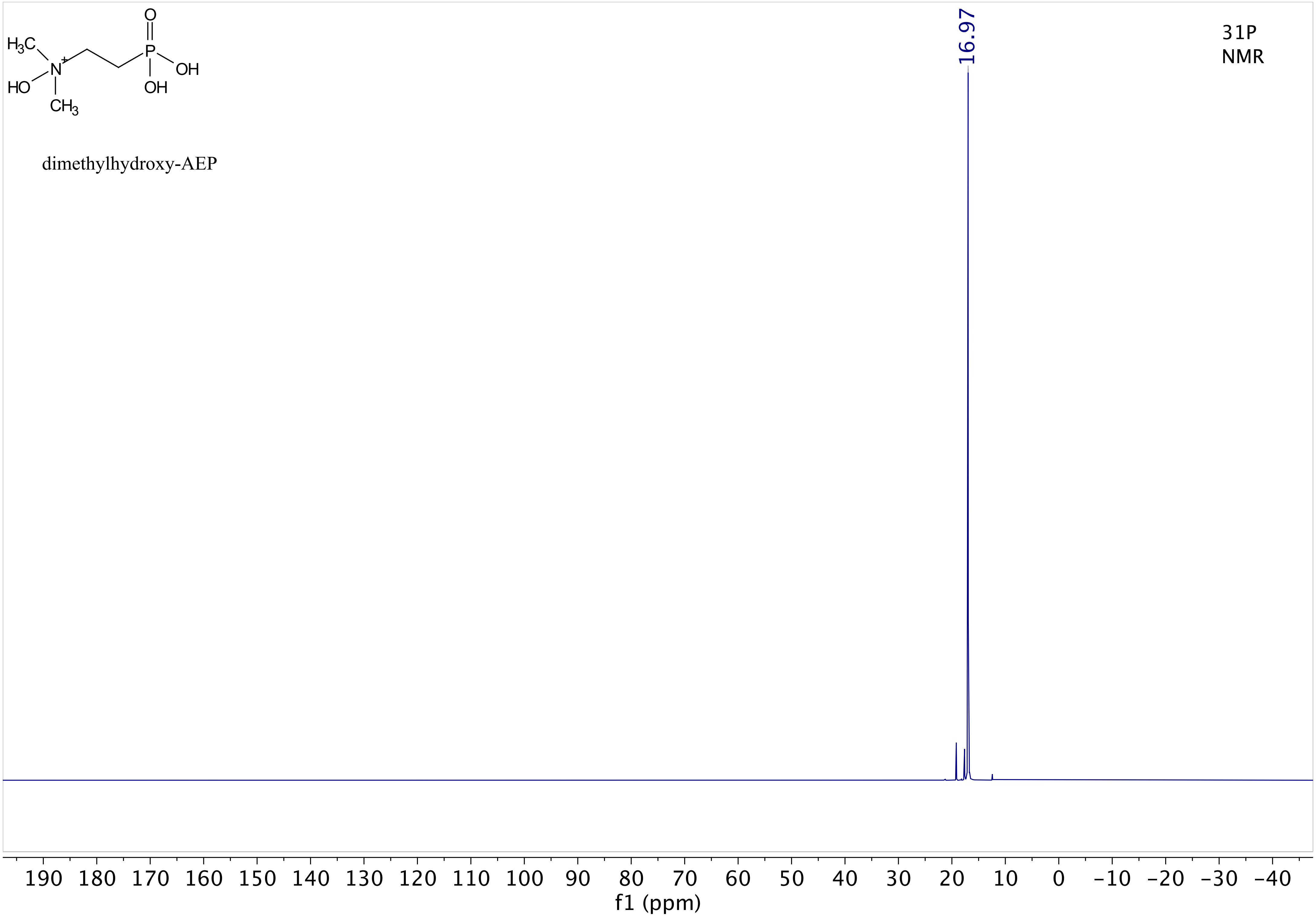
dimethylhydroxy-AEP
16.97
31P
NMR
190 180 170 160 150 140 130 120 110 100 90 80 70 60 50 40 30 20 10 0 −10 −20 −30 −40
f1 (ppm)

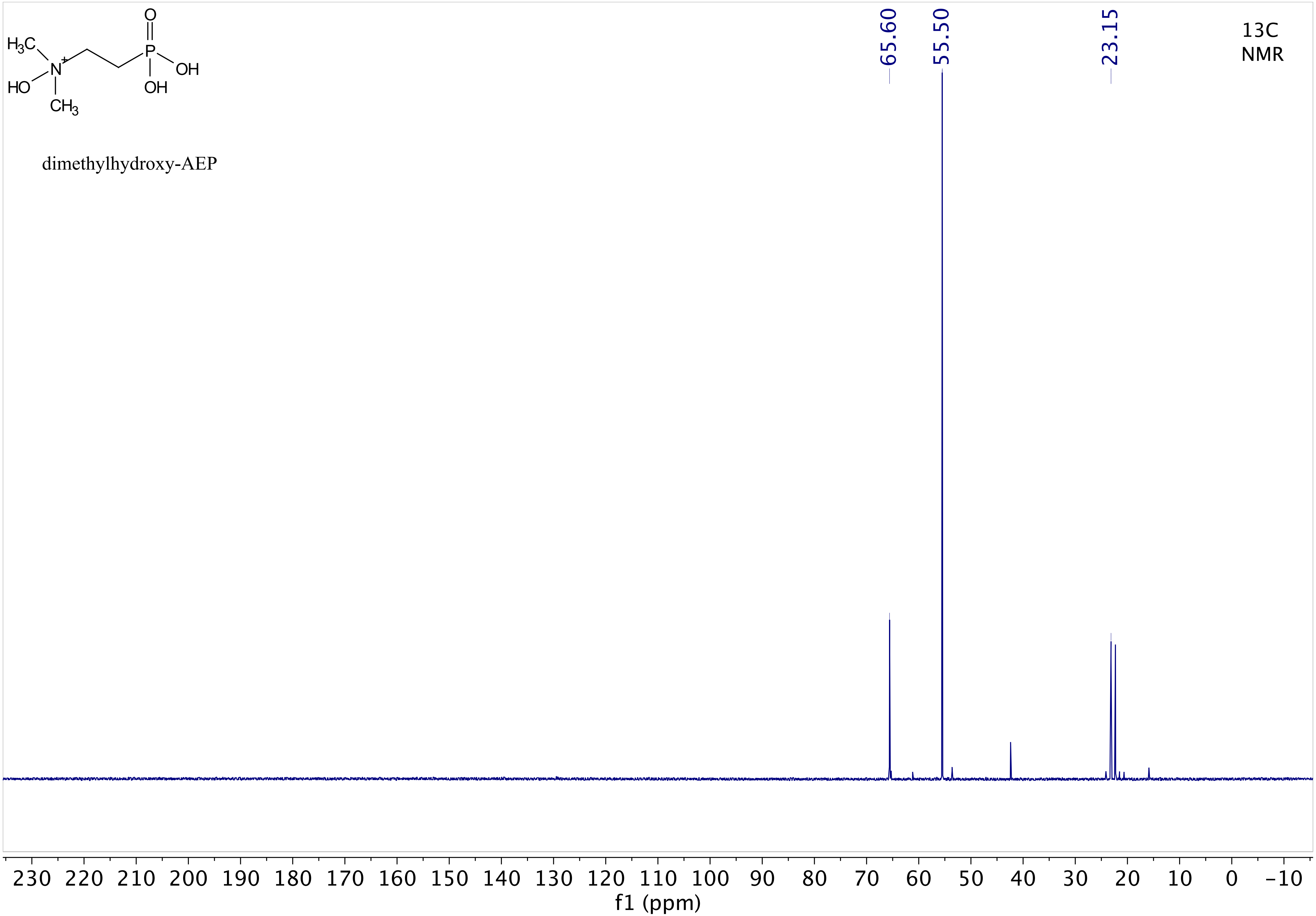

dimethylhydroxy-AEP
13C NMR
65.60
55.50
23.15
230 220 210 200 190 180 170 160 150 140 130 120 110 100 90 80 70 60 50 40 30 20 10 0 −10
f1 (ppm)

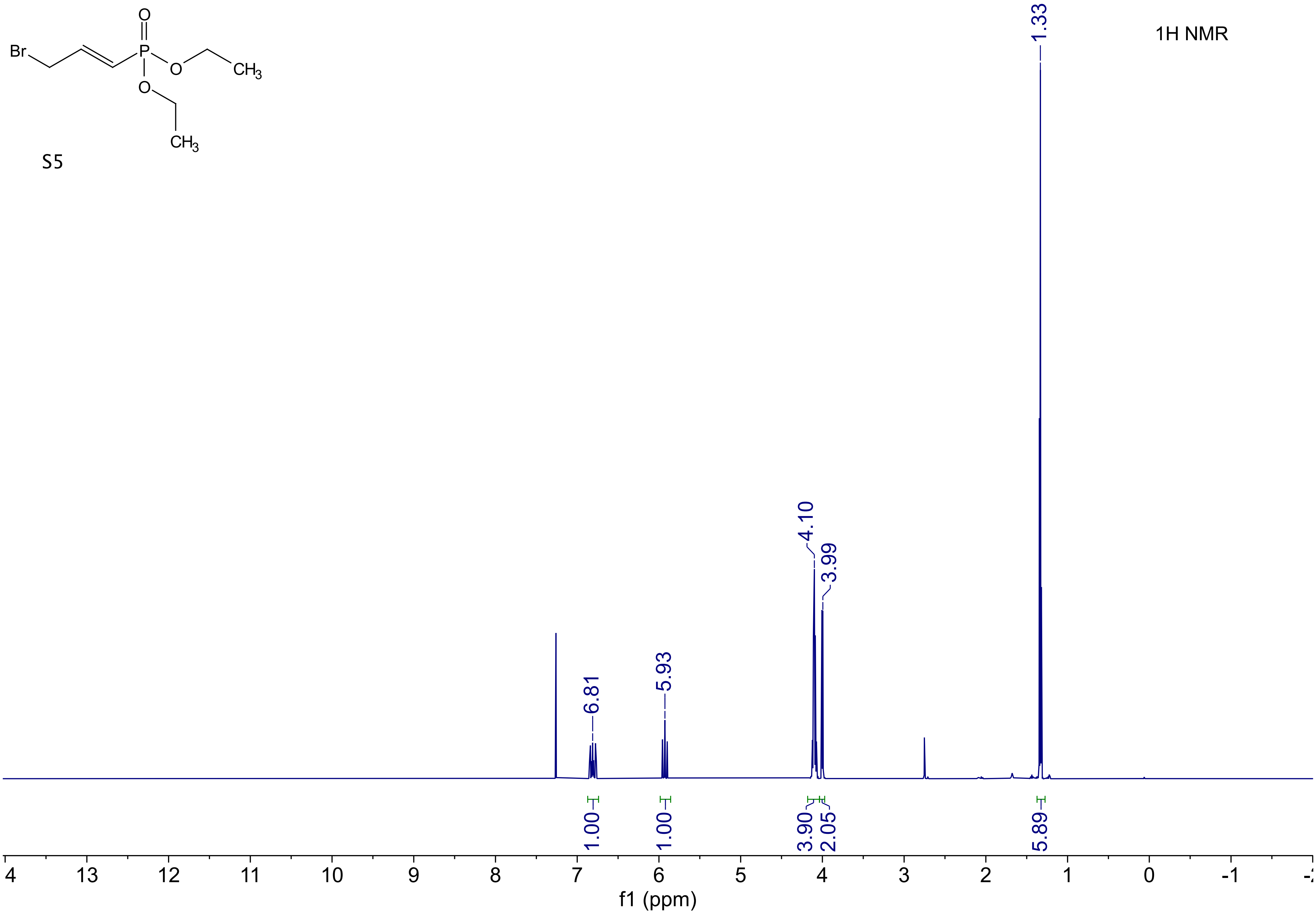

Br
O
P
O
CH3
O
CH3
S5
1H NMR
1.33
4.10
3.99
5.93
6.81
1.00
1.00
3.90
2.05
5.89
f1 (ppm)

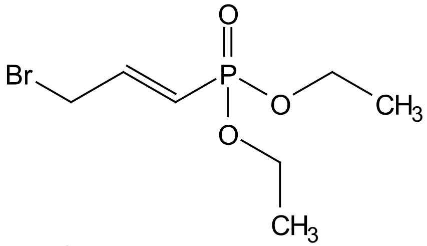

S5

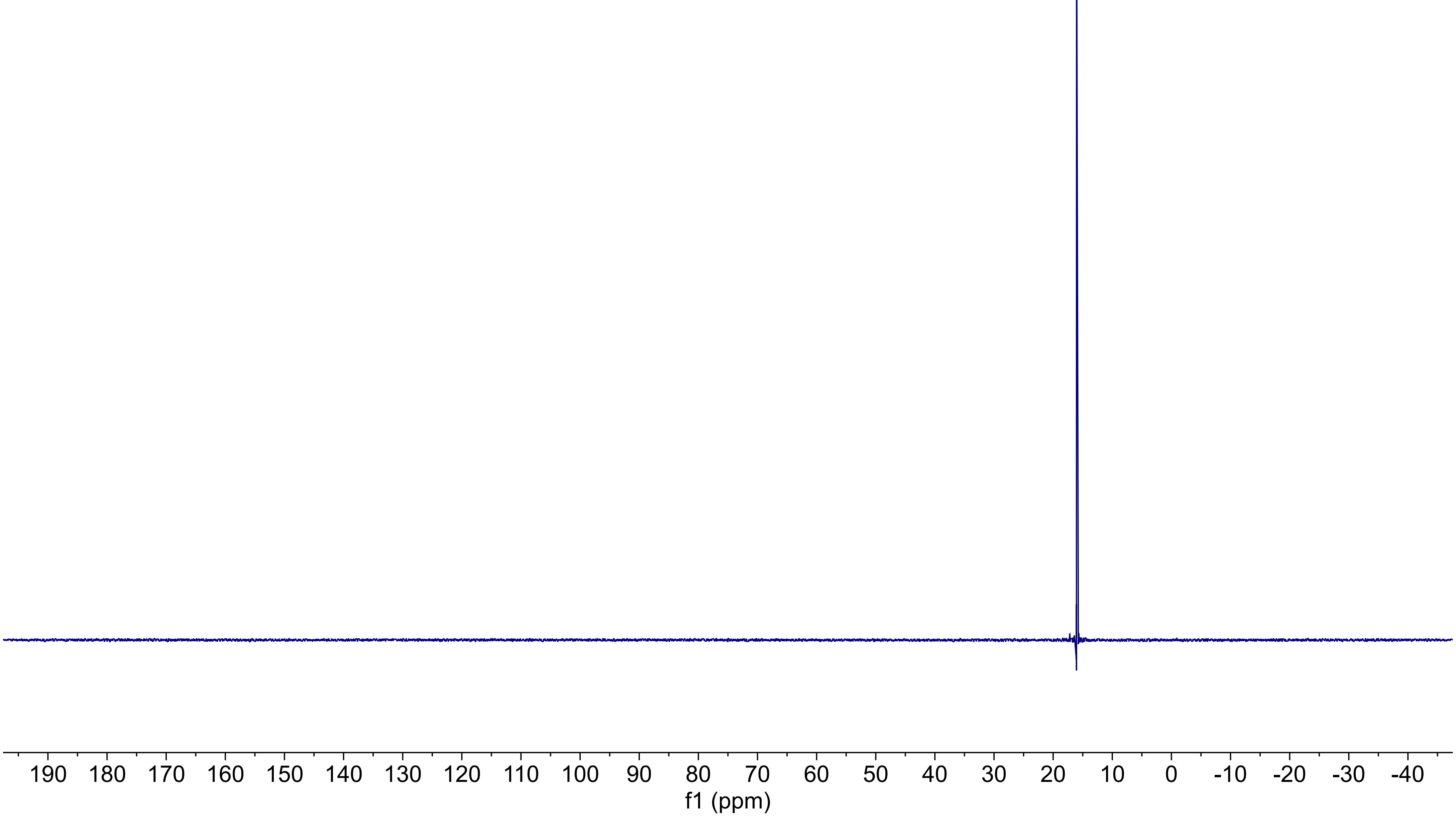

16.04
31P NMR
190
180
170
160
150
140
130
120
110
100
90
80
70
60
50
40
30
20
10
0
-10
-20
-30
-40
f1 (ppm)

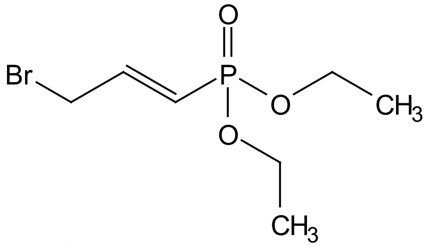

S5

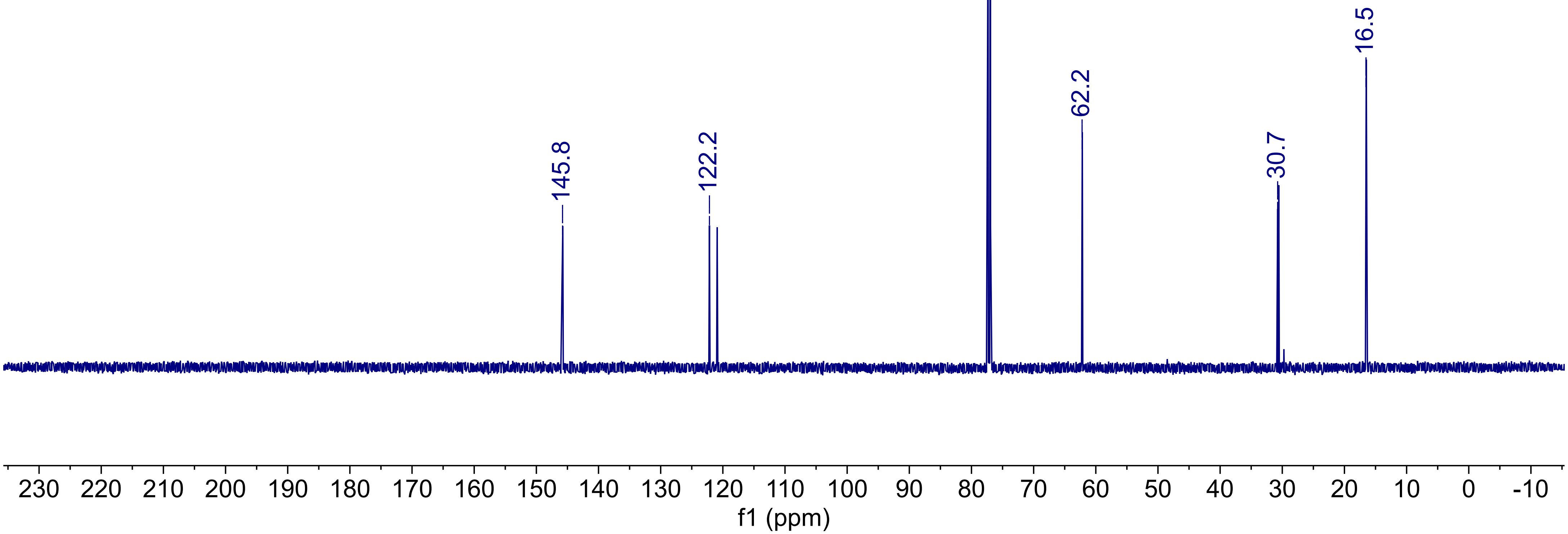
13C NMR
145.8
122.2
62.2
30.7
16.5
230 220 210 200 190 180 170 160 150 140 130 120 110 100 90 80 70 60 50 40 30 20 10 0 -10
f1 (ppm)

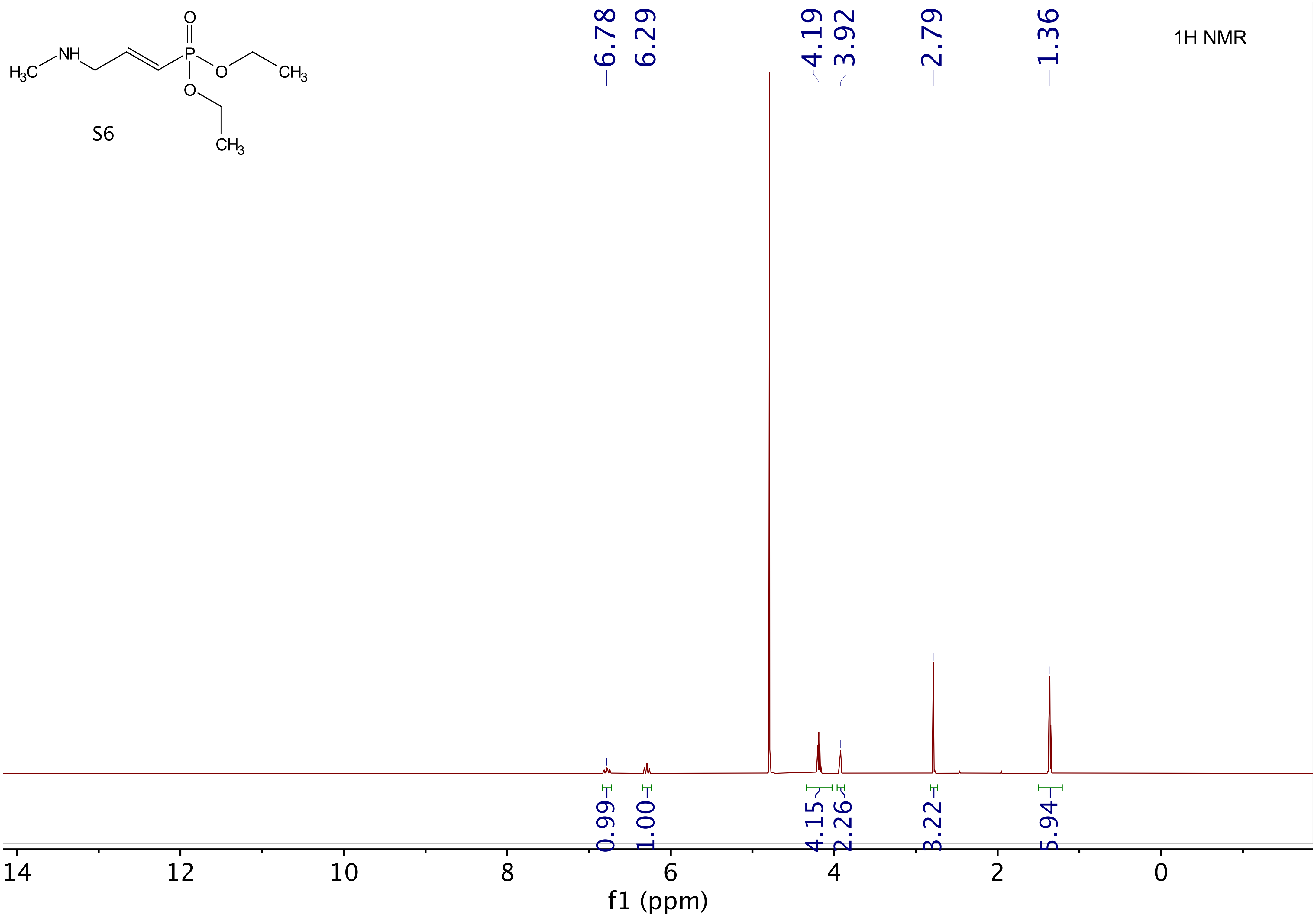
S6
1H NMR
6.78
6.29
4.19
3.92
2.79
1.36
0.99
1.00
4.15
2.26
3.22
5.94
14
12
10
8
6
4
2
0
f1 (ppm)

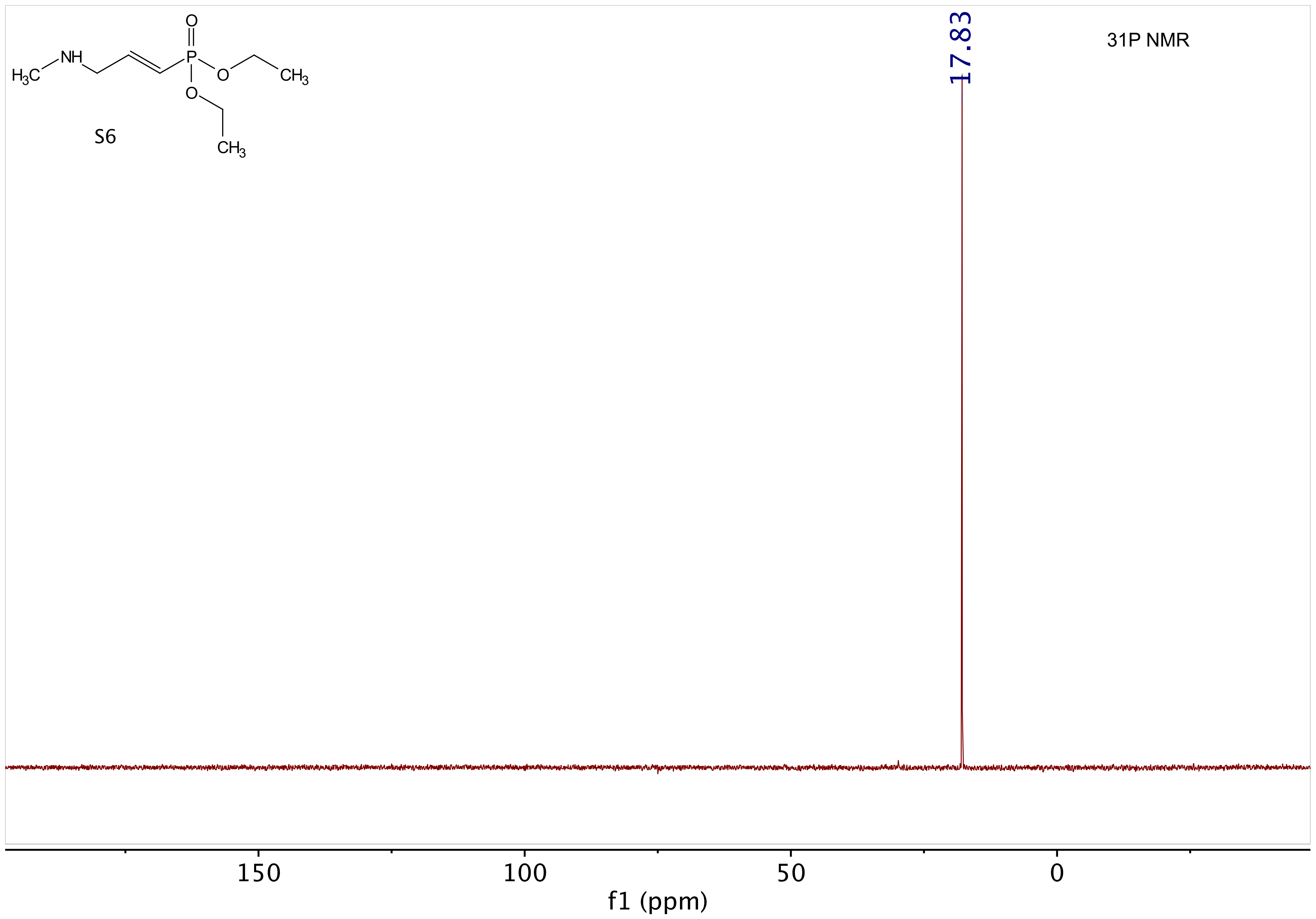

S6
17.83
31P NMR
150
100
50
0
f1 (ppm)

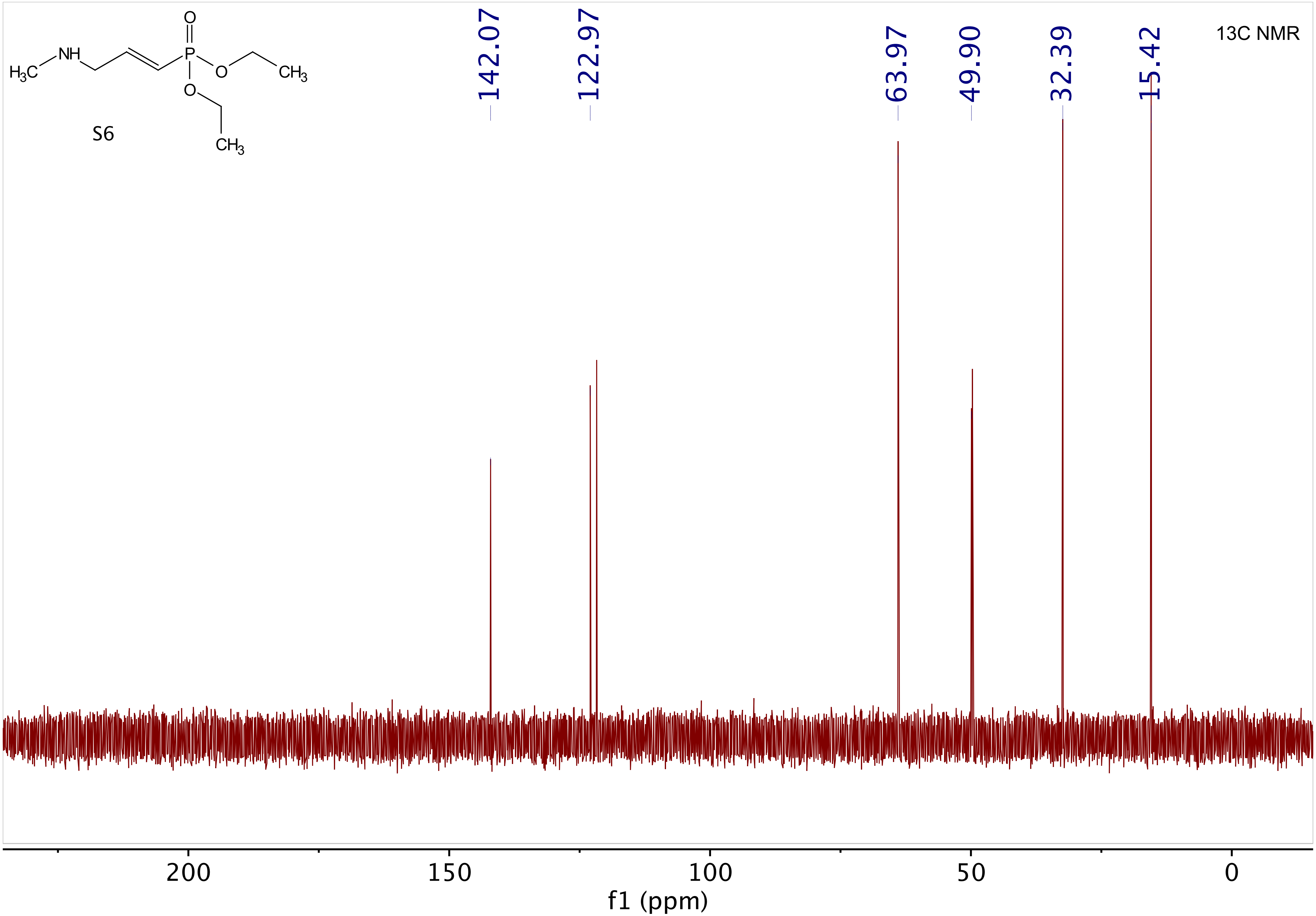

S6
13C NMR
142.07
122.97
63.97
49.90
32.39
15.42
200
150
100
50
0
f1 (ppm)

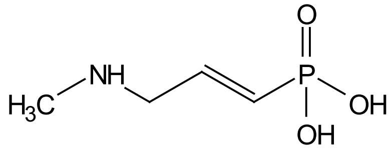

methyldehydrofosmidomycin

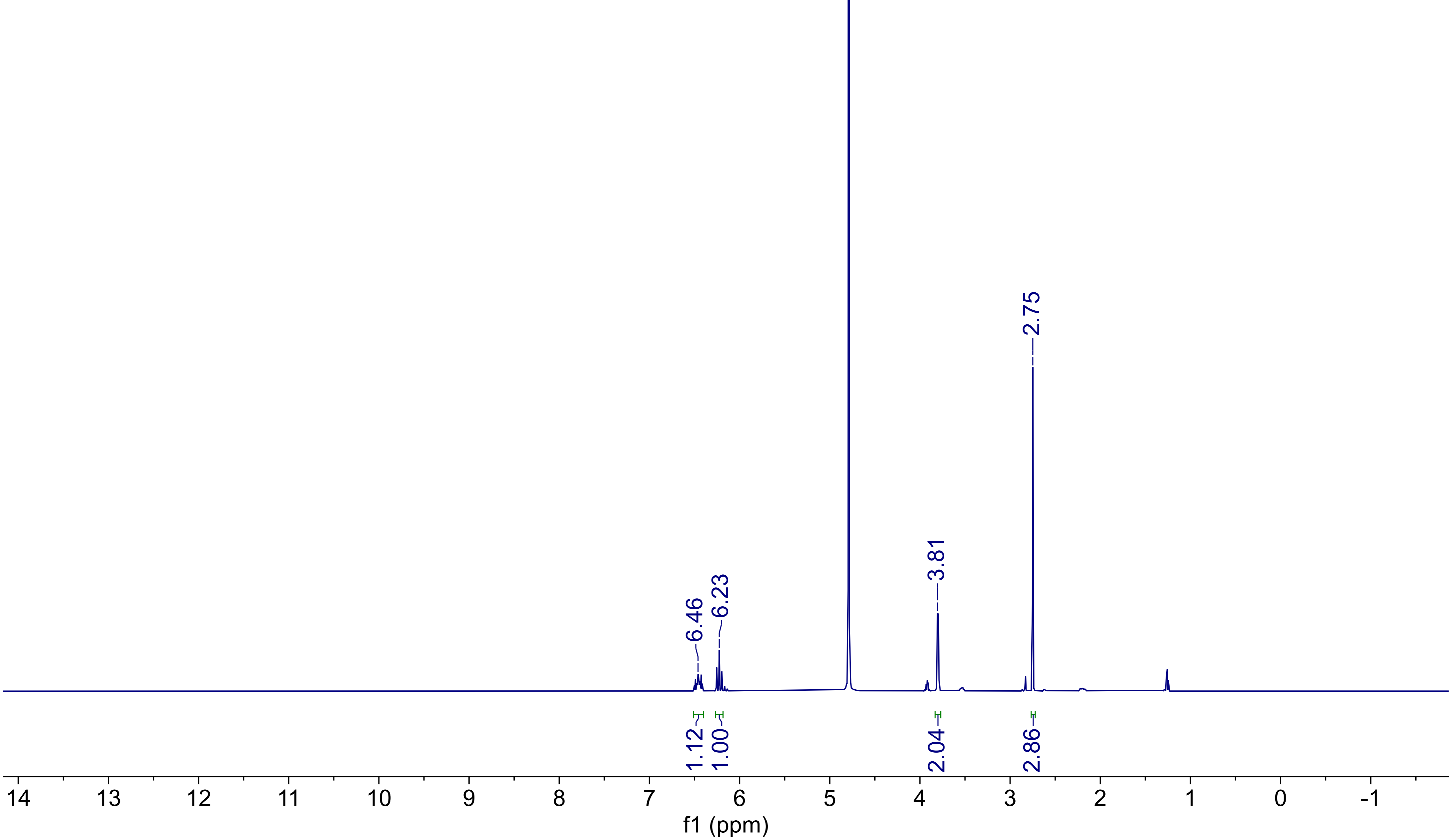

31P NMR

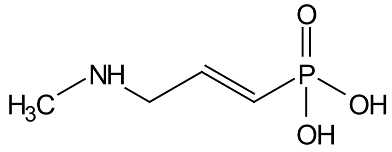

methyldehydrofosmidomycin

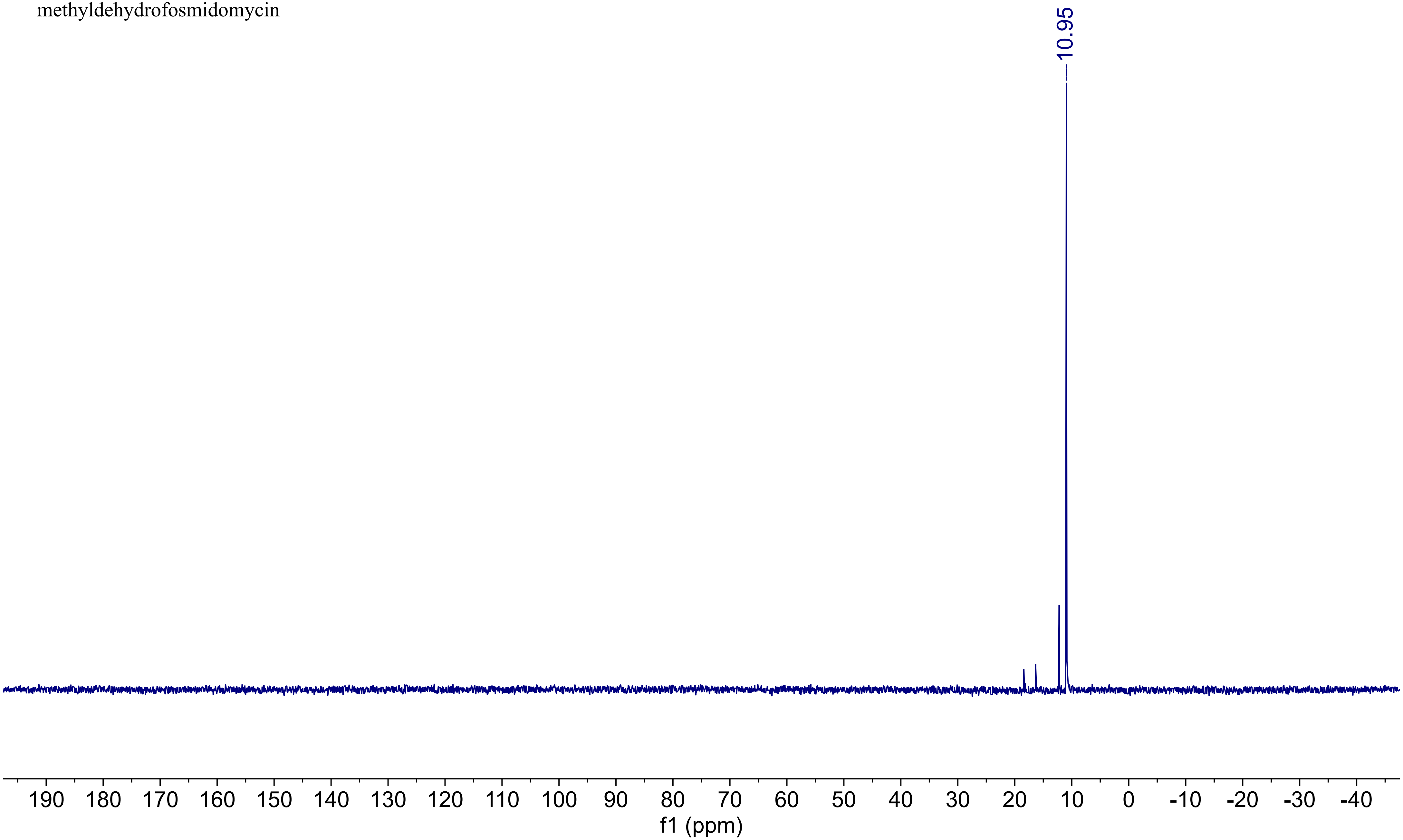

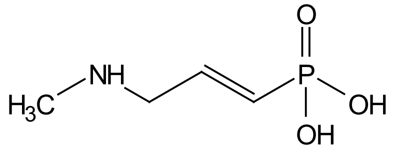

methyldehydrofosmidomycin

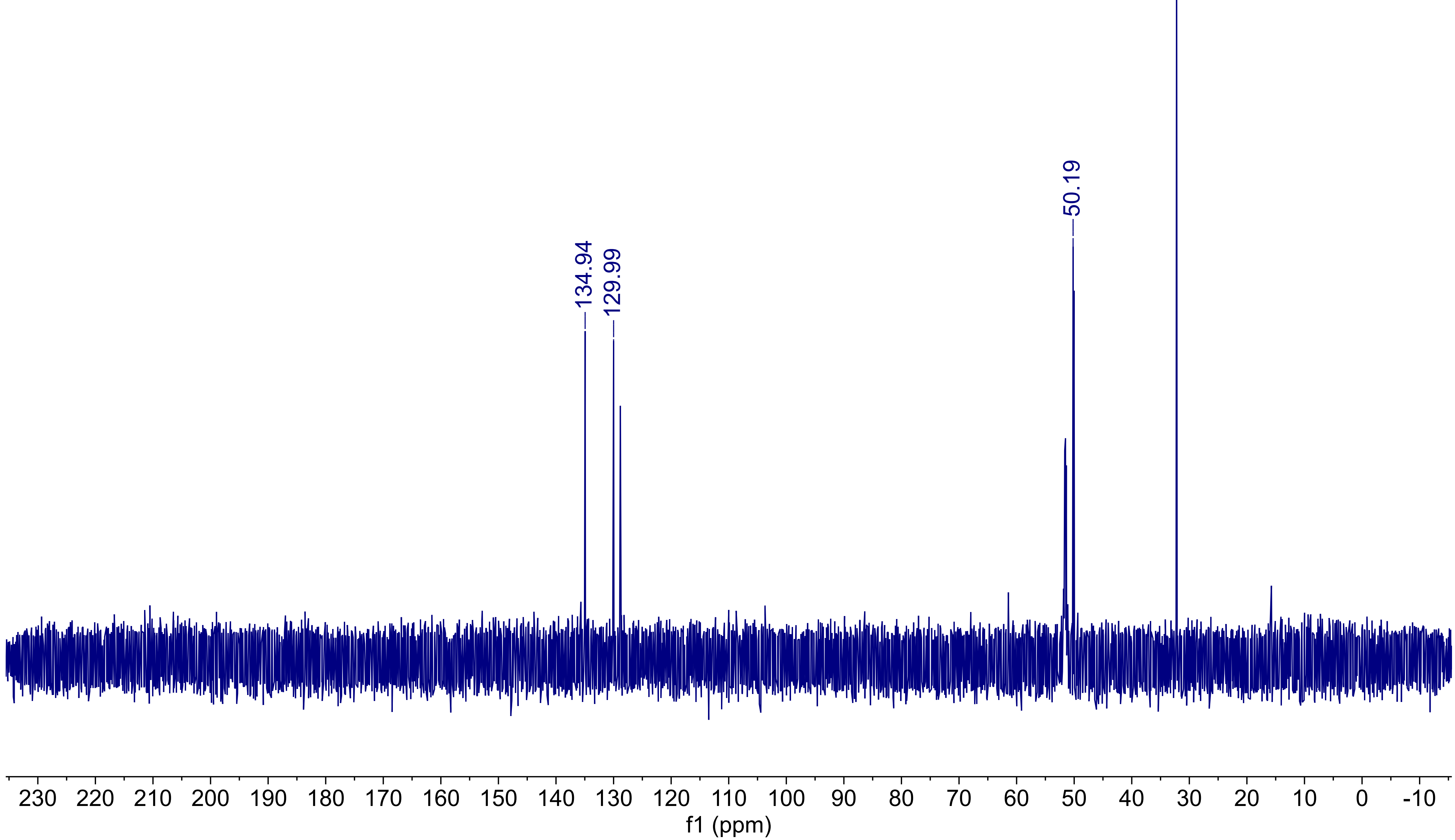

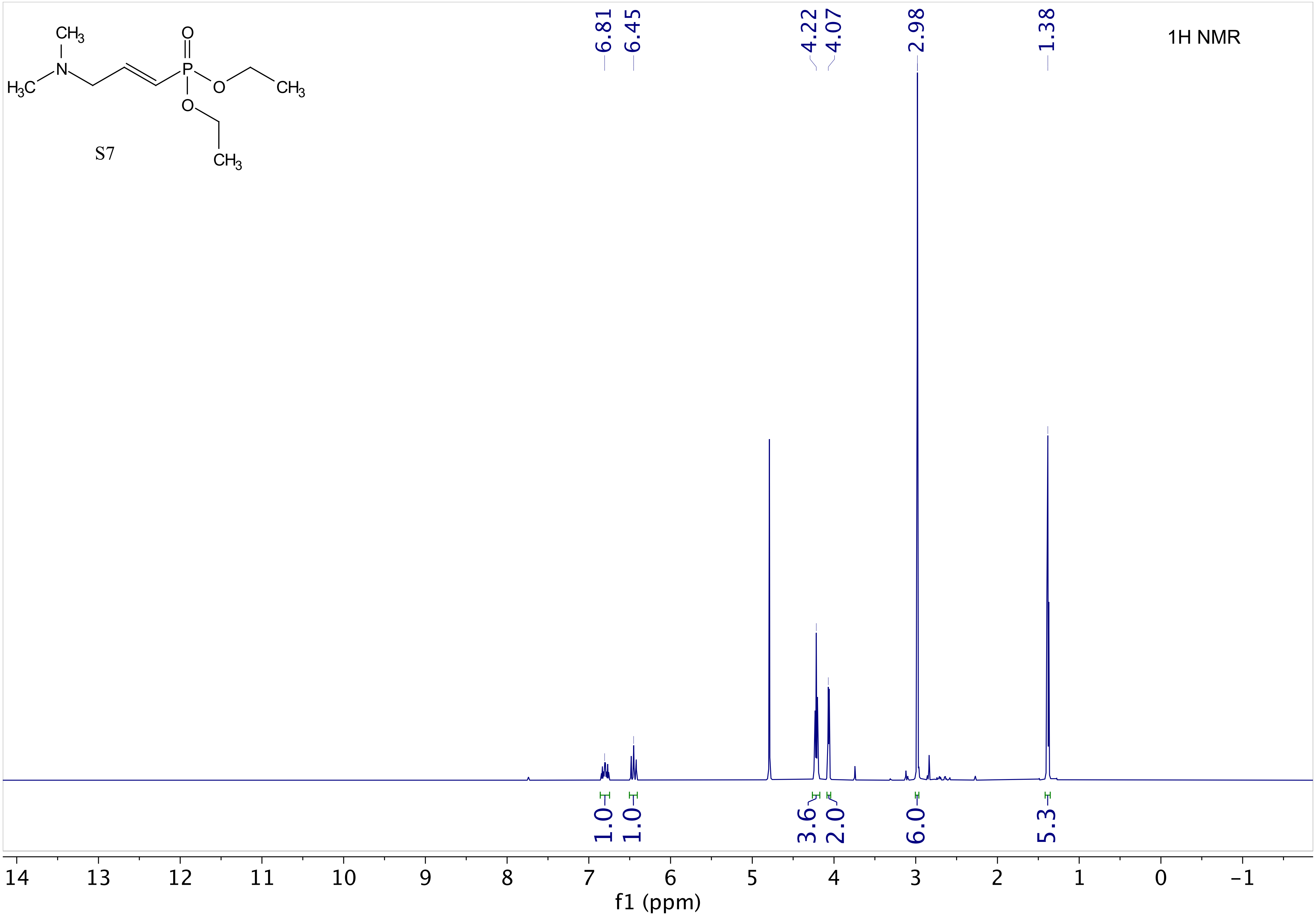
S7
1H NMR
6.81
6.45
4.22
4.07
2.98
1.38
1.0
1.0
3.6
2.0
6.0
5.3
14 13 12 11 10 9 8 7 6 5 4 3 2 1 0 −1
f1 (ppm)

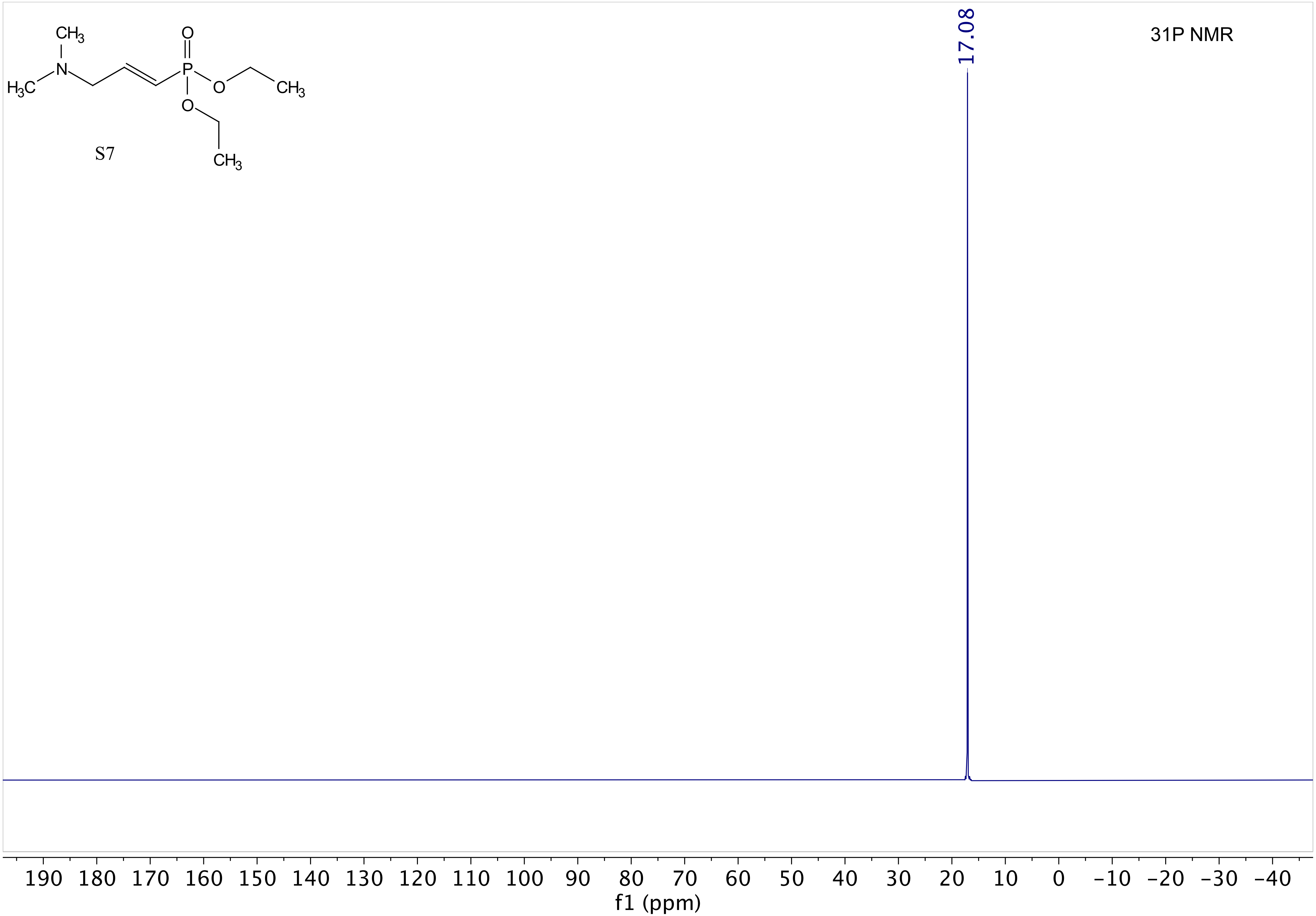

CH3
N
H3C
O
P
O
CH3
O
CH3
S7
17.08
31P NMR
190 180 170 160 150 140 130 120 110 100 90 80 70 60 50 40 30 20 10 0 −10 −20 −30 −40
f1 (ppm)

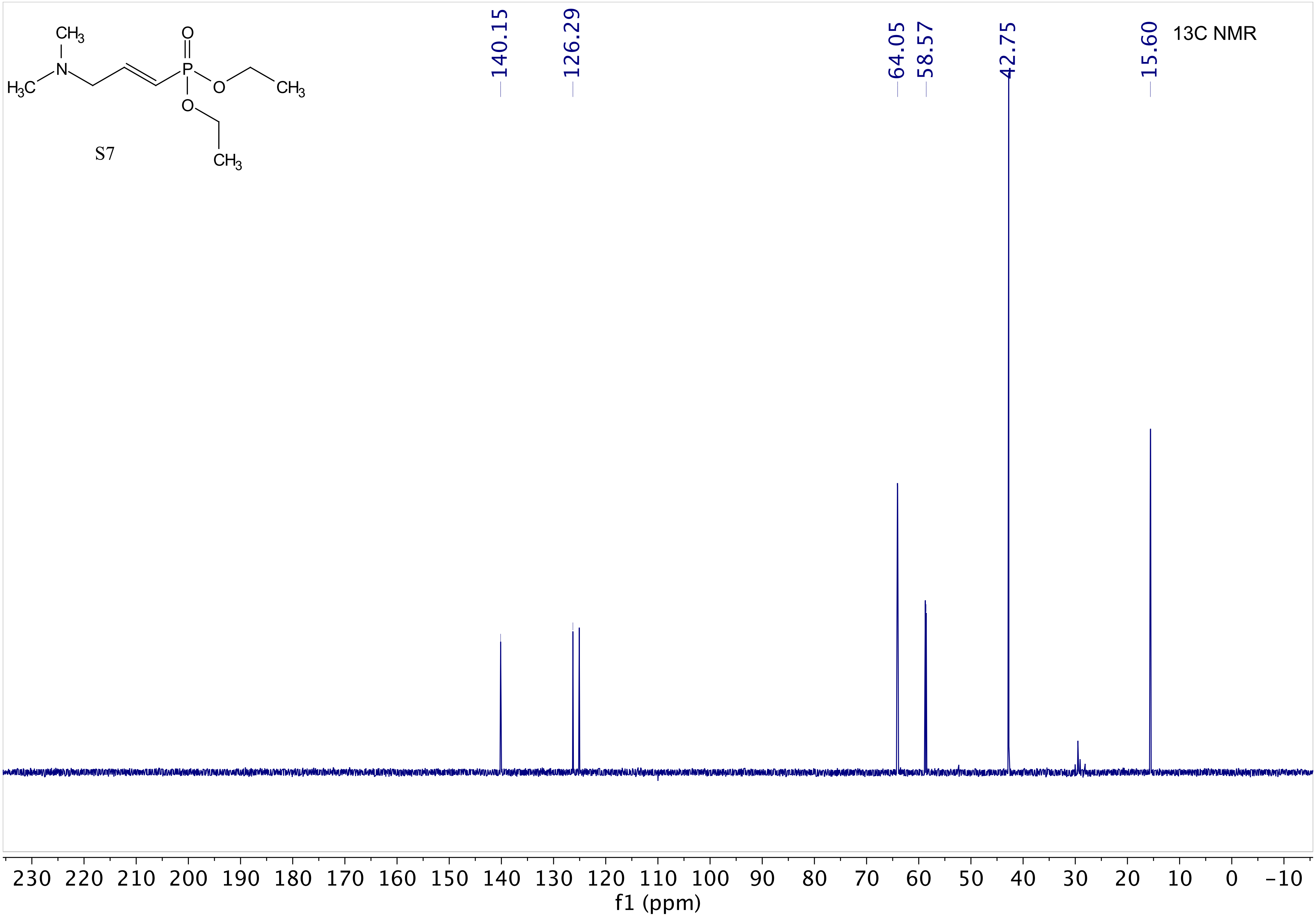

S7
140.15
126.29
64.05
58.57
42.75
15.60
13C NMR
230 220 210 200 190 180 170 160 150 140 130 120 110 100 90 80 70 60 50 40 30 20 10 0 −10
f1 (ppm)

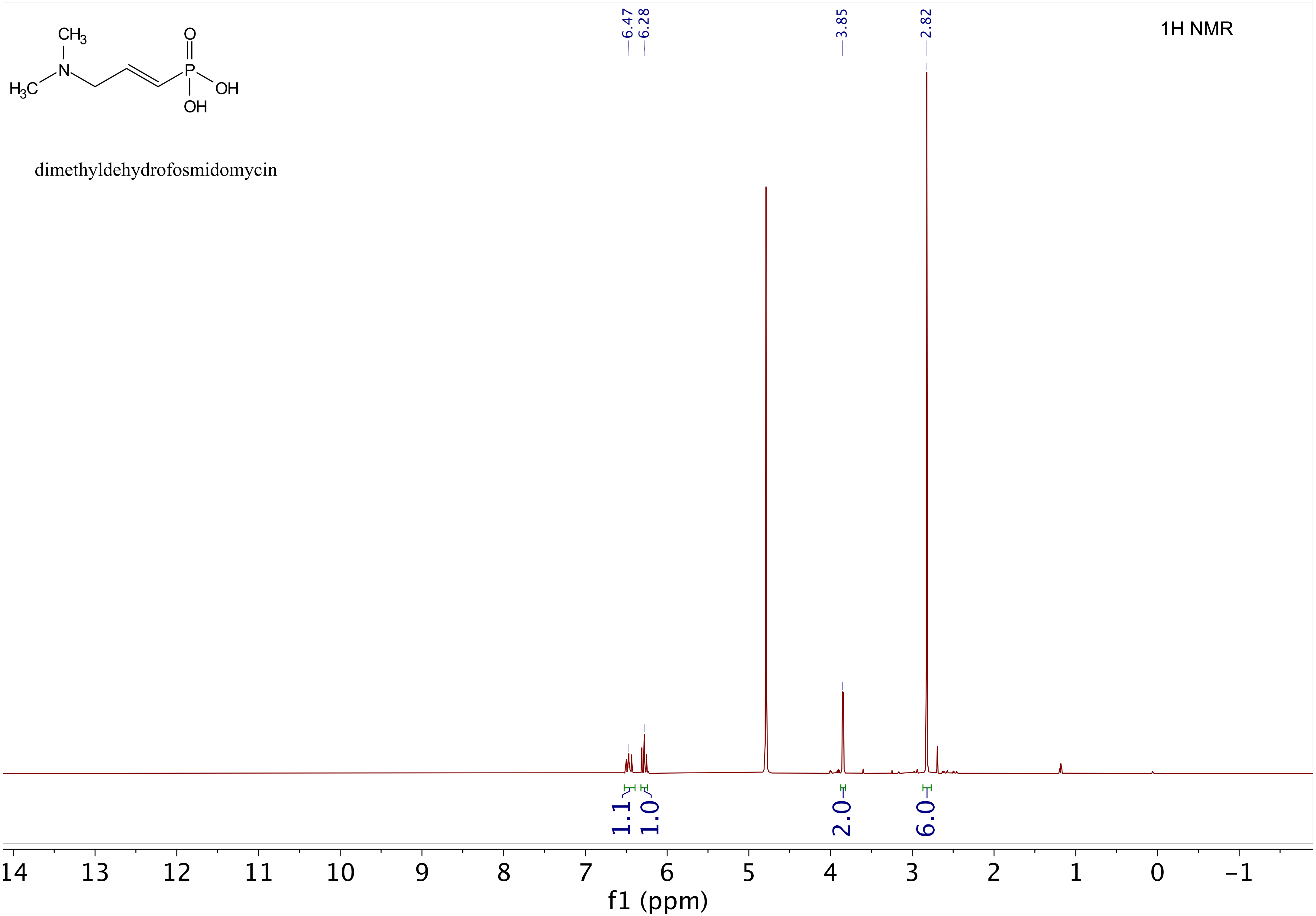

CH3
N
H3C
O
P
OH
OH
dimethyldehydrofosmidomycin
1H NMR
6.47
6.28
3.85
2.82
1.1
1.0
2.0
6.0
14
13
12
11
10
9
8
7
6
5
4
3
2
1
0
–1
f1 (ppm)

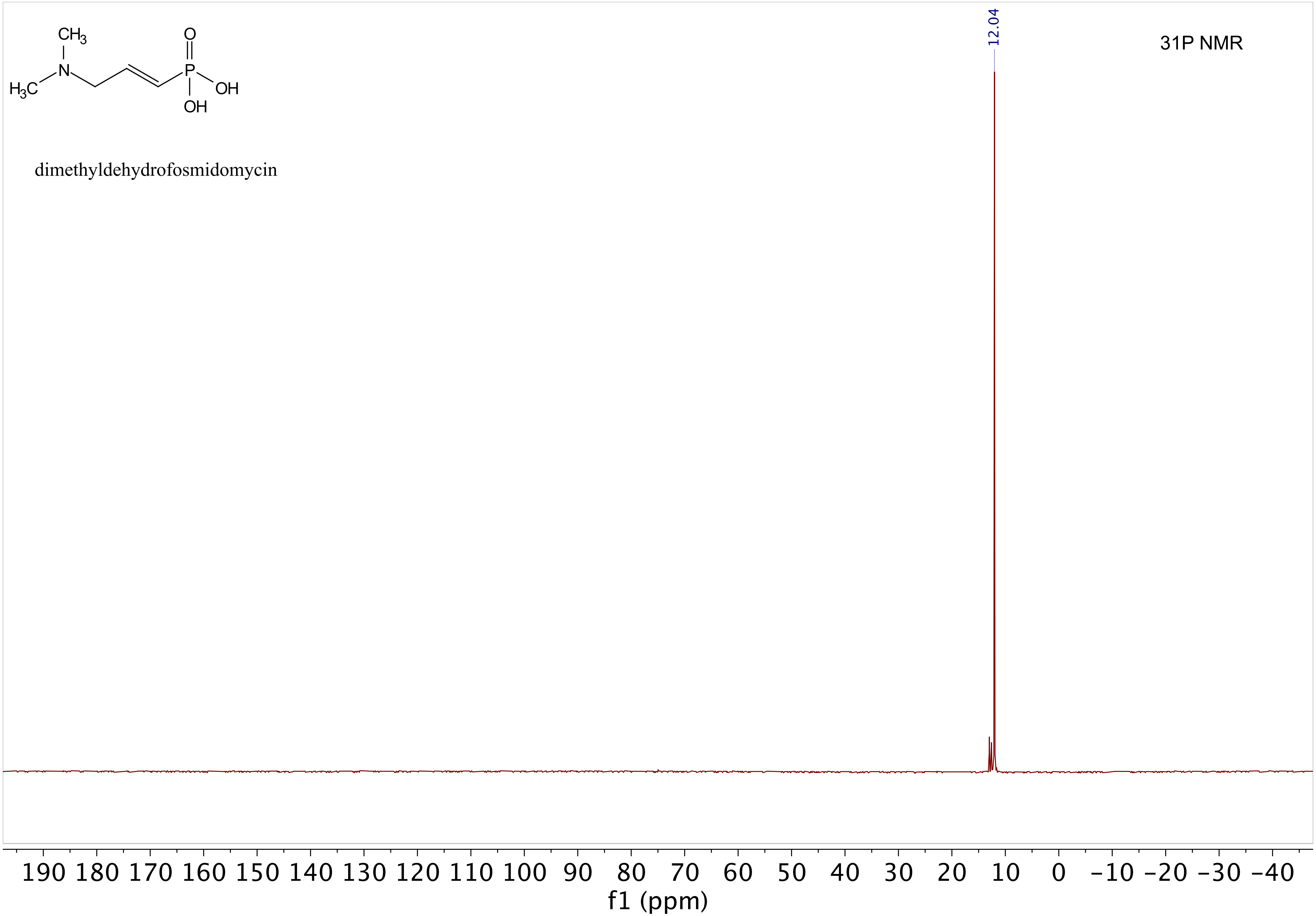

CH3
O
H3C
N
P
OH
OH
dimethyldehydrofosmidomycin
12.04
31P NMR
190 180 170 160 150 140 130 120 110 100 90 80 70 60 50 40 30 20 10 0 −10 −20 −30 −40
f1 (ppm)

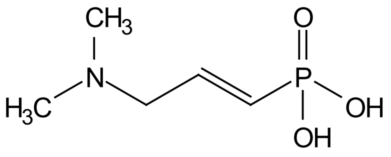

dimethyldehydrofosmidomycin

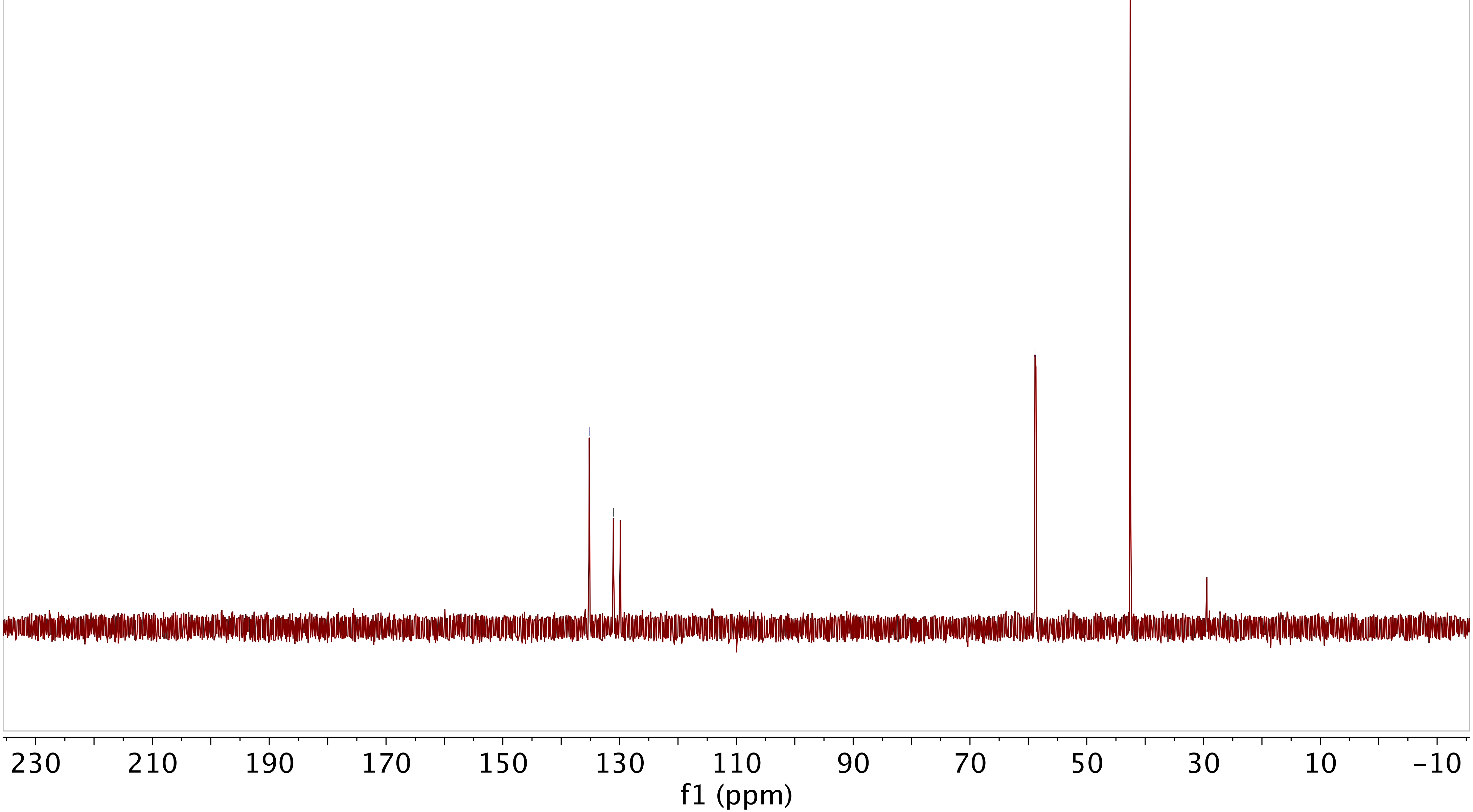

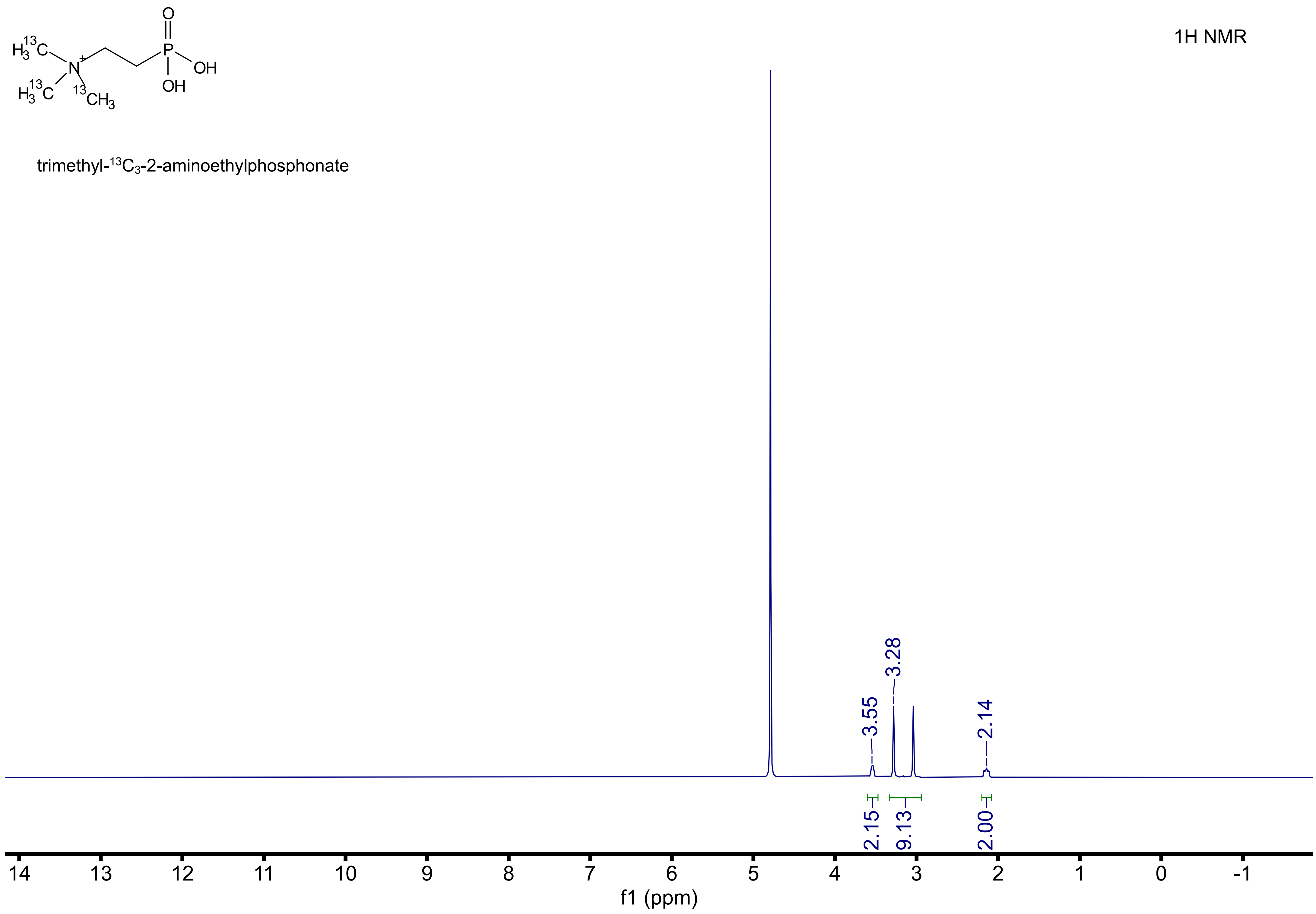
1H NMR
trimethyl-13C3-2-aminoethylphosphonate
OH
OH
O
P
N+
13CH3
H3 13C
H3 13C
3.55
3.28
2.14
2.15
9.13
2.00
14 13 12 11 10 9 8 7 6 5 4 3 2 1 0 -1
f1 (ppm)

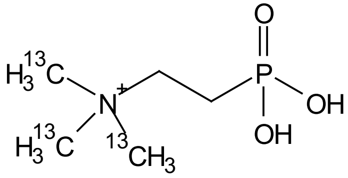

trimethyl-$^{13}C_3$-2-aminoethylphosphonate

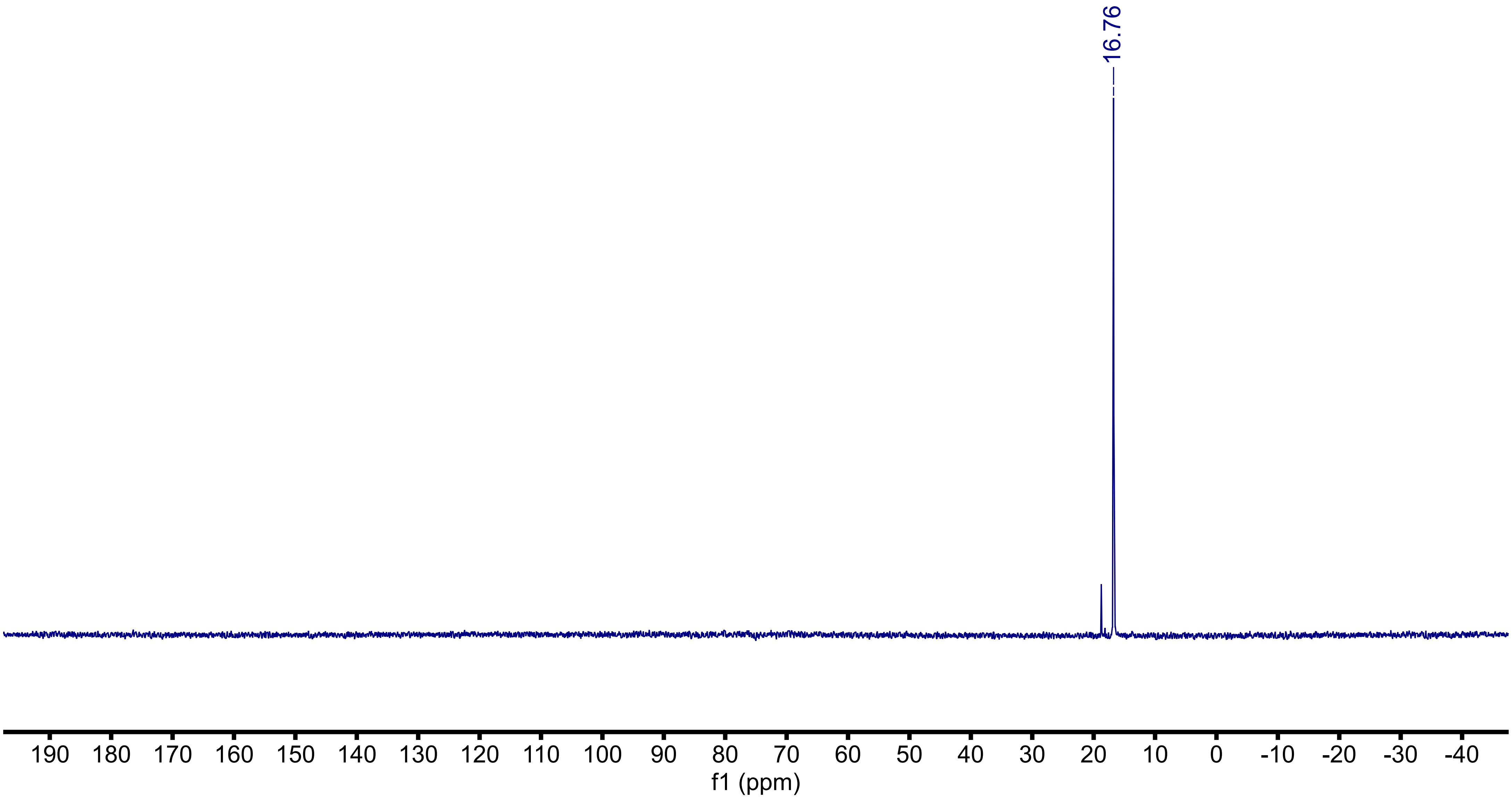

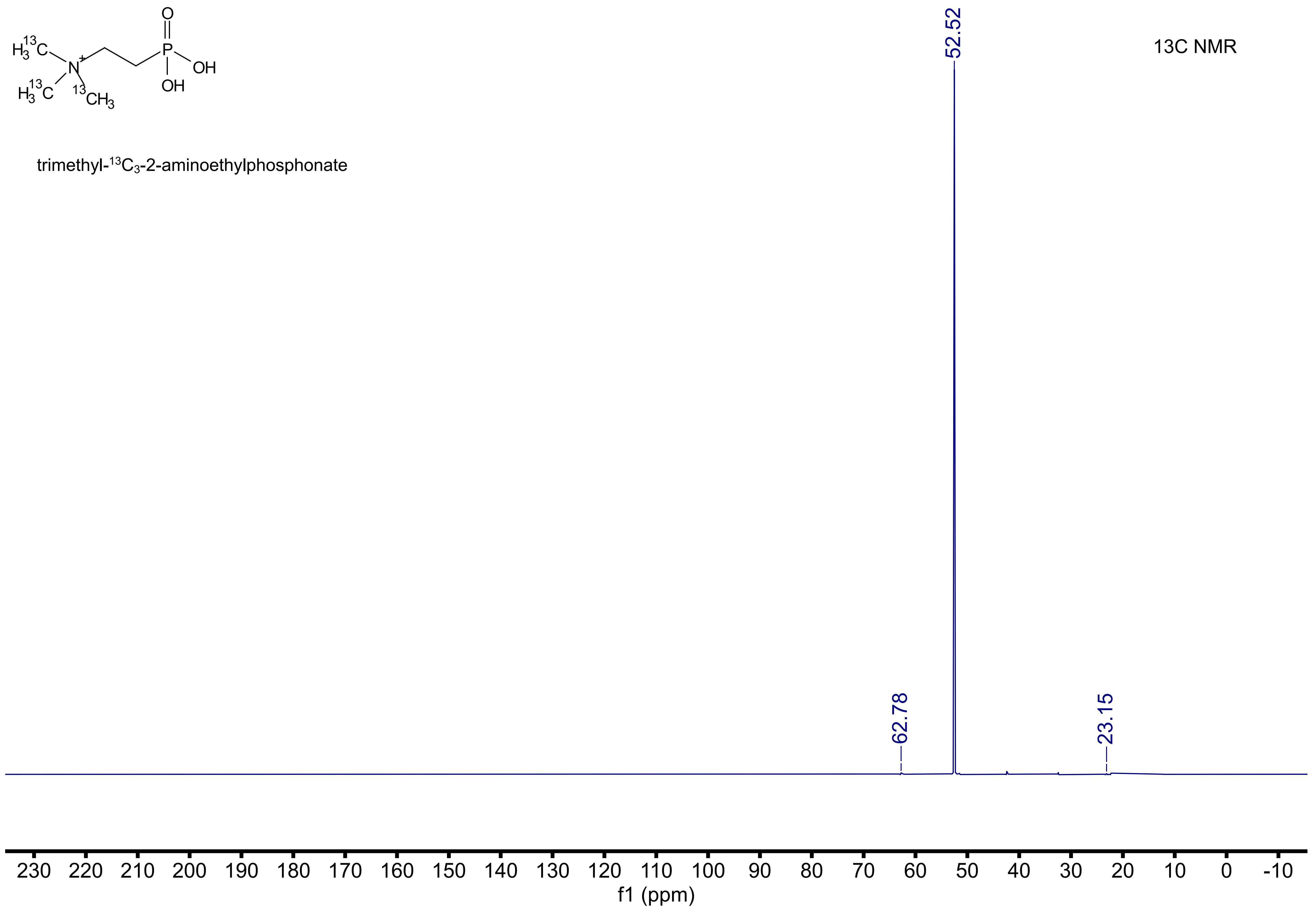

trimethyl-13C3-2-aminoethylphosphonate
13C NMR
52.52
62.78
23.15
230 220 210 200 190 180 170 160 150 140 130 120 110 100 90 80 70 60 50 40 30 20 10 0 -10
f1 (ppm)

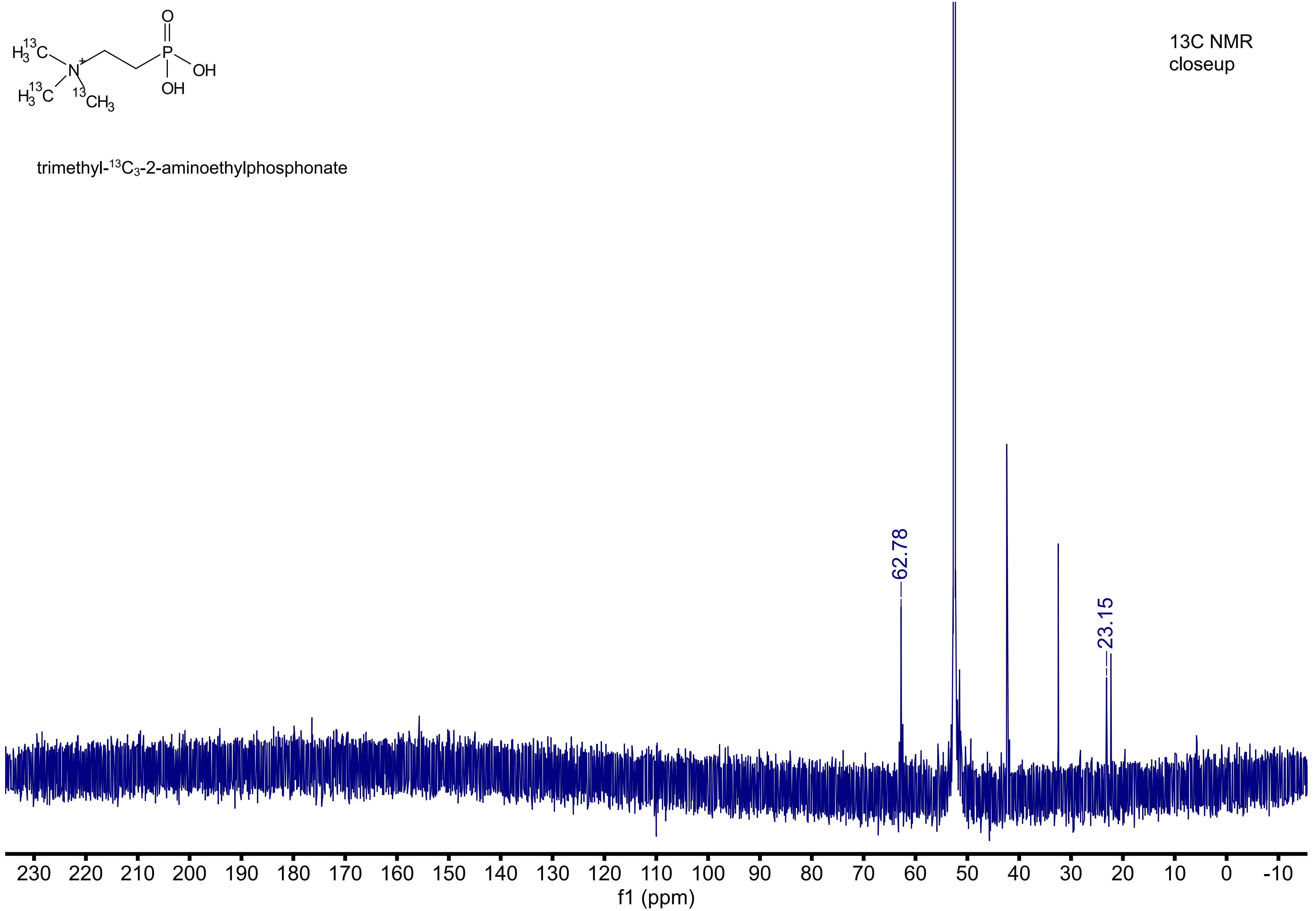

O
P
OH
OH
N
+
$H_3{}^{13}C$
$H_3{}^{13}C$
$^{13}CH_3$
trimethyl-$^{13}C_3$-2-aminoethylphosphonate
13C NMR
closeup
62.78
23.15
230 220 210 200 190 180 170 160 150 140 130 120 110 100 90 80 70 60 50 40 30 20 10 0 -10
f1 (ppm)

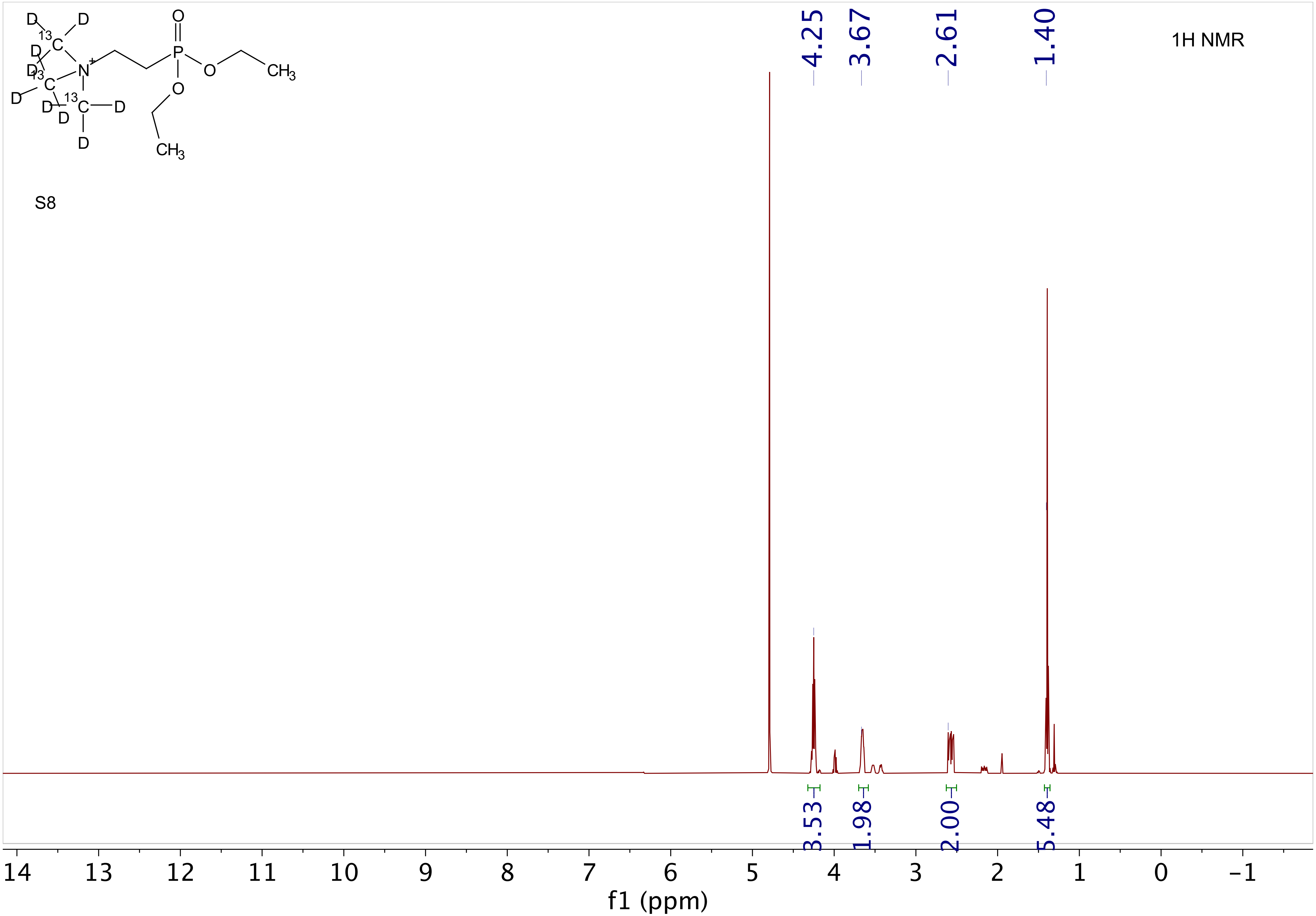
S8
1H NMR
4.25
3.67
2.61
1.40
3.53
1.98
2.00
5.48
14 13 12 11 10 9 8 7 6 5 4 3 2 1 0 −1
f1 (ppm)

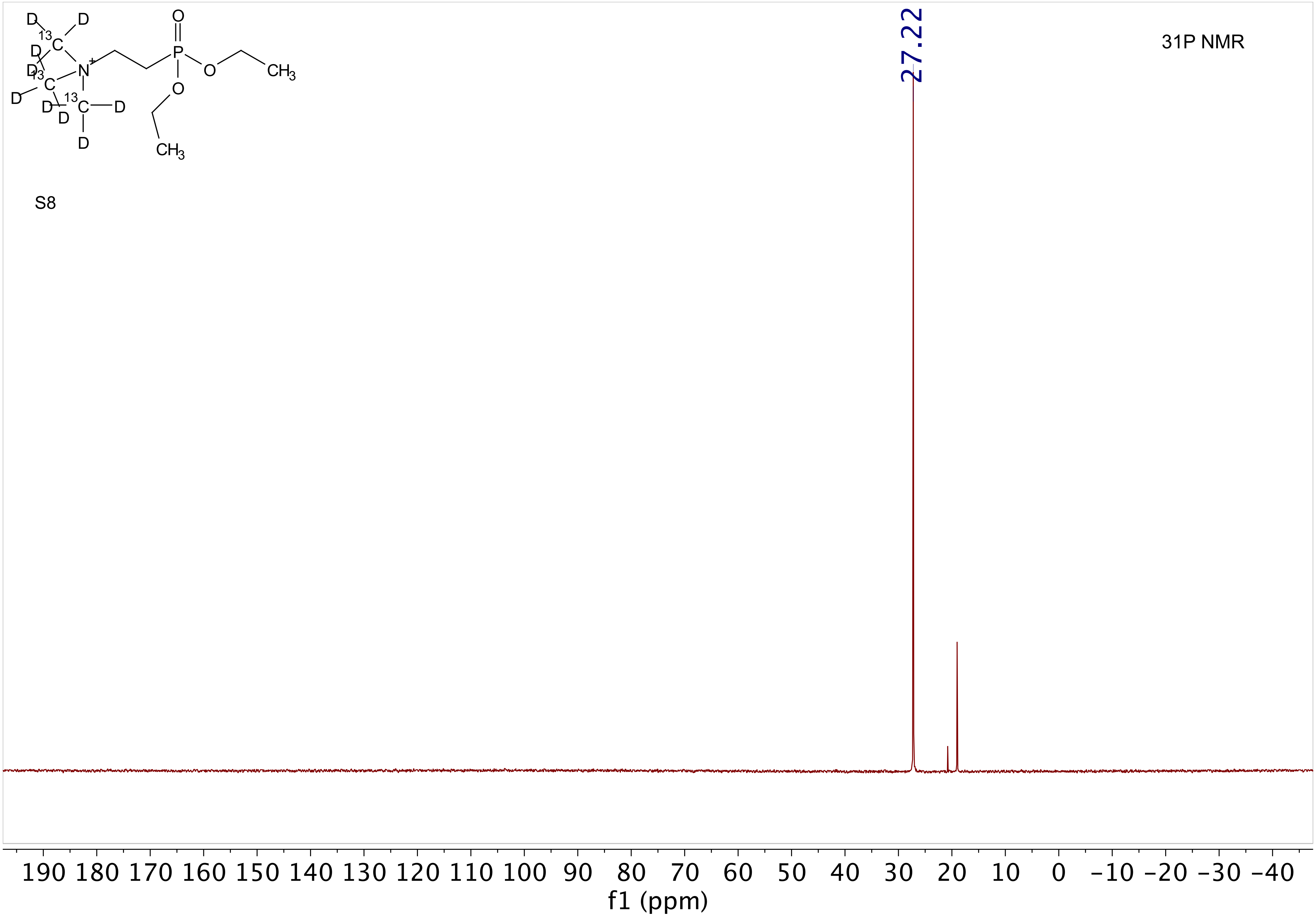
S8
31P NMR
27.22
190 180 170 160 150 140 130 120 110 100 90 80 70 60 50 40 30 20 10 0 −10 −20 −30 −40
f1 (ppm)

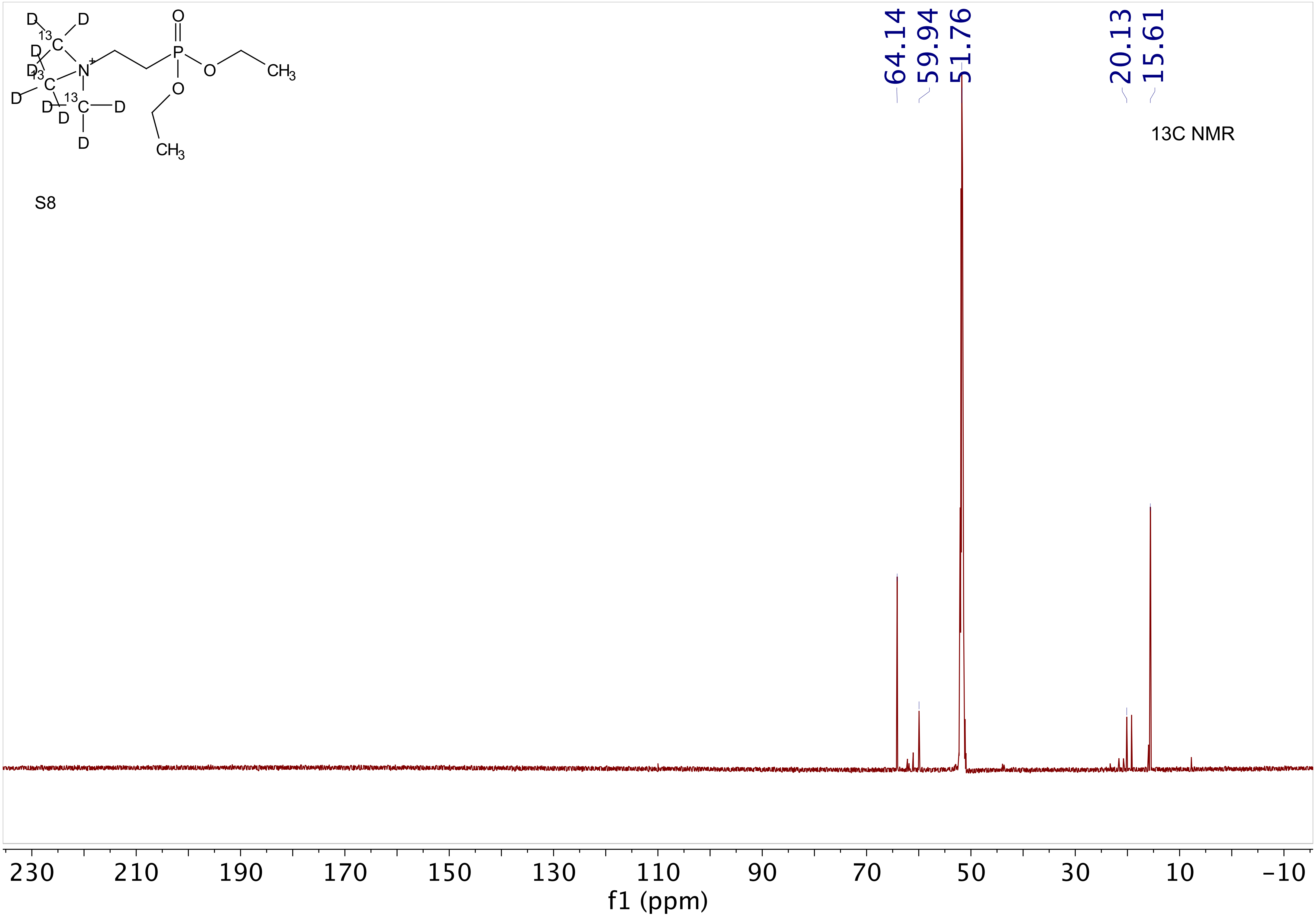
S8
64.14
59.94
51.76
20.13
15.61
13C NMR
230 210 190 170 150 130 110 90 70 50 30 10 −10
f1 (ppm)

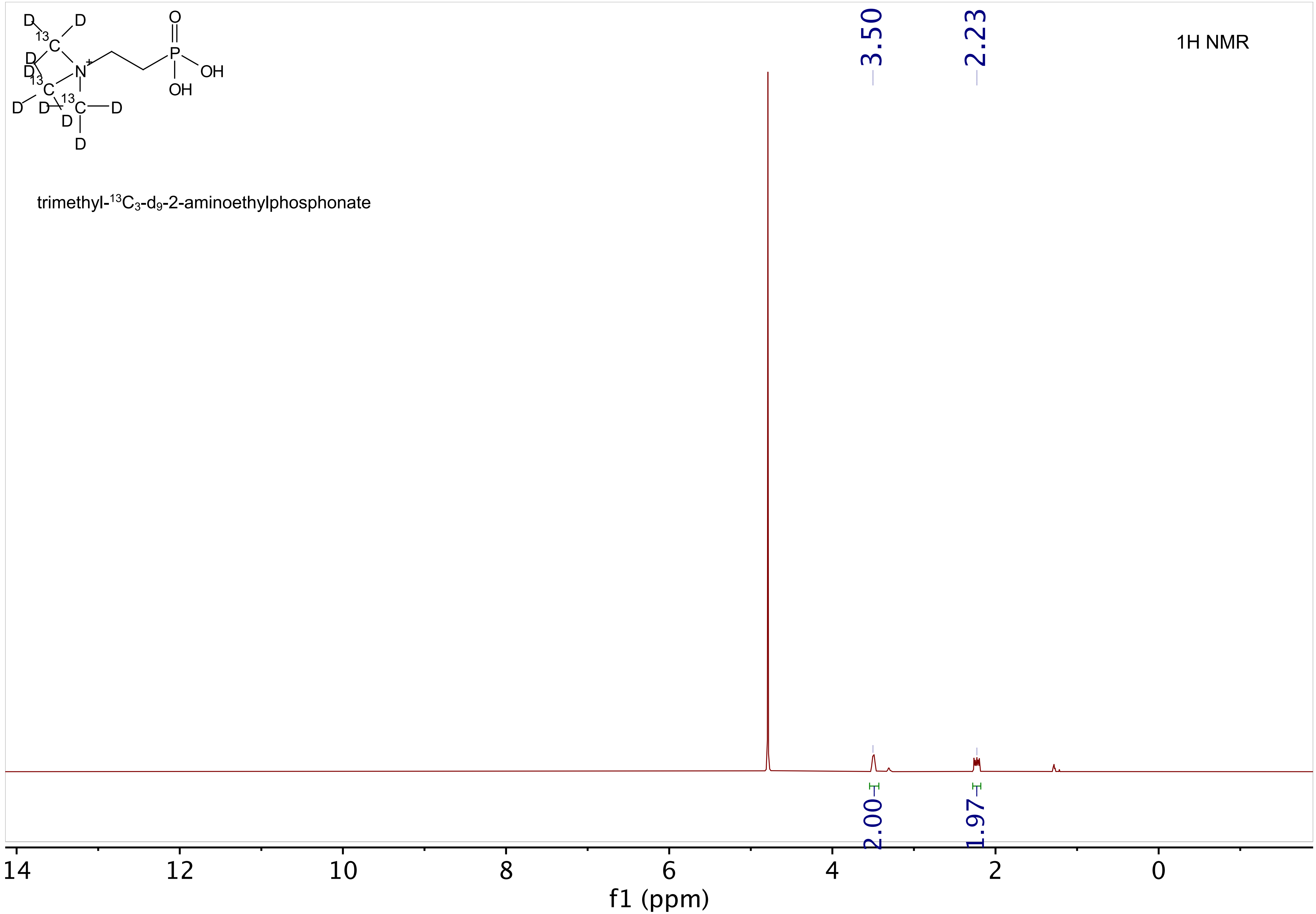
1H NMR
trimethyl-$^{13}C_3$-d_9-2-aminoethylphosphonate
3.50
2.23
2.00
1.97
f1 (ppm)

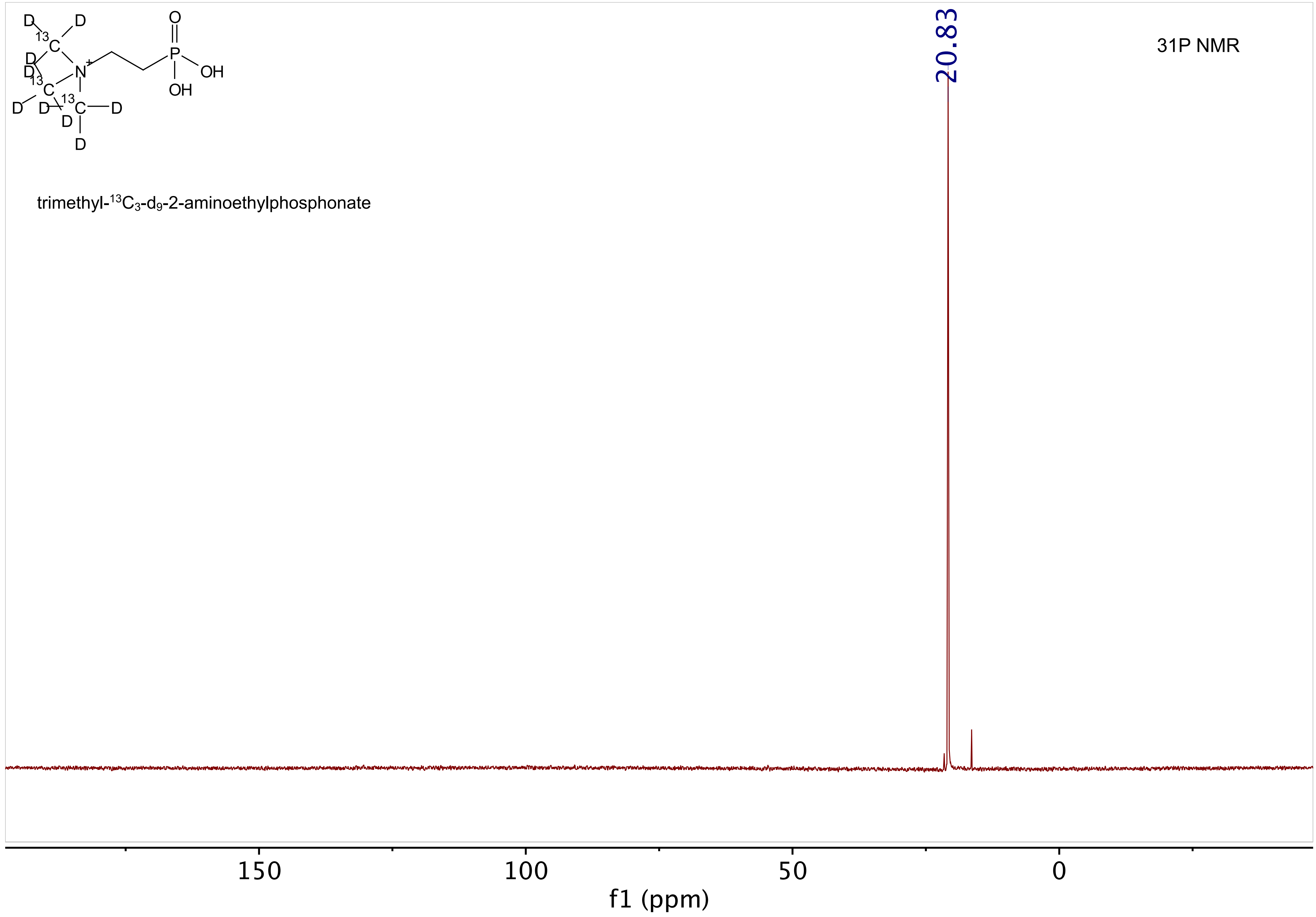

31P NMR
20.83
trimethyl-13C3-d9-2-aminoethylphosphonate
150
100
50
0
f1 (ppm)

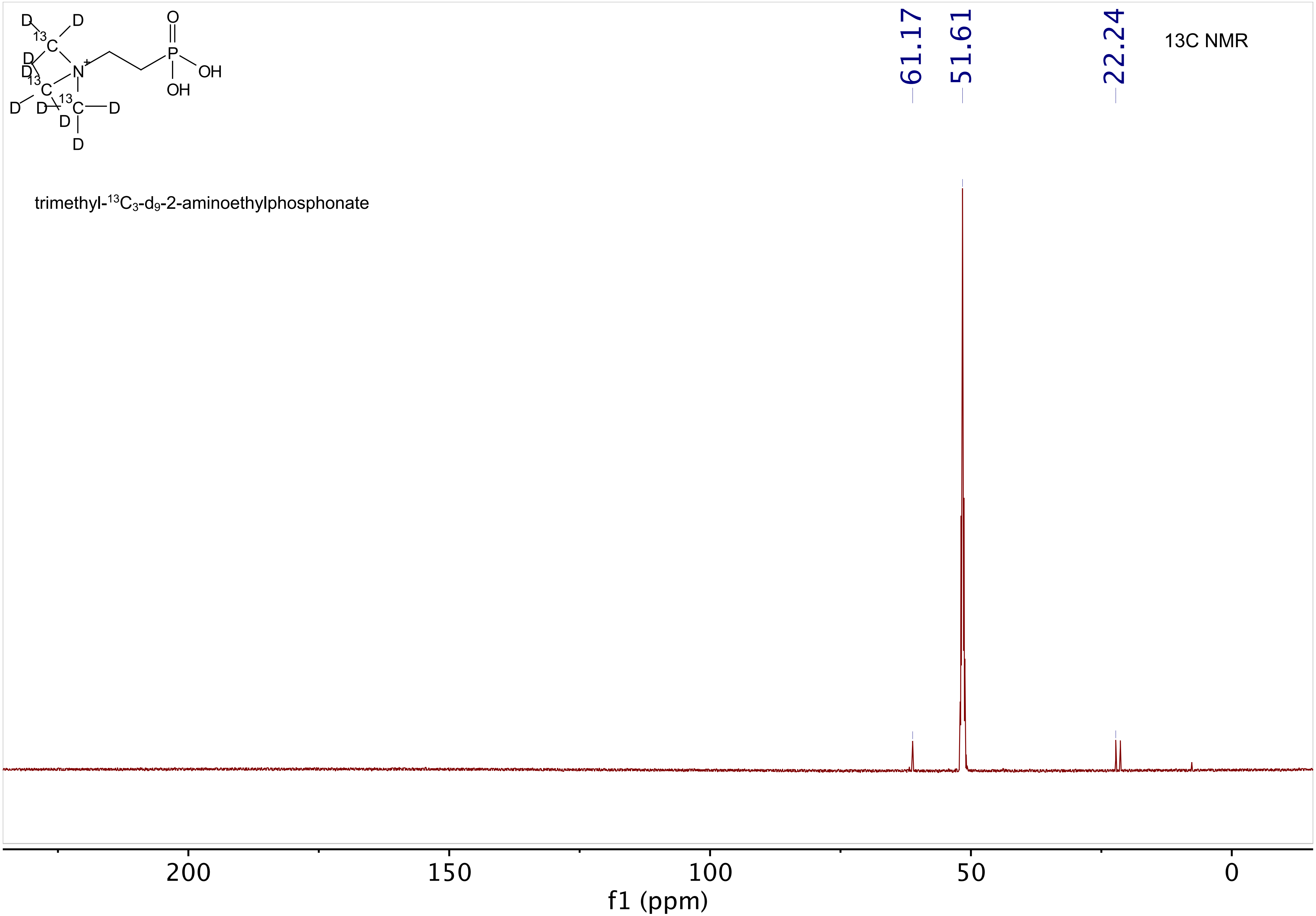

trimethyl-13C3-d9-2-aminoethylphosphonate
13C NMR
61.17
51.61
22.24
200
150
100
50
0
f1 (ppm)

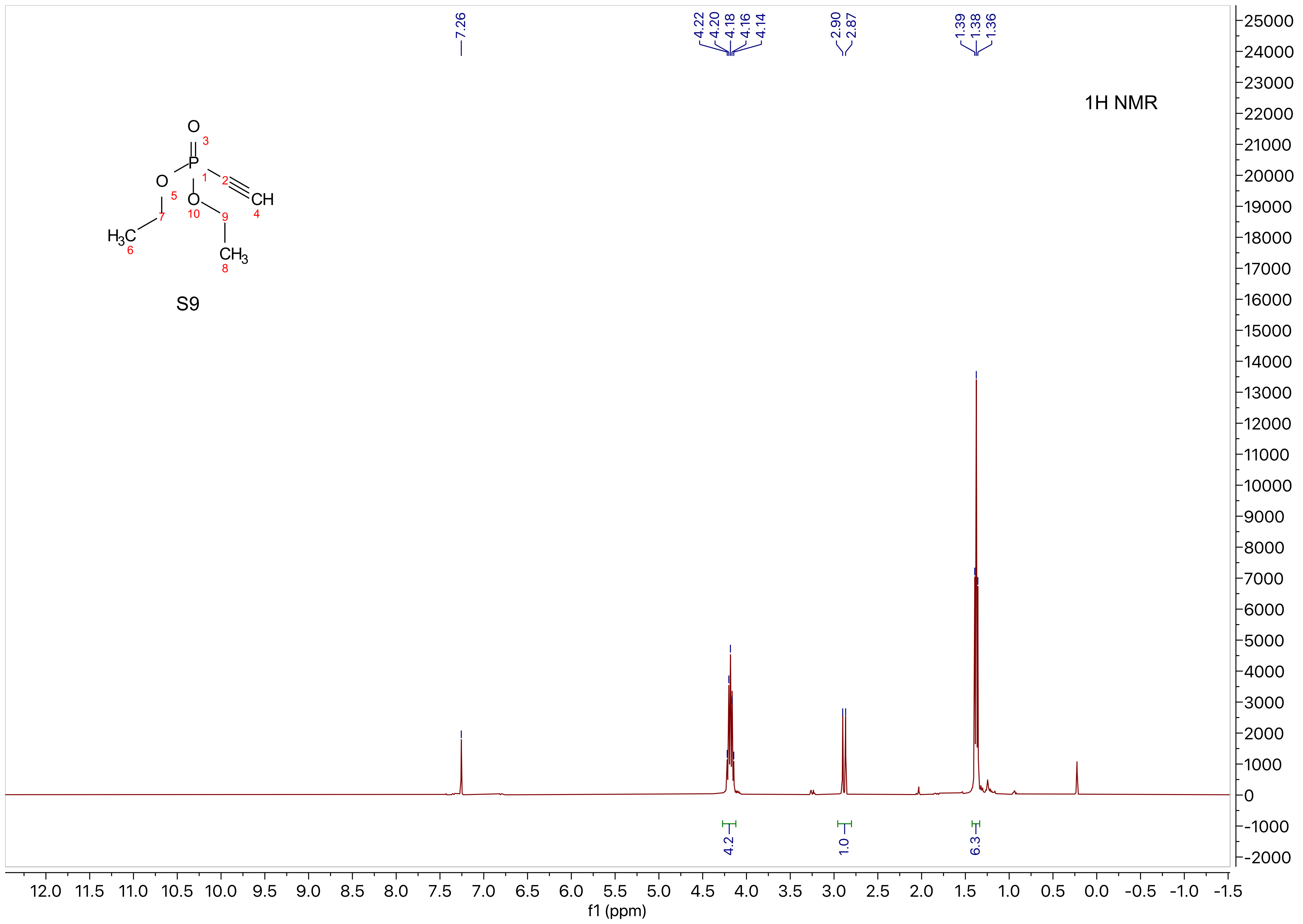
1H NMR
S9
7.26
4.22
4.20
4.18
4.16
4.14
2.90
2.87
1.39
1.38
1.36
4.2
1.0
6.3
f1 (ppm)

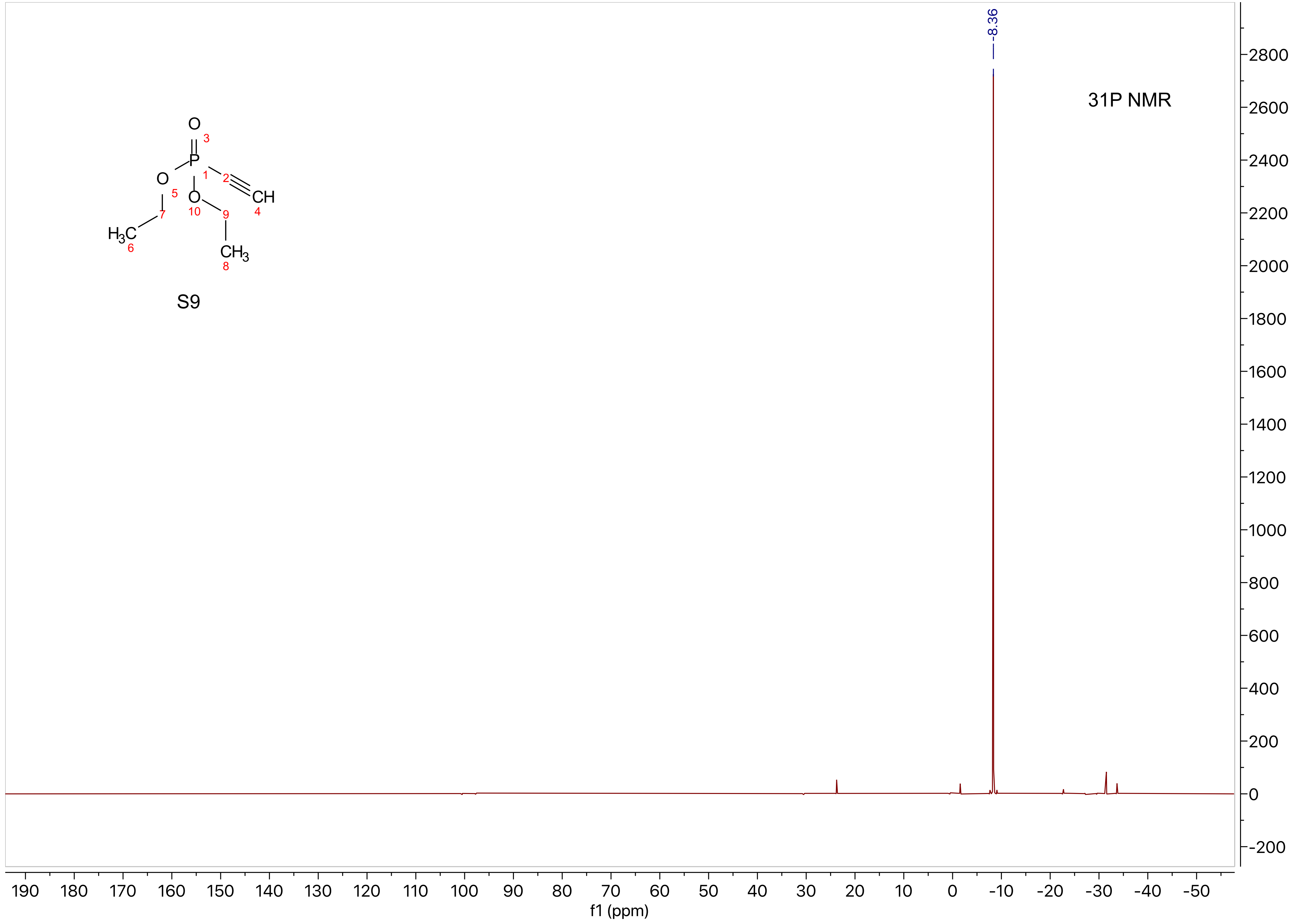

-8.36
31P NMR
S9
f1 (ppm)

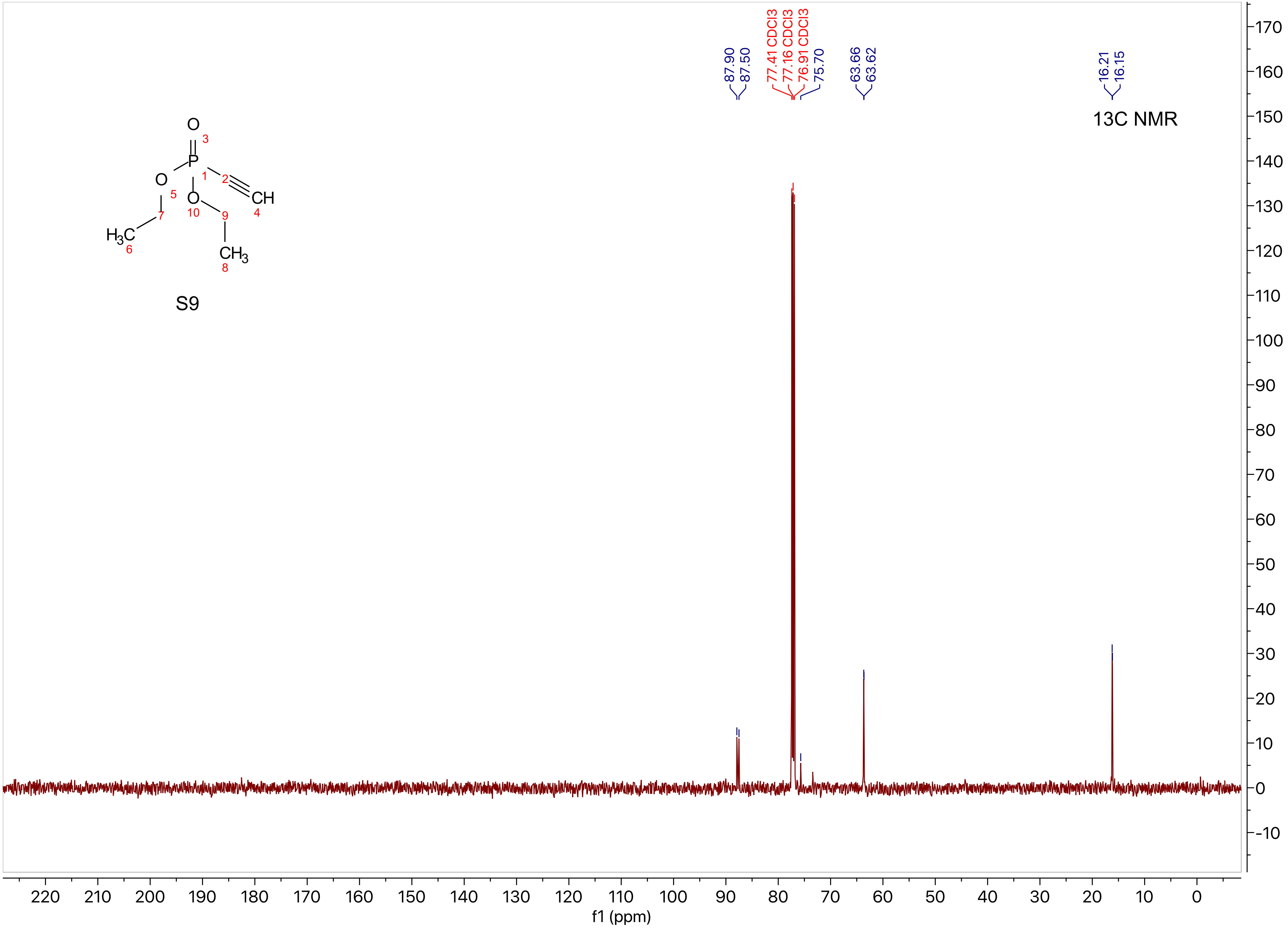
87.90
87.50
77.41 CDCl3
77.16 CDCl3
76.91 CDCl3
75.70
63.66
63.62
16.21
16.15
13C NMR
O
3
P
1
2
O
5
O
10
CH
4
7
9
H3C
6
CH3
8
S9
220
210
200
190
180
170
160
150
140
130
120
110
100
90
80
70
60
50
40
30
20
10
0
f1 (ppm)
170
160
150
140
130
120
110
100
90
80
70
60
50
40
30
20
10
0
-10

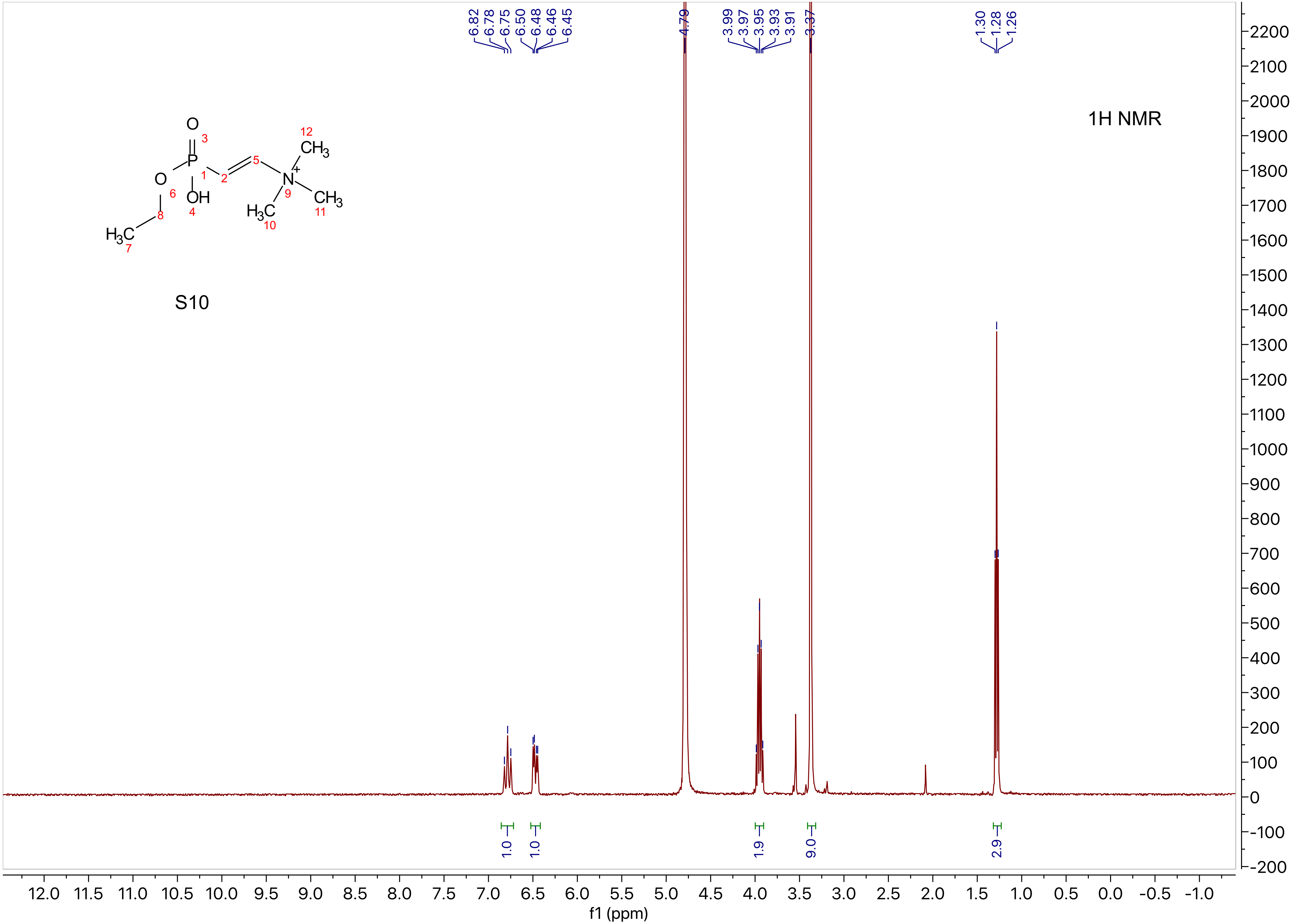

1H NMR
S10
6.82
6.78
6.75
6.50
6.48
6.46
6.45
4.79
3.99
3.97
3.95
3.93
3.91
3.37
1.30
1.28
1.26
1.0
1.0
1.9
9.0
2.9
f1 (ppm)

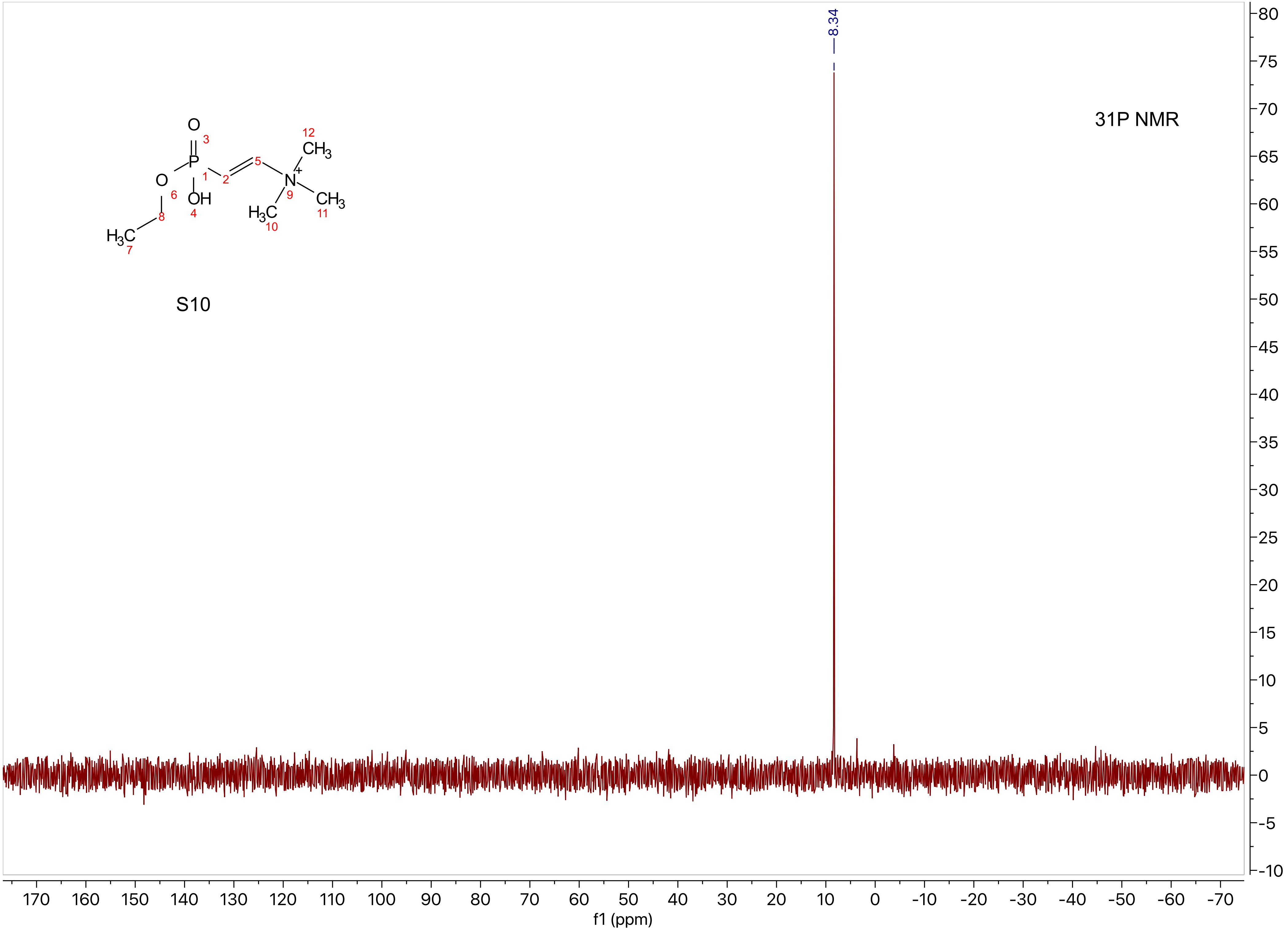

S10
8.34
31P NMR
f1 (ppm)

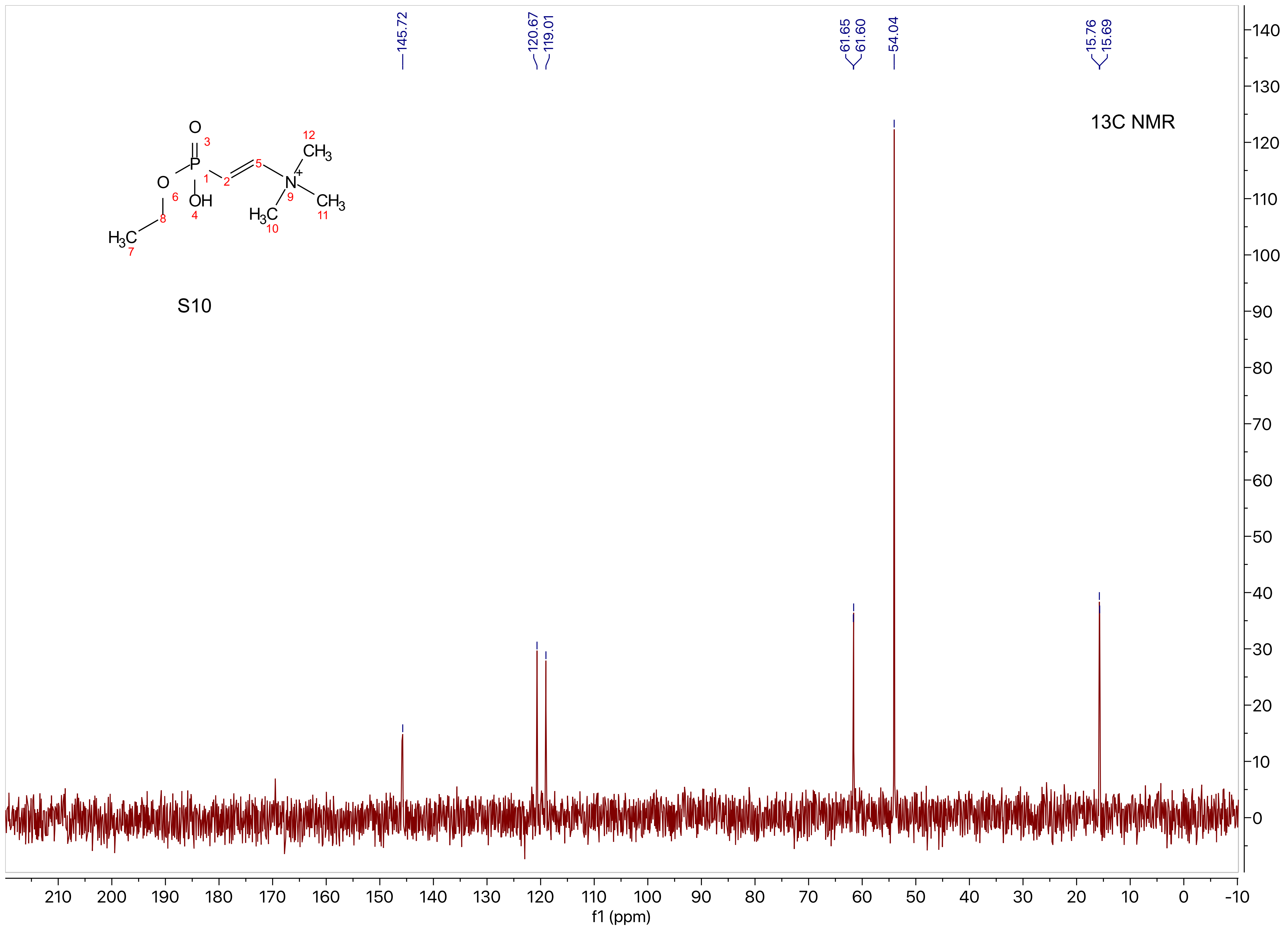
145.72
120.67
119.01
61.65
61.60
54.04
15.76
15.69
13C NMR
S10
f1 (ppm)

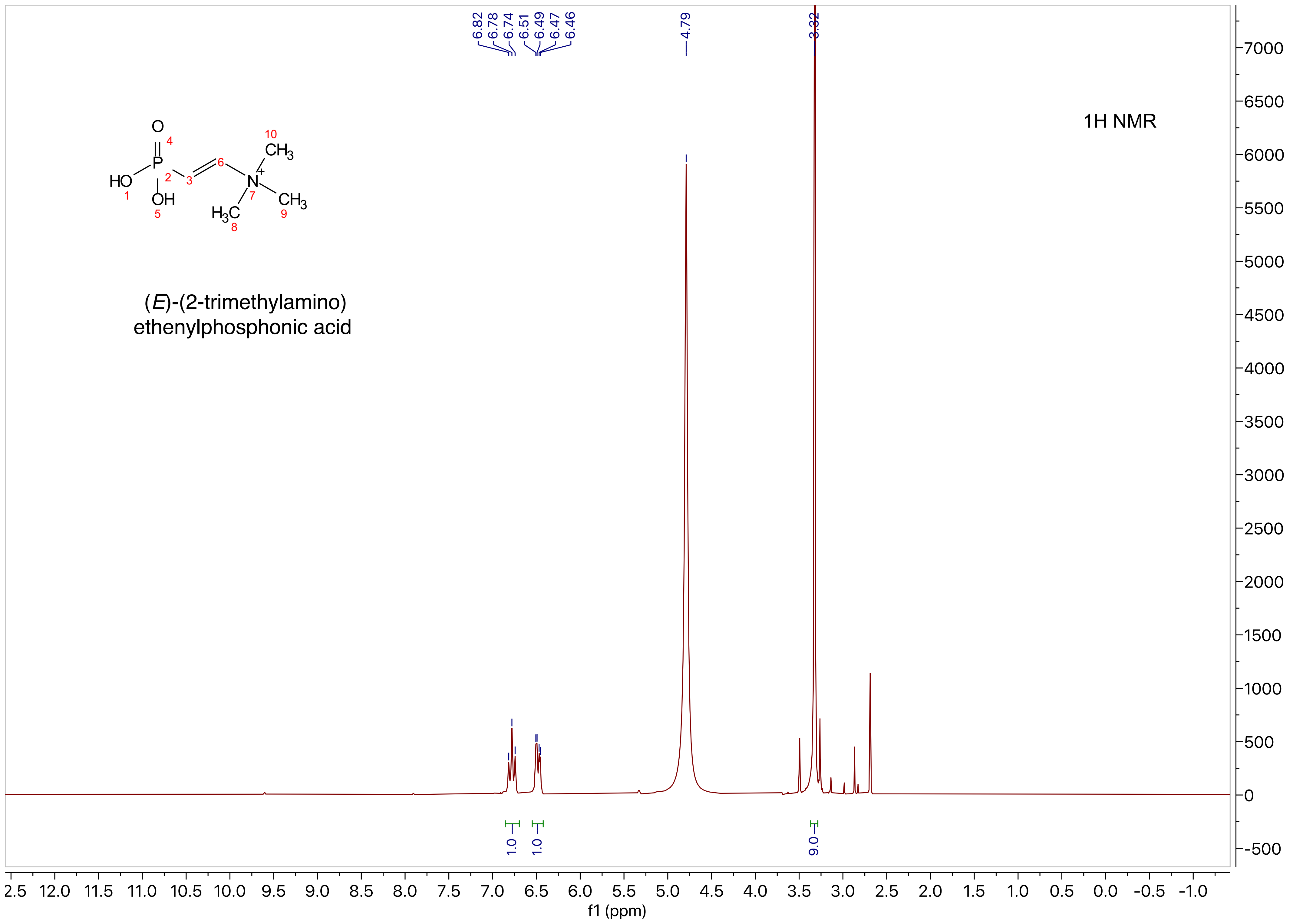

1H NMR
(E)-(2-trimethylamino) ethenylphosphonic acid
O
4
P
HO
1
2
3
6
OH
5
N+
7
10
CH3
CH3
9
H3C
8
6.82
6.78
6.74
6.51
6.49
6.47
6.46
4.79
3.32
1.0
1.0
9.0
f1 (ppm)

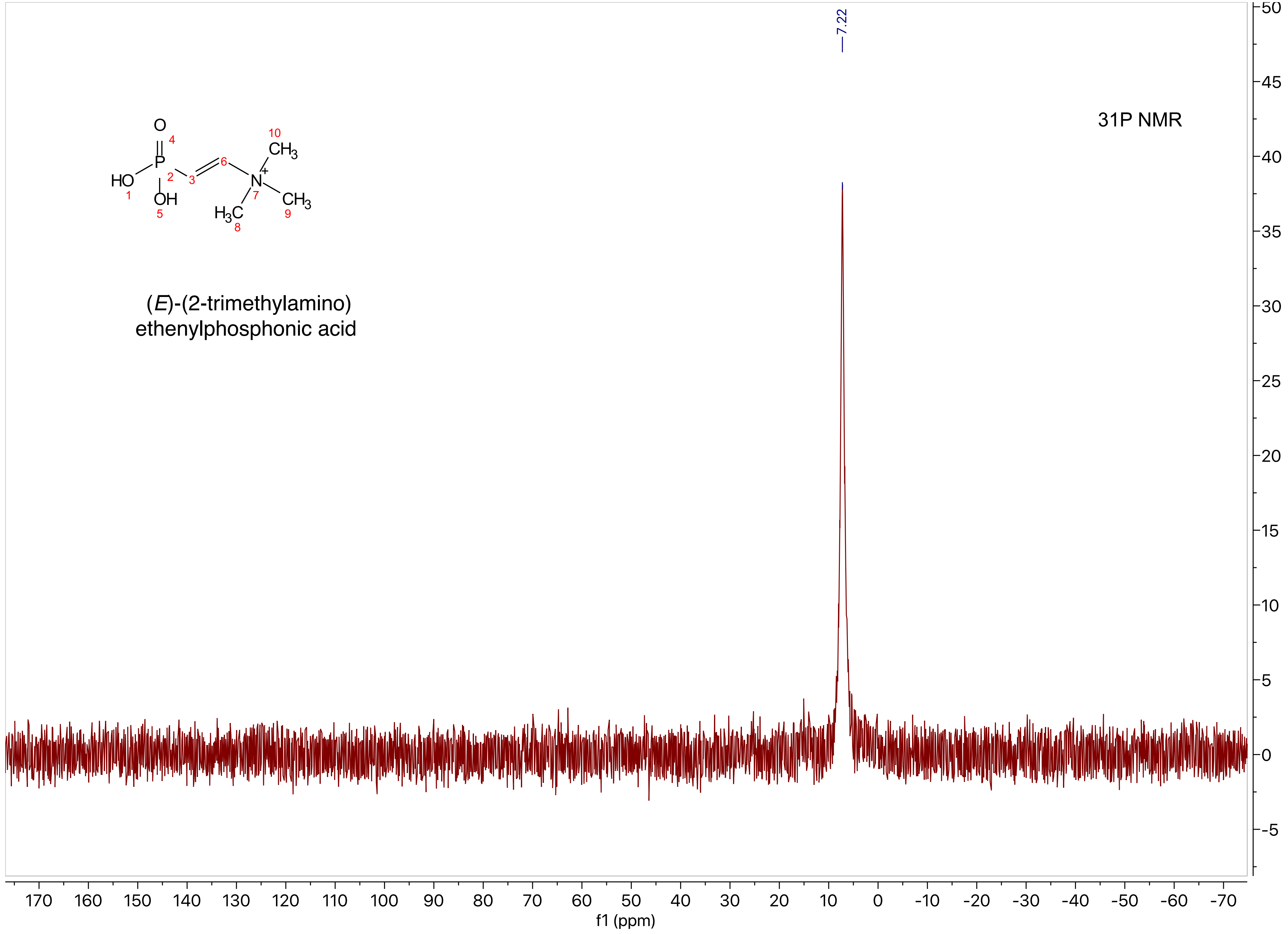

(E)-(2-trimethylamino)
ethenylphosphonic acid
31P NMR
7.22
f1 (ppm)

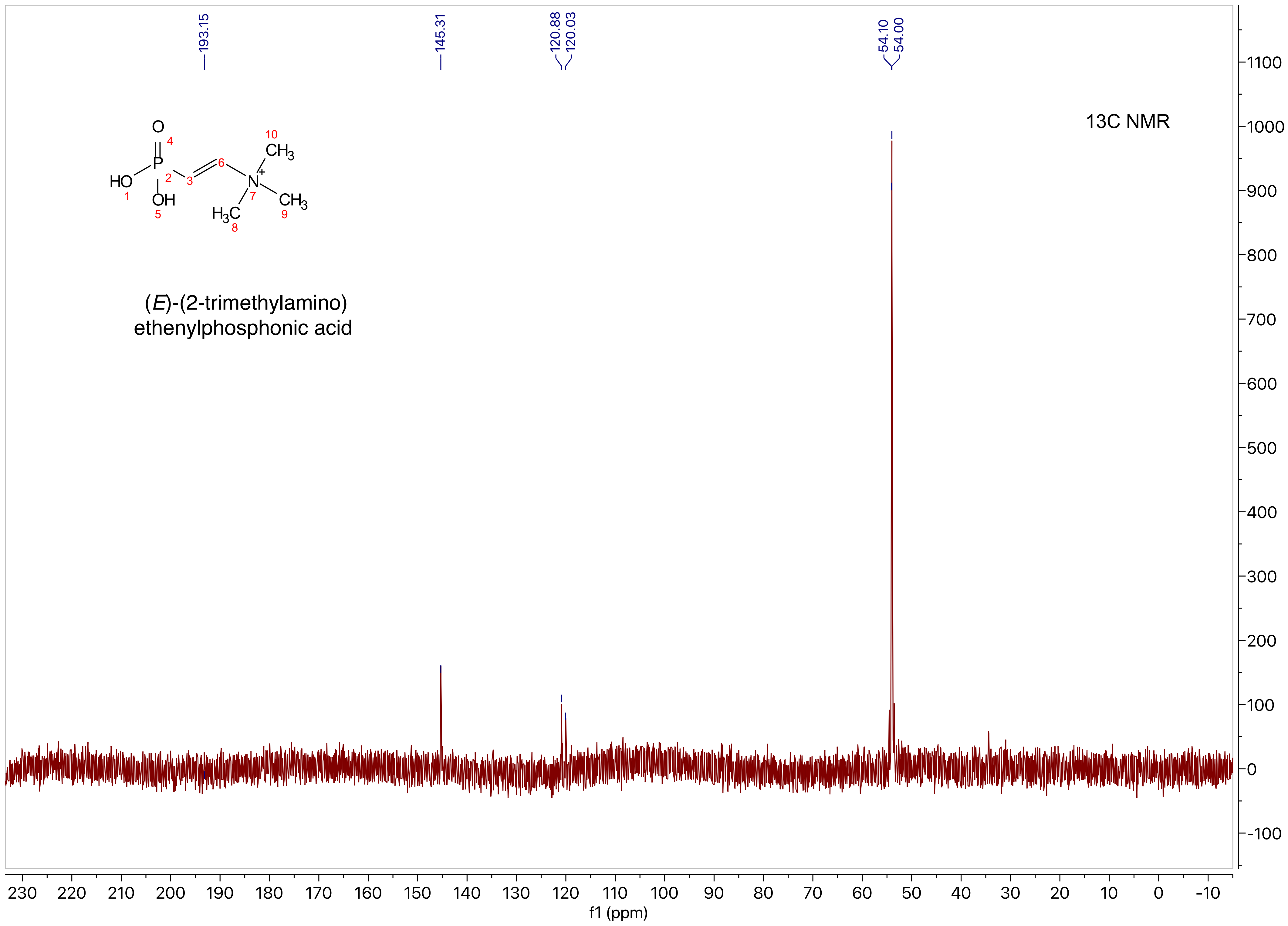
193.15
145.31
120.88
120.03
54.10
54.00
13C NMR
(E)-(2-trimethylamino)
ethenylphosphonic acid
f1 (ppm)
